# Whose morning, whose evening? Temporal asymmetries in intermedia agenda-setting between X and online news in Türkiye

**DOI:** 10.1371/journal.pone.0362255

**Published:** 2026-11-06

**Authors:** Mehmet Ceylan, Ramazan Yılmaz, Rumeysa Erdoğan Bodur

**Affiliations:** 1 Department of New Media and Communication, Faculty of Communication, Zonguldak Bülent Ecevit University, Zonguldak, Türkiye; 2 Department of Computer Technology and Information Systems, Faculty of Science, Bartın University, Bartın, Türkiye; PLoS ONE, UNITED STATES OF AMERICA

## Abstract

This study examines whether temporal ordering between X and online news varies across first-level issue and second-level attribute agendas in Türkiye’s polarized media environment. The analysis draws on 448,664 X posts and 10,357 articles from 19 mainstream online news outlets collected during the 31-day period preceding and including the 31 March 2024 Istanbul local election. Using 12-hour AM/PM windows, we conducted 122 cross-lagged panel analyses—62 same-day AM → PM and 60 overnight PM→next-day AM panels across the two agenda levels—comparing issue- and attribute-indicator frequency profiles. First-level issue agendas were strongly synchronized, with correlations often approaching or exceeding r = .90, whereas second-level attribute relationships were weaker and less consistent. At the first level, morning online news issue salience more often preceded later X issue salience within same-day transitions, whereas evening X issue salience more often preceded the following morning’s online news issue salience across overnight transitions. Most first-level cross-lagged correlations exceeded their Rozelle–Campbell (RCb) baselines, while second-level coefficients did so less consistently. These findings indicate that agenda leadership in a hybrid media system is temporally contingent and level-specific rather than static. The observed patterns represent temporally ordered associations and should not be interpreted as evidence of direct causal influence.

## Introduction

Power relations among media platforms have been recognized as the most important analytical focus of a hybrid media system in which digitalization has deeply transformed communication and news cycles [1]. One of the most prolific paradigms in media effects scholarship, agenda-setting theory [2] has changed over time by reframing the questions that serve as foundation for this emphasis. First-level agenda-setting theory posits that mass media tell the public what to think about [3]; second-level agenda-setting theory argues that they also help tell the public which attributes to think about regarding these issues [4]. This theory today has reached a new stage which questions how the media agenda itself is created. The intermedia agenda-setting (IMAS) literature addresses this question by examining how the agendas of different news sources and platforms are temporally related [5].

The power hierarchy traditionally held by elite news organizations [6] has been disrupted, particularly with the emergence of social media platforms (such as X [formerly Twitter]). Early IMAS research primarily examined relationships from elite news media toward other agendas, whereas reverse agenda-setting research considers whether social media agendas precede or are associated with subsequent mainstream media agendas [7,8]. That new media ecology, in turn, has propelled researchers further into an apparently more complicated quest for meaning organized by time. At this point, theories of kairologic time and right-time can elucidate the ways in which algorithms mine content out of a non-linear (chronos) temporality so that it arrives at the precise and most affectively resonant moment re-calibrating the circulation and timing apparatuses underpinning news [9]. This immediacy then forces news organizations to follow suit with that frenzied tempo, putting intraday agenda cycles at the center of political communication [10].

In this respect, the contextual importance of this study is to assess these power relations in or through the 31 March 2024 Istanbul local elections and a set of core components of the Türkiye media system. These elections were seen as a crucial opportunity for both the ruling and opposition parties to entrench themselves more deeply in the national political landscape. Istanbul, the largest electorate in Türkiye not only retains its symbolic significance to national politics but also remains a financial centre. In this regard, the intense interplay described here takes place in the context of Türkiye's polarized pluralist media system [11], featuring extreme political polarization, highly concentrated media ownership and strong political parallelism. This context provides a case for examining the temporal agenda relationships between X—one of the principal spaces for public expression today—and mainstream online news organizations.

However, despite global interest in IMAS, substantial gaps remain in the Türkiye context and in longitudinal research designs. Empirical IMAS studies in Türkiye remain scarce [12,13]. Existing studies have generally focused either on first-level agenda-setting processes [14,15] or on second-level processes [16], while few have examined both levels within an integrated design. Finally, comparing observed cross-lagged associations with the Rozelle–Campbell baseline (RCb) can strengthen the assessment of whether temporal ordering exceeds contemporaneous synchronization [17].

To fill these gaps, we utilize a comprehensive dataset covering the 31-day period leading up to and including the 31 March 2024 Istanbul local elections: specifically, 448,664 posts published on X and 10,357 articles by 19 mainstream online newspapers—together representing approximately sixty per cent of daily news traffic in the country. This study contributes to IMAS theory by treating agenda leadership as temporally contingent rather than as a stable property of either online news or X. It further distinguishes issue-level synchronization from attribute-level differentiation by examining both levels within the same temporal design. Empirically, these theoretical propositions are assessed across 12-hour intraday and day-to-day cycles using 122 cross-lagged panel analyses in a polarized pluralist media system.

## Theoretical background

### Stages of agenda-setting theory

Agenda-setting theory, arguably one of the oldest bodies of research in communication science (with over 50 years of literature), posits that though media don't do a great job telling people what to think, they do an excellent job dictating “what to think about” [2]. This flavor of core effect has been framed as first-level agenda-setting, in which salience assigned by the media to some issues is said to transfer itself to the public agenda. Second-level agenda-setting extends the concept of salience from issues or objects to the attributes used to characterize them [3,4]. The literature commonly distinguishes substantive (or cognitive) attributes, which describe characteristics of an object, from affective attributes, which convey evaluative valence [4]. In the present study, second-level IMAS refers to cross-platform temporal correspondence in the relative salience of these predefined attributes. This operationalization is broader than sentiment analysis, which concerns affective valence alone, but narrower than a full framing analysis, which examines broader organizing principles and interpretive packages [18]. Attribute emphasis may contribute to framing, but the present design does not identify or test the transfer of complete frames.

First- and second-level agenda transfer, however, need not operate with the same strength. Issue salience may converge because platforms respond to the same campaign events, institutional actors, and electoral timetable, whereas attribute salience requires greater similarity in evaluative tone and interpretive emphasis. Such attributes are more dependent on platform-specific production logics, editorial routines, audience expectations, and ideological positioning. In a polarized pluralist media system, X and online news may therefore converge on which issues receive attention while retaining greater autonomy in how those issues are characterized [1,11,18]. This provides a theoretical basis for the possibility that attribute agendas may be less synchronized and more resistant to cross-platform transfer than issue agendas.

IMAS represents a later development in agenda-setting theory because it shifts attention from relationships between media and public agendas to relationships among media agendas themselves [19]. Media agenda formation differs across traditional and digital environments. In traditional media systems, agenda relationships have largely followed a top-down structure dominated by established news organizations. Digital media, particularly social platforms, have complicated this hierarchy by allowing political actors and citizens to participate more directly in agenda construction [7,20].

X has become an important arena for political communication, particularly during election campaigns. Its rapid circulation of information, direct actor-to-public communication, and networked interaction make it relevant both as a news source and as an indicator of public discussion [8]. These features can make X salient to newsrooms, but they do not establish a direct causal effect on news agendas or public opinion. Accordingly, intermedia relationships between mainstream news and social media are better understood as reciprocal and context-dependent rather than unidirectional [21]. Such relationships may intensify during election campaigns, when political actors, journalists, and citizens produce and circulate information at an accelerated pace.

### IMAS digital temporality and IMAS in the electoral context

In order to understand the forces that put agendas into motion in the hybrid media system [1], we need not only to grasp relationships among actors but also to consider the temporal architecture of these platforms. Yet, within the continuous 24-hour news cycle, content is organized not only chronologically but also according to what is algorithmically treated as the right-time [9]. Political attention may therefore become concentrated around particular events and moments.

Traditional, online and social media have their own internal rhythms through which political events may generate synchronization across platforms [22]. Beyond the immediacy of digital media and the associated demand for continuous news production [23], professional and editorial routines also structure newsroom activity. Consequently, IMAS concerns not only which medium precedes another but also how temporal sequences interact with platform and newsroom routines.

Election campaigns are periods of intensified media activity and heightened public demand for information. Research in the United States has shown that agenda relationships among political blogs, Twitter, and mainstream news can be reciprocal and vary by issue and time lag [7,8]. Related work has found similarly intertwined agenda relationships between fake-news sites and online partisan media during the 2014–2016 period [21]. Evidence from the 2014 Belgian election campaign also indicates that established media remained important agenda setters, although influence varied across media types and publication rhythms [5]. Together, these studies suggest that IMAS dynamics are contingent on political context, media structure, platform type, and campaign timing.

From this perspective, Türkiye provides a relevant context for examining IMAS through concepts associated with the polarized pluralist model [11]. Concerns about media autonomy and independence have been widely documented [24,25], while studies of recent elections show that X/Twitter has become an important arena of political communication [15,26]. Research on the 2019 and 2024 Istanbul mayoral elections has examined candidates’ social-media content, self-presentation, and political storytelling [14,16,27,28]. Türkiye-based IMAS research has also investigated agenda relationships between online news and Twitter [29]. Within this literature, however, direct comparisons of issue- and attribute-level agenda relationships across intraday and interday temporal windows remain limited. The present study addresses this specific gap.

### Research questions and hypotheses

Building on this theoretical background, this study examines whether IMAS relationships between X and mainstream online news differ across levels of salience, cross-platform directions, and temporal transitions. It considers both first-level issue salience and second-level attribute salience across intraday (AM → PM) and day-to-day (PM→next-day AM) transitions.

**RQ1 (Cross-Platform IMAS Relationship):** During the 31 March 2024 Istanbul local election campaign, is there a statistically significant IMAS relationship between the X platform and mainstream online news media?

**H1.** During the 2024 Istanbul local election period, the agendas of X and mainstream online news media will be positively and significantly related.

**RQ2 (Difference Between the First and Second Levels):** Are agenda-setting relationships observed at both the first level and the second level? Which level exhibits stronger cross-platform associations?

**H2a.** IMAS relationships will be observed at both the first and second levels.

**H2b.** Second-level agenda-setting relationships will be stronger than first-level relationships.

**RQ3 (The Direction of Temporal Ordering and Asymmetry):** Which cross-platform direction exhibits the stronger temporally ordered association: earlier X→later online news or earlier online news→later X? How does this comparison vary across 12-hour temporal transitions (AM → PM and PM→next-day AM)?

**RQ3a.** Does the larger cross-lagged coefficient (earlier X→later online news vs. earlier online news→later X) systematically vary depending on the temporal transition (AM → PM vs. PM→next-day AM)?

**RQ3b.** Within day-to-day (PM→next-day AM) transitions, is the previous evening’s X agenda more strongly associated with the following morning’s online news agenda, or is the previous evening’s online news agenda more strongly associated with the following morning’s X agenda?

## Data and methodology

### Data collection and sampling

This study analyzes data collected from the X platform and mainstream online news websites during a 31-day period (1 March 2024–31 March 2024) surrounding the 31 March 2024 Istanbul local elections. The data collection process standardized all data according to Türkiye Standard Time (GMT + 3), thereby preventing any time-zone inconsistencies between the two platforms.

X data were obtained through Teleskop, a commercial social media monitoring provider with official API access, and the Boolean queries were implemented in collaboration with the provider. In the initial stage, a broad Boolean query related to the Istanbul local elections was used, yielding 512,438 posts. However, because not all of the retrieved content was election-related, a two-stage filtering procedure was implemented. First, automated filtering was applied using negative keywords; subsequently, 1,000 randomly selected posts were manually reviewed. This validation test achieved an accuracy rate of 94 percent. The 94 percent estimate refers exclusively to the relevance-screening procedure and not to the predictive performance of the subsequent issue or attribute classifiers. A total of 63,774 off-topic, spam-related, or otherwise irrelevant posts were excluded from the analysis, resulting in a final X dataset of 448,664 posts (see Appendix S1 in S1 File).

Online news sampling began with the 20 most visited news websites identified using Similarweb total-visits data for October–December 2023, together representing approximately 60 percent of Türkiye’s daily online news traffic. Because the available content stream for one outlet was incomplete during the study period, that outlet was excluded, and the final sample comprised 19 websites. News articles were collected via a Python-based web scraper, with titles, content, and timestamps (GMT + 3) recorded in a standardized format, resulting in a dataset of 10,357 news texts. All news texts were preprocessed through stop-word removal and tokenization, yielding a coherent textual structure suitable for analysis (see Appendix S2 in S1 File).

### Ethical considerations and user privacy

Only publicly accessible X posts were included in the study; no private or protected-account content or direct messages were accessed. Data were collected through a commercial social media monitoring provider with official API access and were processed in accordance with the platform terms applicable during the data-collection period. The collection and analysis of X data complied with the applicable X terms and conditions and with the data-access conditions of the third-party provider in effect at the time of data collection. The analysis focused on aggregate agenda patterns rather than individual users. Usernames, user IDs, profile information, post IDs, post URLs, and verbatim post texts from the collected records were excluded from the publicly shared analysis files, and no identifiable X post is quoted or reproduced in the manuscript. The documented query strings in the Supporting Information include only the public names and account handles used as search terms. The publicly shared files contain only processed numerical matrices and synthetic, non-semantic indicator identifiers that cannot be linked to individual accounts.

### Operationalization and content analysis

For first-level agenda measurement, 5 percent of the dataset was manually reviewed by two independent coders, and the S3 codebook was developed to comprise 11 substantive issue categories, their subcategories, and the associated matched indicators reflecting the Istanbul election process (see Appendix S3 in S1 File). Intercoder reliability was assessed on a randomly selected sample of 1,000 content items and yielded Krippendorff’s α = .86. This coefficient reflects agreement between the human coders and should not be interpreted as the predictive performance of an automated classifier.

For second-level agenda measurement, the S4 codebook comprised eight attribute categories (see Appendix S4 in S1 File). Positive (Emotional/Encouraging) and Negative (Emotional/Reactive) were treated as affective attributes, whereas Public Connection/Empathy, Decisiveness/Crisis Management, Vision/Innovation, Experience/Competence, Leadership/Charisma, and Ideology/Partisanship were treated as substantive or cognitive attributes. Intercoder reliability for the second-level codebook was assessed on a randomly selected sample of 1,000 content items and yielded Krippendorff’s α = .82. This coefficient likewise represents agreement between the human coders rather than classifier accuracy.

The operational procedure used to generate the inferential analysis matrices was dictionary-supervised and indicator-based rather than a document-level machine-learning classifier. The matched indicators in the S3 and S4 codebooks were applied to both X posts and online news texts. Repeated occurrences of the same indicator within a content item were counted once, whereas distinct matched indicators could each contribute to the corresponding temporal frequency profile. Consequently, the main analytical procedure did not force every document into a single issue or attribute category. Content without a matched S3 or S4 indicator did not contribute an indicator count.

For each corpus and each 12-hour window, the frequency values of the ordered matched indicators were assembled into temporal analysis vectors. The first-level matrix contained 375 ordered issue indicators, and the second-level matrix contained 122 ordered attribute indicators. Cross-lagged Pearson correlations were calculated across the corresponding indicator-frequency vectors for X and online news. The 11 issue categories and eight attribute categories therefore provide the conceptual grouping of the indicators, whereas the inputs to the cross-lagged and RCb analyses were the 375- and 122-indicator frequency matrices—not vectors consisting of only 11 or eight aggregate category percentages. These matrices and the reproduction code are provided in the OSF repository described in the Data Availability Statement.

As a separate auxiliary benchmark conducted during the revision, two pretrained Turkish transformers—dbmdz/bert-base-turkish-128k-cased (BERTurk) and YSKartal/berturk-social-5m—were compared on adjudicated human-coded X texts for document-level issue classification. BERTurk was selected based on macro-F1 on the internal development set. The benchmark used a single-label, multiclass architecture with the 11 substantive issue categories and a twelfth “None/Outside codebook” class. On a separate locked test set of 200 X posts, it achieved an accuracy of .735, balanced accuracy of .697, macro-F1 of .630, and weighted F1 of .745. This auxiliary benchmark was not used to generate the 375-indicator issue matrix or the cross-lagged and RCb results reported in the study and should not be interpreted as the validation accuracy of the main matched-indicator procedure. Its complete 12 × 12 confusion matrix and class-wise precision, recall, F1, and support values are provided in the workbook X_Nihai_Test_Raporu_v2_1.xlsx within the OSF archive 02_Classifier_Validation/X_issue_classifier_validation_issue_only_v1.zip.

### Analytical strategy: The 12-hour rhythm and cross-lagged analysis

In this study, agenda relationships between media platforms are examined across fixed 12-hour cycles. Prior research shows that online intermedia agenda-setting can unfold within a single day [10], while the accelerating temporality of platforms [9] and the rhythmic organization of election-period information flows [22] make intraday segmentation theoretically relevant. We selected two 12-hour intervals as a balance between temporal sensitivity and measurement stability: shorter intervals risk sparse or zero counts, particularly for less frequent issue and attribute indicators in the news corpus, whereas 24-hour aggregation would obscure the intraday transitions central to RQ3. The labels AM and PM are operational clock-time designations rather than claims that all hours within each interval represent homogeneous media or audience routines. AM (00:01–12:00) captures overnight carryover together with early-day news production, whereas PM (12:01–00:00) captures the accumulation of afternoon and evening activity. The 31-day period therefore yielded 62 unique 12-hour time blocks. These blocks formed 31 same-day AM → PM transitions and 30 between-day PM→next-day AM transitions. Because each transition was analyzed separately for the first- and second-level agendas, the design produced 62 intraday cross-lagged panels (31 days × 2 levels) and 60 interday cross-lagged panels (30 day-pairs × 2 levels), for a total of 122 panels.

The analysis relies on cross-lagged panel correlations. For each temporal transition, both cross-platform directions were estimated and compared: X in the earlier interval with online news in the later interval, and online news in the earlier interval with X in the later interval. The AM → PM panels represent same-day transitions, whereas the PM→next-day AM panels represent overnight transitions to the following morning. These coefficients are interpreted as temporally ordered associations rather than direct causal effects.

Cross-lagged coefficients can be elevated by contemporaneous cross-platform synchronization and within-platform temporal stability. To assess whether the observed coefficients exceeded a baseline incorporating these components, the study uses the Rozelle–Campbell baseline (RCb) [30]. The RCb compares each observed cross-lagged correlation with a baseline derived from the contemporaneous cross-platform correlations and the within-platform autocorrelations. When an observed cross-lagged coefficient exceeds the RCb value, it is interpreted here as a temporally ordered association that is stronger than expected from synchronous covariation and temporal stability alone; this comparison does not establish direct causation. The procedure was applied to all 122 cross-lagged panels: 62 intraday panels (31 days × 2 agenda-setting levels) and 60 interday panels (30 day-pairs × 2 levels), providing an additional check against contemporaneous synchronization.


$$\begin{array}{c} \text{R}{\text{C}}_{\text{b}}=\left(\frac{{\text{P}}_{\text{X1Y1}}+{\text{P}}_{\text{X2Y2}}}{\text{2}}\right)*{\left(\frac{{\left({\text{P}}_{\text{X1X2}}\right)}^{\text{2}}+{\left({\text{P}}_{\text{Y1Y2}}\right)}^{\text{2}}}{\text{2}}\right)}^{\frac{\text{1}}{\text{2}}} \end{array}$$

Here, X1Y1 and X2Y2 represent the contemporaneous cross-platform correlations, whereas X1X2 and Y1Y2 represent the within-platform autocorrelations, or temporal stability, between the earlier and later intervals. By incorporating contemporaneous cross-platform correspondence and within-platform temporal stability, the formula provides a baseline against which the observed cross-lagged coefficients can be compared. It does not constitute a formal test of direct causation or eliminate the possibility of unmeasured common drivers.

## Findings

### First-Level IMAS: Issue salience

In the first stage of the analysis, the issue agendas of X and online news media during the 31-day campaign period were examined. The three most prominent issue categories on both platforms were, in order, “Party/Alliance,” “Candidate/Actor,” and “Election/Campaign.” This pattern is consistent with the expectation that media attention during election periods concentrates on the principal actors and processes of politics. Nevertheless, a notable difference in concentration is observed between the two platforms. These three categories accounted for approximately 74% of the matched-indicator frequency on X and 67% in online news. This indicates that, compared with the news media, the X platform exhibits a narrower and more concentrated political agenda. In contrast, several policy- and issue-oriented categories—notably “Transportation/Infrastructure,” “Environment/Projects,” and “Security/Law”—accounted for larger shares in online news than on X (Fig 1).

**Fig 1:**
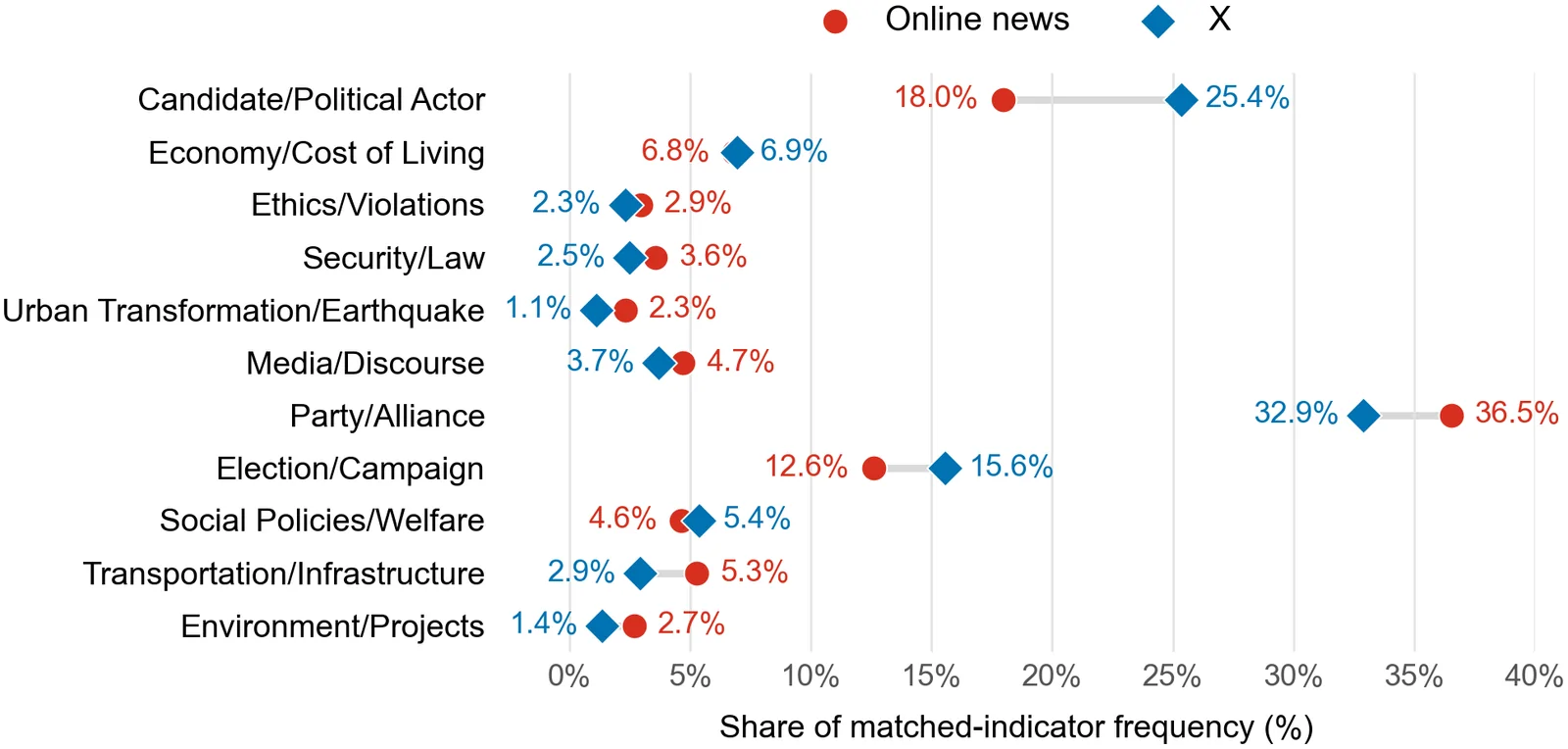
Percentage Distribution of First-Level Issue Agendas.

Following this overall picture, cross-lagged correlations were examined to assess the temporally ordered relationships between the two agendas. First-level associations were consistently strong: across the 122 directional coefficients, values ranged from .874 to .971, and 111 coefficients (91.0%) exceeded .90. All coefficients were positive and statistically significant (p < .001), supporting H1. Detailed intraday and day-to-day correlation matrices are provided in Appendix S5 in S1 File. The intraday and day-to-day first-level results are presented in Tables 1 and 2, respectively.

**Table 1:** Intraday relationships for first-level agenda-setting (AM–PM).

	Days	X1Y2	Y1X2	RCb
**March 1**	Day 1	0.883	0.898	0.885
	Day 2	0.886	0.927	0.897
	Day 3	0.916	0.932	0.916
	Day 4	0.902	0.894	0.892
	Day 5	0.902	0.898	0.881
	Day 6	0.910	0.913	0.891
	Day 7	0.901	0.940	0.903
	Day 8	0.936	0.954	0.924
	Day 9	0.922	0.910	0.893
	Day 10	0.931	0.933	0.919
	Day 11	0.899	0.950	0.910
	Day 12	0.933	0.944	0.931
	Day 13	0.917	0.947	0.910
	Day 14	0.943	0.942	0.929
	Day 15	0.944	0.955	0.942
	Day 16	0.948	0.965	0.946
	Day 17	0.937	0.950	0.926
	Day 18	0.938	0.934	0.919
	Day 19	0.930	0.936	0.914
	Day 20	0.948	0.947	0.940
	Day 21	0.955	0.959	0.952
	Day 22	0.943	0.943	0.924
	Day 23	0.919	0.945	0.920
	Day 24	0.944	0.942	0.936
	Day 25	0.931	0.945	0.929
	Day 26	0.957	0.938	0.939
	Day 27	0.951	0.950	0.942
	Day 28	0.952	0.968	0.952
	Day 29	0.963	0.966	0.959
	Day 30	0.938	0.947	0.936
**March 31**	Day 31	0.926	0.933	0.911

X1Y2 is the correlation between the morning (AM) X agenda and the subsequent afternoon/evening (PM) online news agenda; Y1X2 is the correlation between the morning (AM) online news agenda and the subsequent afternoon/evening (PM) X agenda. These coefficients indicate temporal ordering, not direct causal effects. Directional-dominance and RCb-threshold comparisons reported in the text were based on unrounded values; table entries are displayed to three decimal places. Red-shaded cells indicate cross-lagged coefficients below the corresponding unrounded RCb value.

**Table 2:** Day-to-day relationships for first-level agenda-setting (PM–Next-Day AM).

	Days	X2Y3	Y2X3	RCb
**March 1-2**	Days 1 and 2	0.909	0.880	0.896
	Days 2 and 3	0.921	0.911	0.918
	Days 3 and 4	0.940	0.899	0.887
	Days 4 and 5	0.874	0.905	0.885
	Days 5 and 6	0.910	0.908	0.884
	Days 6 and 7	0.920	0.886	0.910
	Days 7 and 8	0.943	0.954	0.924
	Days 8 and 9	0.937	0.898	0.895
	Days 9 and 10	0.935	0.929	0.924
	Days 10 and 11	0.947	0.906	0.920
	Days 11 and 12	0.957	0.926	0.927
	Days 12 and 13	0.940	0.917	0.916
	Days 13 and 14	0.955	0.929	0.925
	Days 14 and 15	0.951	0.953	0.943
	Days 15 and 16	0.965	0.950	0.946
	Days 16 and 17	0.964	0.941	0.934
	Days 17 and 18	0.932	0.946	0.915
	Days 18 and 19	0.925	0.917	0.920
	Days 19 and 20	0.961	0.927	0.934
	Days 20 and 21	0.947	0.952	0.950
	Days 21 and 22	0.958	0.961	0.930
	Days 22 and 23	0.933	0.902	0.923
	Days 23 and 24	0.954	0.946	0.945
	Days 24 and 25	0.935	0.944	0.927
	Days 25 and 26	0.941	0.938	0.938
	Days 26 and 27	0.947	0.953	0.943
	Days 27 and 28	0.965	0.951	0.956
	Days 28 and 29	0.971	0.952	0.955
	Days 29 and 30	0.947	0.955	0.941
**March 30-31**	Days 30 and 31	0.927	0.928	0.909

X2Y3 is the correlation between one day’s afternoon/evening (PM) X agenda and the following day’s morning (AM) online news agenda; Y2X3 is the correlation between one day’s afternoon/evening (PM) online news agenda and the following day’s morning (AM) X agenda. These coefficients indicate temporal ordering, not direct causal effects. Directional-dominance and RCb-threshold comparisons reported in the text were based on unrounded values; table entries are displayed to three decimal places. Red-shaded cells indicate cross-lagged coefficients below the corresponding unrounded RCb value.

However, comparison of the cross-lagged coefficients underlying this high level of synchronization (RQ3a and RQ3b) presents a more differentiated pattern. In the intraday (AM → PM) analyses, the earlier-news→later-X coefficient was larger on 21 of the 31 days, whereas the earlier-X→later-news coefficient was larger on 10 days. Thus, morning news salience more often preceded afternoon/evening X salience than the reverse within same-day transitions. This temporal ordering does not establish that changes in the news agenda caused the subsequent X agenda.

In contrast, the day-to-day (PM→next-day AM) analyses present a different pattern. On 20 of the 30 day-pairs, the coefficient relating the evening X agenda to the following morning’s online news agenda was larger than the reverse cross-lagged coefficient; the earlier-news→later-X coefficient was larger on 10 day-pairs. This pattern suggests that issues salient on X at the end of the day more often preceded similar issue salience in the following morning’s online news. Considered together with the intraday findings, the results indicate that temporal ordering varies across different segments of the day, but they do not identify direct cross-platform causation or exclude shared external drivers.

### Second-Level IMAS: Attribute salience

The descriptive second-level comparison summarizes the relative prominence of the eight predefined S4 attribute categories used to group the 122 operational indicators. It therefore examines attribute emphasis rather than sentiment alone or complete interpretive frames. On X, the two most prominent categories were Public Connection/Empathy, a substantive attribute, and Negative (Emotional/Reactive), an affective attribute. In online news, the distribution was more balanced, with Positive (Emotional/Encouraging) and Decisiveness/Crisis Management also occupying a prominent position (Fig 2). These distributions describe the substantive and affective emphases of the two platforms; H2a is evaluated through the cross-lagged analyses reported below.

**Fig 2:**
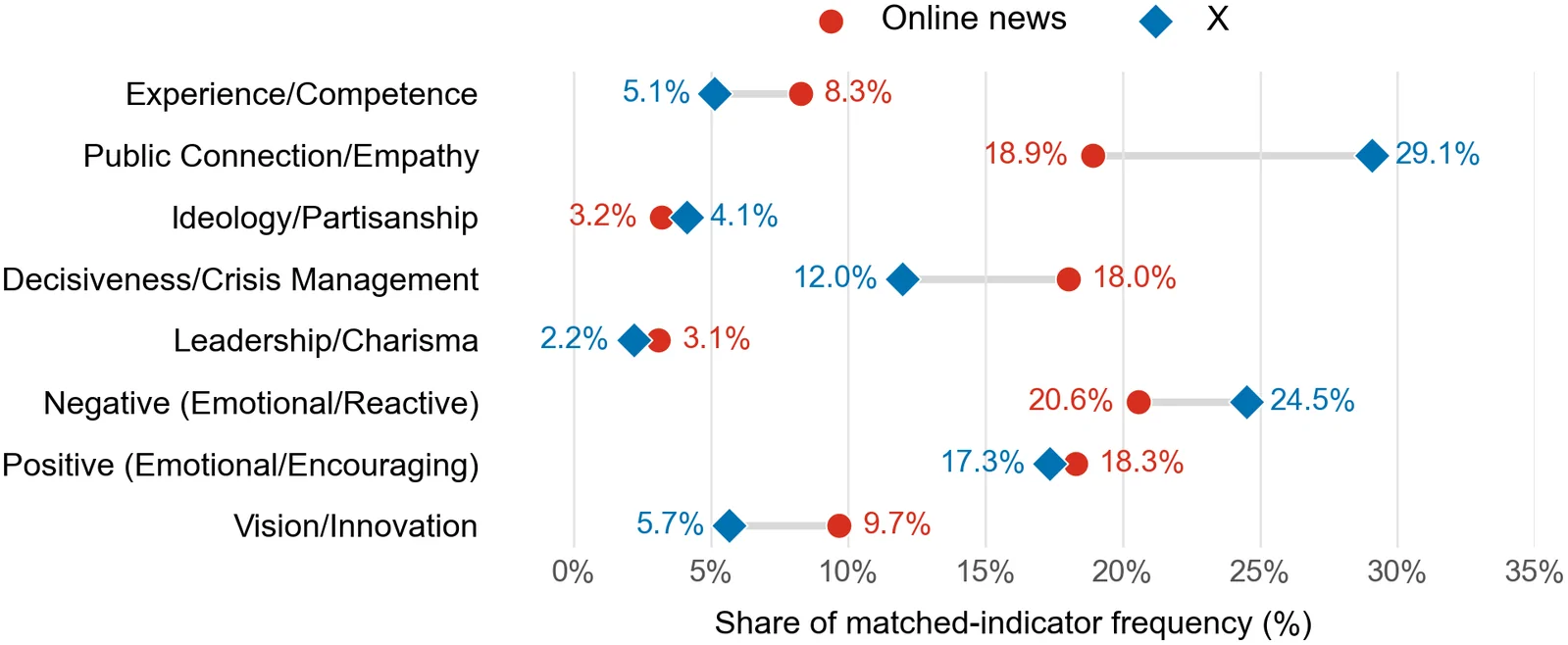
Percentage distribution of second-level attribute agendas.

Second-level agenda relationships were weaker than first-level relationships. Across the 122 directional second-level coefficients, values ranged from r = .293 to .853, and all were positive and statistically significant (all p < .01), supporting H2a. These coefficients were lower than the first-level coefficients (r = .874–.971), thereby rejecting H2b and indicating that first-level agenda relationships were stronger than second-level relationships. Full cross-lagged correlation tables for the second-level attribute agendas across all temporal windows are provided in Appendix S6 in S1 File. The corresponding intraday and day-to-day second-level results are presented in Tables 3 and 4, respectively.

**Table 3:** Intraday relationships for second-level agenda-setting (AM–PM).

	Days	X1Y2	Y1X2	RCb
**March 1**	Day 1	0.441	0.502	0.463
	Day 2	0.407	0.583	0.450
	Day 3	0.482	0.424	0.421
	Day 4	0.503	0.435	0.433
	Day 5	0.333	0.502	0.409
	Day 6	0.491	0.549	0.482
	Day 7	0.444	0.591	0.484
	Day 8	0.585	0.684	0.579
	Day 9	0.416	0.459	0.401
	Day 10	0.554	0.650	0.543
	Day 11	0.724	0.621	0.592
	Day 12	0.745	0.642	0.667
	Day 13	0.576	0.668	0.563
	Day 14	0.735	0.818	0.726
	Day 15	0.801	0.672	0.700
	Day 16	0.637	0.562	0.574
	Day 17	0.617	0.793	0.659
	Day 18	0.702	0.684	0.640
	Day 19	0.656	0.656	0.563
	Day 20	0.671	0.690	0.624
	Day 21	0.655	0.782	0.701
	Day 22	0.654	0.615	0.549
	Day 23	0.473	0.462	0.424
	Day 24	0.630	0.492	0.514
	Day 25	0.646	0.663	0.606
	Day 26	0.740	0.783	0.727
	Day 27	0.746	0.745	0.721
	Day 28	0.702	0.673	0.669
	Day 29	0.739	0.744	0.711
	Day 30	0.598	0.700	0.612
**March 31**	Day 31	0.599	0.554	0.543

X1Y2 is the correlation between the morning (AM) X agenda and the subsequent afternoon/evening (PM) online news agenda; Y1X2 is the correlation between the morning (AM) online news agenda and the subsequent afternoon/evening (PM) X agenda. These coefficients indicate temporal ordering, not direct causal effects. Directional-dominance and RCb-threshold comparisons reported in the text were based on unrounded values; table entries are displayed to three decimal places. Red-shaded cells indicate cross-lagged coefficients below the corresponding unrounded RCb value.

**Table 4:** Day-to-day relationships for second-level agenda-setting (PM–Next-Day AM).

	Days	X2Y3	Y2X3	RCb
**March 1–2**	Days 1 and 2	0.598	0.377	0.466
	Days 2 and 3	0.350	0.467	0.424
	Days 3 and 4	0.555	0.414	0.437
	Days 4 and 5	0.406	0.510	0.399
	Days 5 and 6	0.540	0.435	0.490
	Days 6 and 7	0.548	0.386	0.490
	Days 7 and 8	0.620	0.702	0.624
	Days 8 and 9	0.690	0.293	0.406
	Days 9 and 10	0.474	0.553	0.549
	Days 10 and 11	0.754	0.668	0.629
	Days 11 and 12	0.620	0.674	0.635
	Days 12 and 13	0.650	0.690	0.574
	Days 13 and 14	0.801	0.597	0.700
	Days 14 and 15	0.730	0.798	0.715
	Days 15 and 16	0.639	0.713	0.587
	Days 16 and 17	0.661	0.666	0.680
	Days 17 and 18	0.749	0.628	0.651
	Days 18 and 19	0.589	0.677	0.595
	Days 19 and 20	0.684	0.647	0.611
	Days 20 and 21	0.760	0.738	0.712
	Days 21 and 22	0.764	0.635	0.583
	Days 22 and 23	0.545	0.457	0.450
	Days 23 and 24	0.487	0.622	0.531
	Days 24 and 25	0.515	0.568	0.604
	Days 25 and 26	0.708	0.756	0.728
	Days 26 and 27	0.720	0.771	0.715
	Days 27 and 28	0.767	0.754	0.694
	Days 28 and 29	0.666	0.682	0.706
	Days 29 and 30	0.853	0.728	0.659
**March 30–31**	Days 30 and 31	0.492	0.656	0.492

X2Y3 is the correlation between one day’s afternoon/evening (PM) X agenda and the following day’s morning (AM) online news agenda; Y2X3 is the correlation between one day’s afternoon/evening (PM) online news agenda and the following day’s morning (AM) X agenda. These coefficients indicate temporal ordering, not direct causal effects. Directional-dominance and RCb-threshold comparisons reported in the text were based on unrounded values; table entries are displayed to three decimal places. Red-shaded cells indicate cross-lagged coefficients below the corresponding unrounded RCb value.

Second, the comparison of cross-lagged coefficients differs from that observed at the first level. In the intraday (AM → PM) analyses, the earlier-news→later-X coefficient was larger on 18 of the 31 days, whereas the earlier-X→later-news coefficient was larger on 13 days. In the day-to-day (PM→next-day AM) analyses, the earlier-news→later-X coefficient was larger on 16 of the 30 day-pairs, whereas the earlier-X→later-news coefficient was larger on 14 day-pairs. Thus, the clear predominance of the X-before-news coefficient observed in the first-level interday analysis was not reproduced at the second level; attribute-level temporal ordering was more balanced.

### RCb threshold results

RCb comparisons were conducted to assess whether the cross-lagged correlations exceeded the level expected from contemporaneous synchronization and within-platform temporal stability. At the first level, at least one cross-lagged coefficient fell below the RCb value on 7 of the 31 intraday panels and 13 of the 30 interday panels. Thus, most first-level cross-lagged associations exceeded their corresponding RCb baselines, although this comparison does not by itself establish causal influence. Comprehensive RCb results for all 122 cross-lagged panels are provided in Appendix S7 in S1 File.

At the second level, at least one cross-lagged coefficient fell below the RCb value on 11 of the 31 intraday panels and 18 of the 30 interday panels. Attribute-level associations therefore exceeded their RCb baselines less consistently than issue-level associations.

Together, these results reinforce the rejection of H2b and indicate that the issue agendas of the two platforms are more closely aligned than their attribute agendas. The findings concern differences in the strength and stability of temporally ordered associations and should not be interpreted as evidence of direct agenda transfer.

## Discussion

This study examined IMAS relationships over time in the context of Türkiye’s polarized pluralist media system. The results indicate significant temporally ordered associations between X and mainstream online news in both cross-platform directions. However, the relative magnitude of these associations differs across issue- versus attribute-level agendas and intraday versus interday transitions. These patterns are interpreted as cross-lagged associations rather than evidence that either platform directly caused changes in the other platform’s agenda.

The strong synchronization between the issue agendas is one of the study’s most notable findings. This pattern is consistent with theoretical accounts of a hybrid media system in which news organizations and social media actors continuously monitor, respond to, and compete with one another [1]. Possible explanations include journalists’ use of X as a news-gathering source and efforts to remain competitive in the news cycle, as well as political actors’ simultaneous dissemination of messages through traditional and social media.

X’s prominence in election communication may also be related to platform affordances that distinguish it from mainstream online news, including rapid publication, direct actor-to-public communication, real-time interaction, and algorithmically organized visibility [8,9]. These features can make campaign issues immediately visible and contestable, whereas online news remains more strongly organized by editorial routines and production schedules. This contrast may help explain why political issues can gain rapid visibility on X and why the platform remains relevant to journalists and political actors during election campaigns. These affordances are offered as contextual explanations and should not be interpreted as causes of the observed cross-lagged relationships.

Yet, in the context of Türkiye’s political polarization and concentrated media ownership [11], this high synchronization may reflect not only agenda convergence but also a possible form of “agenda compression.” The prominence of Party/Alliance and Candidate/Political Actor content in both media spaces may leave comparatively limited agenda space for alternative or less-supported policy issues. This interpretation is noteworthy because X might be expected to be less constrained by institutional news-media routines; nevertheless, the observed similarity suggests that both media environments may operate within overlapping structures of political attention that limit agenda diversity in a polarized media system.

Furthermore, comparison of the cross-lagged coefficients indicates that temporal ordering differs across segments of the day. At the first level, the earlier-news→later-X coefficient was more often larger within same-day AM → PM transitions, whereas the earlier-X→later-news coefficient was more often larger across PM→next-day AM transitions. This pattern is consistent with the different production cycles and functions of online news and X, but it cannot determine whether either platform directly caused subsequent changes in the other. One plausible interpretation is that morning news salience may provide material for later discussions on X, whereas issues salient on X in the evening may be available to the following morning’s news cycle; shared campaign events nevertheless remain an alternative explanation. These temporally contingent associations are compatible with research emphasizing the continuing role of traditional media [5], the growing role of social media [8], reciprocal IMAS relationships [21], and the kairologic logic of algorithmic media [9].

Two illustrative episodes help place these day-level fluctuations in their campaign context. First, on 24 March, President Erdoğan and Istanbul mayoral candidate Murat Kurum participated in the “Yeniden Büyük İstanbul Mitingi” at Atatürk Airport, an event that began during the PM interval [31]. On that day, the first-level intraday coefficients were nearly symmetrical (earlier X→later news, r = .944; earlier news→later X, r = .942), which could reflect the common salience of the same major campaign event across both platforms. At the attribute level, however, the larger coefficient varied by temporal transition: earlier news→later X was larger across the 23 March PM → 24 March AM transition (.622 vs. .487), whereas earlier X→later news was larger within the 24 March AM → PM transition (.630 vs. .492). Second, the final campaign weekend produced a sharper attribute-level shift. The earlier X→later news coefficient reached .853 across the 29 March PM → 30 March AM transition, compared with .728 in the reverse direction. Across the 30 March PM → 31 March AM transition—which straddled the legally mandated 18:00 end of campaign propaganda and the move into election-day conditions [32]—the larger coefficient reversed (earlier news→later X, r = .656; earlier X→later news, r = .492). These examples are post hoc contextual illustrations and do not demonstrate that the events caused the observed correlations; rather, they show that temporal ordering was embedded in a campaign schedule containing major rallies, formal campaign closure, and the transition to election day.

Across the reported panels, first-level agenda relationships were generally stronger than second-level relationships. While the platforms converged closely on which issues received attention, they diverged more in the attributes made salient when characterizing candidates, issues, and political performance. The greater prominence of Public Connection/Empathy and Negative attributes on X, compared with the more balanced and performance-oriented attribute distribution in online news, is consistent with platform-specific patterns of substantive and affective emphasis [18]. Second-level cross-lagged coefficients also exceeded the RCb threshold less consistently, suggesting that the 122-indicator attribute-frequency profiles were less closely aligned beyond contemporaneous synchronization. The present study does not identify complete frames or demonstrate frame transfer; it shows that cross-platform alignment was weaker for the 122-indicator attribute-frequency profiles than for the 375-indicator issue-frequency profiles.

Taken together, these findings refine IMAS theory by moving beyond the static question of which medium leads. Agenda leadership appears temporally contingent: the platform that precedes the other differs across intraday and day-to-day cycles. Cross-platform convergence is also level-specific, because strong synchronization in issue salience does not entail equivalent convergence in attribute salience. The Türkiye case further suggests that a polarized hybrid media system can concentrate attention around shared issues while preserving platform-specific evaluative and interpretive patterns. IMAS is therefore better understood as a temporally and analytically layered relationship whose direction and strength vary across both time windows and levels of salience.

### Limitations and future research

The first limitation concerns the correlational design of the study. Cross-lagged correlations and the RCb comparison establish temporal ordering and assess whether coefficients exceed a contemporaneous baseline, but they cannot identify direct causal effects. Unmeasured shared drivers—including campaign events, candidate communications, and broader news cycles—may account for part of the observed associations. Accordingly, the terms “agenda leadership” and “agenda-setting” are used as conventional descriptors within the IMAS literature and not as claims of experimentally identified causation.

Second, this research is limited in regard to time and content; it only concerns the 2024 Istanbul local elections. Although Istanbul is a major political and media center in Türkiye, caution is warranted when generalizing the dynamics observed in this city to other Turkish cities or to national elections. The fixed AM/PM specification does not resolve variation within either 12-hour block. Accordingly, the findings are interpreted only for the selected aggregation and should not be assumed to generalize to shorter or differently shifted temporal windows; future research should test such alternatives using hour-level issue- and attribute-salience series.

Third, the hybrid media system encompasses additional platforms, including the image-oriented platform Instagram, the video-sharing platform TikTok, and the closed-group messaging service WhatsApp. Future studies could incorporate these platforms to develop a more comprehensive IMAS model.

Lastly, the analysis addressed only the first and second levels of agenda-setting. Future research could extend this contribution by examining cross-platform correspondence not only in issues and attributes but also in the networks of meaning formed by relationships among these elements (third-level network agenda-setting).

## Supporting information

S1 File Supporting information on data collection, coding, classifier validation, and statistical analyses. Contains the keyword query system and data collection procedures (Appendix S1), online news outlet sampling frame (Appendix S2), first-level issue codebook (Appendix S3), auxiliary transformer benchmark (Appendix S3A), second-level attribute codebook (Appendix S4), first-level correlation results (Appendix S5), second-level correlation results (Appendix S6), and RCb results (Appendix S7).
(DOCX)
