# Supplementary material for: Whose morning, whose evening? Temporal asymmetries in intermedia agenda-setting between X and online news in Türkiye
Source: PLoS One. 2026 Nov 6;21(11):e0362255. doi: 10.1371/journal.pone.0362255 (PMC13742923; doi:10.1371/journal.pone.0362255)
Supplement: Supplementary file 1 [file pone.0362255.s001.docx]

**S1 File. Supporting information**

**Whose Morning, Whose Evening? Temporal Asymmetries in Intermedia Agenda-Setting Between X and Online News in Türkiye**

**Appendix S1. Keyword query system and data collection procedures**

X data used in the time-series analyses were collected through the X API with support from Teleskop, a media monitoring company with official API access. Online news data were collected separately from publicly accessible news websites using a Python-based web scraper. In collaboration with Teleskop, all queries were implemented in bracketed Boolean format in the Q field; lang:tr was used as a language filter, retweets were excluded (-is:retweet), and replies and quote posts were treated separately in alternative query versions prepared for sentiment-related extensions. Unicode and diacritic variants (e.g., i/ı, ş/s, ö/o) were normalized, and mixed Turkish/English hashtag variants were incorporated into the query design.

The data collection period ran from 1 March 2024, 00:00 to 31 March 2024, 23:59. Negative keyword lists were updated in revisions dated 5 March and 12 March 2024 in order to reduce noise. For each version, hit rates were checked on a 5% random sample and the query lists were refined accordingly. News content was deduplicated using canonical URLs, and UTM and AMP parameters were removed.

Data collection was carried out in accordance with the X API Terms of Service and applicable compliance principles. News data were collected only from publicly accessible webpages. Queries were executed through two access profiles, after which the results were merged and duplicates were removed. The query structure was designed to capture the Istanbul context and election-related vocabulary while expanding candidate, party, district, and event signals through OR logic and reducing noise through basic negative filters.

**Q1.** ('Istanbul' OR 'İstanbul' OR 'İBB') (election OR 'local election' OR municipality OR mayor OR ballot box OR 'vote counting' OR poll OR rally) plus 39 district names

**Q2.** (('Ekrem İmamoğlu' OR 'Ekrem Imamoglu' OR İmamoğlu OR Imamoglu OR @ekrem_imamoglu) OR ('Murat Kurum' OR @murat_kurum)) (election OR 'local election' OR municipality OR mayor OR ballot box)

**Q3.** ((CHP OR 'Republican People's Party' OR @herkesicinCHP) OR ('AK Party' OR AKP OR 'Justice and Development Party' OR @Akparti)) (Istanbul OR IBB) (election OR 'local election')

**Q4.** ('Istanbul' OR 'İstanbul' OR 'İBB') (Kadıköy OR Üsküdar OR Ümraniye OR Şişli OR Beşiktaş ... ) (election OR 'local election' OR ballot box OR rally) / ('Istanbul' OR 'IMM') ('rally' OR 'TV program' OR 'election broadcast' OR 'minutes' OR 'objection')

**Note:** Istanbul context (core): ("Istanbul" OR "İstanbul" OR "IMM" OR "Metropolitan") + 39 district names / Election dictionary: (election OR "local election" OR municipality OR mayor OR ballot box OR "vote" OR "vote counting" OR "survey" OR rally) / Noise reduction: match - derby - air - discount - 'Ankara' - 'Izmir' – ‘Bursa’ – ‘Antalya’ ‘Konya’ – ‘Adana’ – ‘Şanlıurfa’ – ‘Gaziantep’ – ‘Kocaeli’

**Appendix S2. Online news outlet sampling frame**

The sample of mainstream online news outlets was determined using Similarweb total visits data for the October–December 2023 period. Of the 20 most visited news sites identified through traffic data, one outlet was excluded because its content stream was interrupted during the data collection period. The final sample therefore consisted of 19 sites (accessed 5 March 2024).

This selection was used because the 2023 traffic data reflect audience patterns that had stabilized prior to the election period. The resulting sample captures diversity across outlets with different editorial orientations and includes news organizations that play a significant role in shaping news visibility and agenda flows in Türkiye.

**S2 Table.** Ranking of Most Visited Mainstream Media Organizations

| **No** | **News media** | **Web traffic** |  | **No** | **News media** | **Web traffic** |
| --- | --- | --- | --- | --- | --- | --- |
| **1** | hürriyet.com.tr | 354.4 million (6.87%) |  | **11** | haberler.com | 123.7 million (2.38%) |
| **2** | milliyet.com.tr | 300.1 million (5.89%) |  | **12** | onedio.com | 120.8 million (2.35%) |
| **3** | mynet.com | 267.8 million (5.40%) |  | **13** | yenicaggazetesi.com.tr | 112.0 million (2.24%) |
| **4** | sozcu.com.tr | 251.2 million (4.81%) |  | **14** | takvim.com.tr | 87.0 million (1.72%) |
| **5** | sabah.com.tr | 187.3 million (3.63%) |  | **15** | internethaber.com | 72.3 million (1.41%) |
| **6** | haberturk.com | 179.3 million (3.45%) |  | **16** | cnnturk.com | 58.2 million (1.10%) |
| **7** | sondakika.com | 166.9 million (3.29%) |  | **17** | yeniakit.com.tr | 48.2 million (0.95%) |
| **8** | ensonhaber.com | 162.9 million (3.10%) |  | **18** | posta.com.tr | 47.9 million (0.95%) |
| **9** | cumhuriyet.com.tr | 135.3 million (2.49%) |  | **19** | birgun.net | 47.6 million (0.92%) |
| **10** | haber7.com | 129.3 million (2.51%) |  | **20** | trthaber.com | 45.9 million (0.90%) |

***Note.*** *birgun.net was included in the initial 20-outlet sampling frame but was excluded from the final analytic sample because its available content stream was incomplete during the study period. The final online news sample therefore comprised 19 outlets.*

**Appendix S3. First-Level Agenda (Issue)**

Operational note. The S3 categories provide the conceptual grouping of the matched issue indicators. For the main inferential analyses, the S3 indicators were applied to both X posts and online news texts using dictionary-supervised matching. Repeated occurrences of the same indicator within an item were counted once, whereas distinct matched indicators could each contribute to the temporal frequency profile. The resulting ordered matrix contained 375 issue indicators. Cross-lagged and RCb analyses were calculated from these indicator-frequency vectors rather than from document-level machine-learning predictions or vectors containing only 11 aggregate category percentages.

**S3 Table:** Issue Categories and Matched Keywords

| **Categories** | **Matched keywords (TR originals and EN glosses)** |
| --- | --- |
| **Candidate/Political Actor** | **TR→** kurum, imamoğlu, başkan, ibb, erdoğan, özel, aday, bakan, yavaş, bahçeli, kılıçdaroğlu, cumhurbaşkanı, erbakan, altınok, akşener, (meral danış) beştaş, (murat) çepni, (selahattin) demirtaş, ümit (özdağ), (canan) kaftancıoğlu |
|  | **EN→** Kurum (surname; Murat Kurum), İmamoğlu (surname; Ekrem İmamoğlu), mayor/president/chairperson, Istanbul Metropolitan Municipality (İBB), Erdoğan (surname; Recep Tayyip Erdoğan), Özel (surname; Özgür Özel), candidate, minister, Yavaş (surname; Mansur Yavaş), Bahçeli (surname; Devlet Bahçeli), Kılıçdaroğlu (surname; Kemal Kılıçdaroğlu), president, Erbakan (surname), Altınok (surname; Turgut Altınok), Akşener (surname; Meral Akşener), Beştaş (surname; Meral Danış Beştaş), Çepni (surname; Murat Çepni), Demirtaş (surname; Selahattin Demirtaş), Özdağ (surname; Ümit Özdağ), Kaftancıoğlu (surname; Canan Kaftancıoğlu) |
| **Party/Alliance** | **TR→** ak (parti), chp, iyi (parti), cumhur, millet, gelecek (partisi), mhp, ittifak, yeniden (refah), hüdapar, ysp, meclis, zafer (partisi), iktidar, muhalefet, kürt, milletvekili, istifa, dem, hdp, hükümet, saadet (partisi), muhalif |
|  | **EN→** Justice and Development Party (AK Party), Republican People’s Party (CHP), Good Party (İYİ Party), People’s Alliance, Nation Alliance, Future Party, Nationalist Movement Party (MHP), alliance, New Welfare Party, Free Cause Party (HÜDA PAR), Green Left Party (YSP), parliament/assembly, Victory Party, governing party/ruling bloc, opposition, Kurd/Kurdish, member of parliament, resignation, Peoples’ Equality and Democracy Party (DEM Party), Peoples’ Democratic Party (HDP), government, Felicity Party, opposition-aligned |
| **Election/Campaign** | **TR→** seçim, aday, seçmen, anket, miting, sonuç, vaat, kampanya, propaganda, yasak, geçersiz, geçerli, galip, baraj, sandık, mağlup, katılım, sayım, destek, fenerbahçe, galatasaray, reklam, koordinasyon, kent, araştırma, açılış, tören |
|  | **EN→** election, candidate, voter, opinion poll/survey, rally, result, promise/pledge, campaign, propaganda, ban/prohibition, invalid, valid, winner/victorious, electoral threshold, ballot box, defeated/loser, turnout/participation, vote count/counting, support, fenerbahçe, galatasaray, advertisement/campaign ad, coordination, city/urban, research/survey, opening/inauguration, ceremony |
| **Urban Transformation/**  **Earthquake** | **TR→** deprem, kentsel, konut, imar, riskli, toki, kira, rezerv, kentleşme, şehircilik, inşaat |
|  | **EN→** earthquake, urban, housing, zoning/urban development, high-risk/risky, Housing Development Administration of the Republic of Türkiye (TOKİ), rent, reserve area, urbanization, urban planning, construction |
| **Transportation/Infrastructure** | **TR→** metro, yol, metrobüs, otobüs, otopark, tünel, hat, marmaray, tramvay, köprü, ulaşım, hizray, iskele, trafik, raylı, toplu (taşıma), taksi, kanal (istanbul) |
|  | **EN→** metro/subway, road, Metrobus (bus rapid transit), bus, car park/parking lot, tunnel, line/route, Marmaray, tram, bridge, transportation, HIZRAY rapid-transit project, pier/ferry terminal, traffic, rail/rail-based, public transportation, taxi, Canal Istanbul |
| **Economy/Cost of Living** | **TR→** emekli, gelir, enflasyon, ekonomi, zam, bütçe, asgari, vergi, fiyat, kira, borç, işsizlik, yoksulluk, market, pahalılık, dolar, yerli, milli, maaş, lira, merkez (bankası), (Mehmet) şimşek, gelir, ekmek, borsa, faiz, ticaret, altın, kredi, tarım, turizm |
|  | **EN→** retiree, income, inflation, economy, price increase/price hike, budget, minimum (wage), tax, price, rent, debt, unemployment, poverty, supermarket/market, high cost of living, US dollar, domestic/local, national, salary, Turkish lira, central bank, Şimşek (surname; Mehmet Şimşek), income, bread, stock market, interest rate, trade, gold, credit/loan, agriculture, tourism |
| **Environment/Projects** | **TR→** çevre, yeşil, kanal (istanbul), hava, iklim, meydan, park, orman, ağaç, doğa, sürdürülebilir, ekoloji, karbon, emisyon, geri (dönüşüm), verimlilik, temiz, sıfır (atık) |
|  | **EN→** environment, green, Canal Istanbul, air, climate, public square, park, forest, tree, nature, sustainable, ecology, carbon, emissions, recycling, efficiency, clean, zero waste |
| **Media/Discourse** | **TR→** medya, video, haber, program, canlı, polemik, gazete, açıklama, açıkladı, yayın, tweet, basın, tartışma, gazze, algı, israil, muhalif, filistin, iftira, trol, iftar, sahur, ramazan |
|  | **EN→** media, video, news, program, live/live broadcast, polemic/controversy, newspaper, statement, announced/stated, broadcast/publication, tweet, press, debate/discussion, Gaza, perception, Israel, opposition-aligned, Palestine, defamation/slander, troll, iftar (fast-breaking meal), sahur (pre-dawn meal), Ramadan |
| **Security/Law** | **TR→** ysk, güvenlik, (seçim) güvenliği, hukuk, polis, yasa, yasak, itiraz, mahkeme, jandarma, tutanak, yargı, pkk, terör, terörist, tehdit, saldırı, adalet, yerli, milli, fetö, demokrasi, mahkum, kandil, suç, anayasa, soruşturma, bedelli (askerlik) |
|  | **EN→** Supreme Election Council (YSK), security, election security, law/legal system, police, law/statute, ban/prohibition, objection/appeal, court, gendarmerie, official record/minutes, judiciary, PKK, terrorism, terrorist, threat, attack, justice, domestic/local, national, FETÖ, democracy, convict/prisoner, Qandil Mountains, crime, constitution, investigation, paid military service |
| **Ethics/Violations** | **TR→** para (sayma), rant, ihale, skandal, israf, rüşvet, yolsuzluk, etik, usulsüzlük, kasa, kirli, muhbir, (fatih) keleş, çıkar, menfaat |
|  | **EN→** counting money, rent-seeking/unearned gain, public tender/procurement, scandal, wasteful spending, bribery, corruption, ethics/ethical, irregularity/procedural misconduct, cashbox/safe, dirty/tainted, informant, Keleş (surname; Fatih Keleş), self-interest/private interest, personal benefit/private interest |
| **Social Policies/Welfare** | **TR→** emekli, öğrenci, genç, kadın, işçi, engelli, esnaf, aile, çocuk, burs, sağlık, aile, lgbt, memur, kadro, yardım, eyt, staj, toplum, eğitim |
|  | **EN→** retiree, student, youth/young people, woman/women, worker, person/people with disabilities, tradespeople/small business owners, family, child/children, scholarship, health, family, LGBT, civil servant, public-sector post/staff position, aid/assistance, people affected by the retirement-age requirement (EYT), internship, society/community, education |

***Note.*** *TR lists the original Turkish keywords used in the analysis. EN provides context-sensitive English glosses for reader accessibility only; the English terms were not used for coding, keyword matching, or classification. Parenthetical expressions in the TR lists reproduce the original matching structure.*

**Appendix S3A. Auxiliary Transformer Benchmark for Document-Level Issue Classification**

The transformer analysis reported in this appendix was conducted during the revision as an auxiliary document-level benchmark. It was analytically separate from the dictionary-supervised, multi-indicator procedure used to generate the main 375-indicator issue matrix. Consequently, the transformer predictions were not used to produce the cross-lagged correlations, RCb values, tables, or substantive findings reported in the study.

Within this auxiliary benchmark, the 11 substantive S3 issue categories were represented in a single-label, multiclass architecture. A twelfth “None/Outside codebook” class was retained to prevent out-of-scope texts from being forced into a substantive category. Two pretrained Turkish transformers—dbmdz/bert-base-turkish-128k-cased (BERTurk) and YSKartal/berturk-social-5m—were fine-tuned on adjudicated human-coded X texts. Model selection used macro-F1 on an internal development set with a class-weighted loss. BERTurk was selected for the auxiliary benchmark. The eligible development dataset contained 1,875 items, comprising 1,580 training items and 295 development items. A separate locked test set of 200 items was opened once after the model and category mapping had been frozen.

**S3A Table 1.** Locked-Test Performance of the Auxiliary Document-Level Issue-Classification Benchmark

| **Model** | **Test n** | **Accuracy** | **Balanced accuracy** | **Macro-F1** | **Weighted-F1** |
| --- | --- | --- | --- | --- | --- |
| BERTurk | 200 | .735 | .697 | .630 | .745 |

***Note.*** *These performance values characterize only the auxiliary single-label transformer benchmark and should not be interpreted as the validation accuracy of the main dictionary-supervised, multi-indicator procedure. The previously reported 94% accuracy concerns relevance screening only. The complete 12 × 12 confusion matrix and class-wise precision, recall, F1, and support values for the auxiliary benchmark are provided in the workbook X_Nihai_Test_Raporu_v2_1.xlsx within the OSF archive 02_Classifier_Validation/X_issue_classifier_validation_issue_only_v1.zip.*

**Appendix S4. Second-Level Agenda (Attribute)**

**Conceptual note.** This codebook operationalizes second-level agenda-setting as the relative salience of predefined attributes associated with candidates, issues, and political performance. Positive (Emotional/Encouraging) and Negative (Emotional/Reactive) are affective attributes; the remaining six categories are substantive or cognitive attributes. Neutral valence is not treated as a separate specific attribute category. The matched keywords function as category indicators under the coding rules and should not be interpreted as complete frames or as standalone measures of sentiment.

**S4 Table:** Attribute Categories and Matched Keywords

| **Categories** | **Matched keywords (TR originals and EN glosses)** |
| --- | --- |
| **Positive (Emotional/Encouraging)** | **TR→** hizmet, güçlü, adil, dürüst, etkili, liyakat, şeffaf, güvenilir, kazanmak, kazanacak, kazandı, tecrübeli, başarı, deneyimli, yatırım, reform, kararlı, doğru, adalet(li), yerli, milli, alır, alacak, mutlu, umut, güven, gayret, huzur |
|  | **EN→** public service/service, strong, fair/just, honest, effective, merit, transparent, trustworthy/reliable, to win, will win, won, experienced, success, experienced, investment, reform, determined/decisive, right/correct, justice/fair, domestic/local, national, wins/is expected to win, will win, happy, hope, trust/confidence, effort, peace/social harmony |
| **Public Connection/Empathy** | **TR→** emekli, halk, vatandaş, genç, esnaf, sahada, engelli, kadın, yanında, yardım, dinledi, sahiplendi, destek, dürüst, şeffaf, tarafsız |
|  | **EN→** retiree, public/people, citizen, youth/young people, tradespeople/small business owners, on the ground/in the field, person/people with disabilities, woman/women, standing with/supporting, aid/assistance, listened, embraced/championed, support, honest, transparent, impartial |
| **Decisiveness/Crisis Management** | **TR→** deprem, mücadele, acil, başa (çıkmak), sel, afet, kriz, hızlı, kararlı, söz (vermek), hedef |
|  | **EN→** earthquake, struggle/fight, urgent/emergency, cope with/manage, flood, disaster, crisis, fast/rapid, determined/decisive, promise/make a promise, target/goal |
| **Vision/Innovation** | **TR→** gelecek, şehir, dijital, yeşil, vizyon, sürdürülebilir, akıllı, inovasyon |
|  | **EN→** future, city, digital, green, vision, sustainable, smart, innovation |
| **Negative (Emotional/Reactive)** | **TR→** zam, yalan, rant, mağdur, israf, borç, skandal, beceriksiz, yolsuzluk, mağduriyet, kriz, yetersiz, usulsüzlük, ihmal, liyakatsiz, iflas, yanlış, pahalı, kirli, sorun, ezecek, muhbir, (fatih) keleş, yazık, yeter, fetö, kaybedecek, iftira, sözde, gizli, mal (varlığı), otoriter, baskıcı, kutuplaştırıcı, liyakatsiz |
|  | **EN→** price increase/price hike, lie/falsehood, rent-seeking/unearned gain, victim/aggrieved, wasteful spending, debt, scandal, incompetent, corruption, victimization/grievance, crisis, inadequate/incompetent, irregularity/procedural misconduct, negligence, unqualified/lacking merit, bankruptcy, wrong/incorrect, expensive, dirty/tainted, problem, will crush/oppress, informant, Keleş (surname; Fatih Keleş), shame/pity, enough, FETÖ, will lose, defamation/slander, so-called, secret/hidden, assets/asset holdings, authoritarian, repressive/oppressive, polarizing, unqualified/lacking merit |
| **Experience/Competence** | **TR→** belediyecilik, icraat, şehircilik, uzman, mühendis, tecrübe, deneyim, yeterlilik, yapar, başarır, çalışkan, çalışıyor, çözüm, proje |
|  | **EN→** municipal governance/municipal administration, accomplishments/actions in office, urban planning, expert, engineer, experience, experience, competence/qualification, gets things done/can do, succeeds/can accomplish, hardworking, works/is working, solution, project |
| **Leadership/Charisma** | **TR→** lider, öncü, karizma, vizyoner, öncü, sahiplenme, önder, koordinasyon, mücadele |
|  | **EN→** leader, pioneer, charisma, visionary, pioneer, ownership/commitment, leader, coordination, struggle/fight |
| **Ideology/Partisanship** | **TR→** ideoloji, particilik, partizan, kutuplaşma, taraf, tarafgir, kürt, muhalif, yanlı, milliyetçi, laik, muhafazakar |
|  | **EN→** ideology, party politics/partisanship, partisan, polarization, side/alignment, partisan/biased, Kurd/Kurdish, opposition-aligned, pro-/aligned with, nationalist, secular, conservative |

***Note.*** *TR lists the original Turkish keywords used in the analysis. EN provides context-sensitive English glosses for reader accessibility only; the English terms were not used for coding, keyword matching, or classification. Parenthetical expressions in the TR lists reproduce the original matching structure.*

**Operational note.** The eight S4 categories provide the conceptual grouping of the matched attribute indicators. Positive (Emotional/Encouraging) and Negative (Emotional/Reactive) represent affective attributes, whereas the remaining six categories represent substantive or cognitive attributes. For the main inferential analyses, the S4 indicators were applied to both X posts and online news texts using dictionary-supervised matching. Repeated occurrences of the same indicator within an item were counted once, whereas distinct matched indicators could each contribute to the temporal frequency profile. Content without an S4 indicator match did not contribute an attribute-indicator count. The resulting ordered matrix contained 122 attribute indicators. Cross-lagged and RCb analyses were calculated from these indicator-frequency vectors rather than from vectors containing only eight aggregate category percentages, raw sentiment scores, or indicators of complete frames.

**Appendix S5. Correlation Scores (First-Level Agenda)**

**S5 Table A.** Correlations for Online News from Day 1 (AM) to Day 31 (PM) (First-Level Agenda)

| **Days** | **Day 1** | | **Day 2** | | **Day 3** | | **Day 4** | | **Day 5** | | **Day 6** | | **Day 7** | |
| --- | --- | --- | --- | --- | --- | --- | --- | --- | --- | --- | --- | --- | --- | --- |
|  | **AM** | **PM** | **AM** | **PM** | **AM** | **PM** | **AM** | **PM** | **AM** | **PM** | **AM** | **PM** | **AM** | **PM** |
| Day 1 (AM) | 1 | ,991^**^ | ,988^**^ | ,988^**^ | ,986^**^ | ,986^**^ | ,991^**^ | ,990^**^ | ,987^**^ | ,985^**^ | ,969^**^ | ,990^**^ | ,991^**^ | ,990^**^ |
| Day 1 (PM) |  | 1 | ,990^**^ | ,992^**^ | ,986^**^ | ,990^**^ | ,985^**^ | ,991^**^ | ,984^**^ | ,986^**^ | ,961^**^ | ,989^**^ | ,987^**^ | ,988^**^ |
| Day 2 (AM) |  |  | 1 | ,990^**^ | ,987^**^ | ,991^**^ | ,984^**^ | ,990^**^ | ,979^**^ | ,976^**^ | ,977^**^ | ,989^**^ | ,988^**^ | ,990^**^ |
| Day 2 (PM) |  |  |  | 1 | ,991^**^ | ,993^**^ | ,985^**^ | ,991^**^ | ,980^**^ | ,983^**^ | ,964^**^ | ,988^**^ | ,985^**^ | ,988^**^ |
| Day 3 (AM) |  |  |  |  | 1 | ,990^**^ | ,985^**^ | ,986^**^ | ,979^**^ | ,986^**^ | ,965^**^ | ,987^**^ | ,984^**^ | ,989^**^ |
| Day 3 (PM) |  |  |  |  |  | 1 | ,986^**^ | ,992^**^ | ,983^**^ | ,983^**^ | ,969^**^ | ,990^**^ | ,987^**^ | ,988^**^ |
| Day 4 (AM) |  |  |  |  |  |  | 1 | ,989^**^ | ,986^**^ | ,987^**^ | ,973^**^ | ,985^**^ | ,991^**^ | ,989^**^ |
| Day 4 (PM) |  |  |  |  |  |  |  | 1 | ,986^**^ | ,980^**^ | ,971^**^ | ,990^**^ | ,990^**^ | ,987^**^ |
| Day 5 (AM) |  |  |  |  |  |  |  |  | 1 | ,979^**^ | ,962^**^ | ,983^**^ | ,987^**^ | ,979^**^ |
| Day 5 (PM) |  |  |  |  |  |  |  |  |  | 1 | ,957^**^ | ,985^**^ | ,982^**^ | ,985^**^ |
| Day 6 (AM) |  |  |  |  |  |  |  |  |  |  | 1 | ,974^**^ | ,977^**^ | ,972^**^ |
| Day 6 (PM) |  |  |  |  |  |  |  |  |  |  |  | 1 | ,992^**^ | ,990^**^ |
| Day 7 (AM) |  |  |  |  |  |  |  |  |  |  |  |  | 1 | ,990^**^ |
| Day 7 (PM) |  |  |  |  |  |  |  |  |  |  |  |  |  | 1 |

**p<0.05, **p<0.001*

**S5 Table A.** Correlations for Online News from Day 1 (AM) to Day 31 (PM) (First-Level Agenda) **– Continued**

| **Days** | **Day 8** | | **Day 9** | | **Day 10** | | **Day 11** | | **Day 12** | | **Day 13** | | **Day 14** | |
| --- | --- | --- | --- | --- | --- | --- | --- | --- | --- | --- | --- | --- | --- | --- |
|  | **AM** | **PM** | **AM** | **PM** | **AM** | **PM** | **AM** | **PM** | **AM** | **PM** | **AM** | **PM** | **AM** | **PM** |
| Day 1 (AM) | ,982^**^ | ,962^**^ | ,962^**^ | ,982^**^ | ,991^**^ | ,974^**^ | ,982^**^ | ,971^**^ | ,982^**^ | ,976^**^ | ,981^**^ | ,987^**^ | ,976^**^ | ,989^**^ |
| Day 1 (PM) | ,974^**^ | ,966^**^ | ,956^**^ | ,987^**^ | ,989^**^ | ,976^**^ | ,981^**^ | ,973^**^ | ,975^**^ | ,976^**^ | ,977^**^ | ,988^**^ | ,967^**^ | ,986^**^ |
| Day 2 (AM) | ,986^**^ | ,964^**^ | ,971^**^ | ,983^**^ | ,987^**^ | ,983^**^ | ,987^**^ | ,985^**^ | ,981^**^ | ,984^**^ | ,975^**^ | ,979^**^ | ,971^**^ | ,980^**^ |
| Day 2 (PM) | ,976^**^ | ,966^**^ | ,958^**^ | ,986^**^ | ,986^**^ | ,979^**^ | ,981^**^ | ,978^**^ | ,974^**^ | ,977^**^ | ,975^**^ | ,984^**^ | ,964^**^ | ,982^**^ |
| Day 3 (AM) | ,976^**^ | ,963^**^ | ,955^**^ | ,984^**^ | ,984^**^ | ,977^**^ | ,979^**^ | ,975^**^ | ,973^**^ | ,975^**^ | ,974^**^ | ,982^**^ | ,965^**^ | ,980^**^ |
| Day 3 (PM) | ,978^**^ | ,971^**^ | ,963^**^ | ,985^**^ | ,986^**^ | ,983^**^ | ,982^**^ | ,979^**^ | ,973^**^ | ,981^**^ | ,973^**^ | ,981^**^ | ,964^**^ | ,980^**^ |
| Day 4 (AM) | ,982^**^ | ,973^**^ | ,967^**^ | ,982^**^ | ,990^**^ | ,975^**^ | ,987^**^ | ,971^**^ | ,980^**^ | ,975^**^ | ,982^**^ | ,985^**^ | ,975^**^ | ,989^**^ |
| Day 4 (PM) | ,981^**^ | ,966^**^ | ,967^**^ | ,986^**^ | ,990^**^ | ,982^**^ | ,987^**^ | ,976^**^ | ,974^**^ | ,978^**^ | ,974^**^ | ,983^**^ | ,969^**^ | ,984^**^ |
| Day 5 (AM) | ,976^**^ | ,968^**^ | ,961^**^ | ,978^**^ | ,989^**^ | ,970^**^ | ,979^**^ | ,962^**^ | ,971^**^ | ,969^**^ | ,976^**^ | ,979^**^ | ,968^**^ | ,980^**^ |
| Day 5 (PM) | ,966^**^ | ,973^**^ | ,950^**^ | ,984^**^ | ,983^**^ | ,967^**^ | ,970^**^ | ,963^**^ | ,967^**^ | ,966^**^ | ,978^**^ | ,989^**^ | ,963^**^ | ,983^**^ |
| Day 6 (AM) | ,983^**^ | ,949^**^ | ,978^**^ | ,960^**^ | ,972^**^ | ,971^**^ | ,975^**^ | ,965^**^ | ,966^**^ | ,966^**^ | ,951^**^ | ,952^**^ | ,958^**^ | ,960^**^ |
| Day 6 (PM) | ,981^**^ | ,971^**^ | ,969^**^ | ,984^**^ | ,987^**^ | ,979^**^ | ,980^**^ | ,973^**^ | ,971^**^ | ,977^**^ | ,975^**^ | ,983^**^ | ,969^**^ | ,982^**^ |
| Day 7 (AM) | ,988^**^ | ,974^**^ | ,978^**^ | ,981^**^ | ,992^**^ | ,978^**^ | ,989^**^ | ,974^**^ | ,978^**^ | ,977^**^ | ,979^**^ | ,982^**^ | ,974^**^ | ,983^**^ |
| Day 7 (PM) | ,987^**^ | ,973^**^ | ,968^**^ | ,978^**^ | ,988^**^ | ,972^**^ | ,983^**^ | ,977^**^ | ,979^**^ | ,978^**^ | ,977^**^ | ,981^**^ | ,967^**^ | ,982^**^ |
| Day 8 (AM) | 1 | ,963^**^ | ,982^**^ | ,965^**^ | ,985^**^ | ,969^**^ | ,984^**^ | ,974^**^ | ,978^**^ | ,973^**^ | ,967^**^ | ,963^**^ | ,968^**^ | ,969^**^ |
| Day 8 (PM) |  | 1 | ,966^**^ | ,966^**^ | ,969^**^ | ,959^**^ | ,961^**^ | ,954^**^ | ,951^**^ | ,956^**^ | ,965^**^ | ,961^**^ | ,946^**^ | ,958^**^ |
| Day 9 (AM) |  |  | 1 | ,959^**^ | ,973^**^ | ,968^**^ | ,977^**^ | ,964^**^ | ,958^**^ | ,963^**^ | ,950^**^ | ,948^**^ | ,956^**^ | ,949^**^ |
| Day 9 (PM) |  |  |  | 1 | ,984^**^ | ,987^**^ | ,977^**^ | ,969^**^ | ,969^**^ | ,975^**^ | ,976^**^ | ,988^**^ | ,970^**^ | ,983^**^ |
| Day 10 (AM) |  |  |  |  | 1 | ,978^**^ | ,989^**^ | ,973^**^ | ,980^**^ | ,977^**^ | ,980^**^ | ,985^**^ | ,977^**^ | ,987^**^ |
| Day 10 (PM) |  |  |  |  |  | 1 | ,978^**^ | ,974^**^ | ,967^**^ | ,979^**^ | ,967^**^ | ,973^**^ | ,968^**^ | ,972^**^ |
| Day 11 (AM) |  |  |  |  |  |  | 1 | ,981^**^ | ,982^**^ | ,979^**^ | ,974^**^ | ,974^**^ | ,976^**^ | ,976^**^ |
| Day 11 (PM) |  |  |  |  |  |  |  | 1 | ,982^**^ | ,989^**^ | ,973^**^ | ,967^**^ | ,973^**^ | ,965^**^ |
| Day 12 (AM) |  |  |  |  |  |  |  |  | 1 | ,984^**^ | ,984^**^ | ,973^**^ | ,984^**^ | ,977^**^ |
| Day 12 (PM) |  |  |  |  |  |  |  |  |  | 1 | ,985^**^ | ,974^**^ | ,985^**^ | ,975^**^ |
| Day 13 (AM) |  |  |  |  |  |  |  |  |  |  | 1 | ,983^**^ | ,988^**^ | ,983^**^ |
| Day 13 (PM) |  |  |  |  |  |  |  |  |  |  |  | 1 | ,975^**^ | ,988^**^ |
| Day 14 (AM) |  |  |  |  |  |  |  |  |  |  |  |  | 1 | ,979^**^ |
| Day 14 (PM) |  |  |  |  |  |  |  |  |  |  |  |  |  | 1 |

**p<0.05, **p<0.001*

**S5 Table A.** Correlations for Online News from Day 1 (AM) to Day 31 (PM) (First-Level Agenda) – **Continued**

| **Days** | **Day 15** | | **Day 16** | | **Day 17** | | **Day 18** | | **Day 19** | | **Day 20** | | **Day 21** | |
| --- | --- | --- | --- | --- | --- | --- | --- | --- | --- | --- | --- | --- | --- | --- |
|  | **AM** | **PM** | **AM** | **PM** | **AM** | **PM** | **AM** | **PM** | **AM** | **PM** | **AM** | **PM** | **AM** | **PM** |
| Day 1 (AM) | ,988^**^ | ,986^**^ | ,986^**^ | ,988^**^ | ,991^**^ | ,986^**^ | ,988^**^ | ,975^**^ | ,972^**^ | ,986^**^ | ,979^**^ | ,984^**^ | ,989^**^ | ,985^**^ |
| Day 1 (PM) | ,980^**^ | ,989^**^ | ,983^**^ | ,990^**^ | ,986^**^ | ,985^**^ | ,986^**^ | ,975^**^ | ,969^**^ | ,986^**^ | ,974^**^ | ,989^**^ | ,983^**^ | ,984^**^ |
| Day 2 (AM) | ,981^**^ | ,989^**^ | ,982^**^ | ,983^**^ | ,989^**^ | ,979^**^ | ,985^**^ | ,976^**^ | ,966^**^ | ,982^**^ | ,975^**^ | ,986^**^ | ,985^**^ | ,987^**^ |
| Day 2 (PM) | ,976^**^ | ,989^**^ | ,981^**^ | ,988^**^ | ,986^**^ | ,983^**^ | ,987^**^ | ,977^**^ | ,966^**^ | ,986^**^ | ,969^**^ | ,986^**^ | ,982^**^ | ,985^**^ |
| Day 3 (AM) | ,977^**^ | ,983^**^ | ,983^**^ | ,987^**^ | ,987^**^ | ,983^**^ | ,989^**^ | ,978^**^ | ,964^**^ | ,986^**^ | ,971^**^ | ,982^**^ | ,986^**^ | ,986^**^ |
| Day 3 (PM) | ,975^**^ | ,987^**^ | ,983^**^ | ,985^**^ | ,986^**^ | ,983^**^ | ,984^**^ | ,975^**^ | ,964^**^ | ,985^**^ | ,970^**^ | ,986^**^ | ,982^**^ | ,985^**^ |
| Day 4 (AM) | ,986^**^ | ,985^**^ | ,988^**^ | ,985^**^ | ,991^**^ | ,985^**^ | ,987^**^ | ,976^**^ | ,970^**^ | ,986^**^ | ,978^**^ | ,985^**^ | ,988^**^ | ,987^**^ |
| Day 4 (PM) | ,980^**^ | ,987^**^ | ,984^**^ | ,984^**^ | ,987^**^ | ,985^**^ | ,983^**^ | ,973^**^ | ,964^**^ | ,987^**^ | ,973^**^ | ,985^**^ | ,983^**^ | ,983^**^ |
| Day 5 (AM) | ,979^**^ | ,980^**^ | ,983^**^ | ,978^**^ | ,985^**^ | ,978^**^ | ,976^**^ | ,961^**^ | ,962^**^ | ,976^**^ | ,973^**^ | ,979^**^ | ,980^**^ | ,978^**^ |
| Day 5 (PM) | ,975^**^ | ,979^**^ | ,978^**^ | ,988^**^ | ,980^**^ | ,985^**^ | ,985^**^ | ,973^**^ | ,965^**^ | ,984^**^ | ,967^**^ | ,978^**^ | ,981^**^ | ,978^**^ |
| Day 6 (AM) | ,965^**^ | ,963^**^ | ,965^**^ | ,952^**^ | ,973^**^ | ,956^**^ | ,961^**^ | ,950^**^ | ,940^**^ | ,961^**^ | ,962^**^ | ,966^**^ | ,969^**^ | ,968^**^ |
| Day 6 (PM) | ,979^**^ | ,983^**^ | ,980^**^ | ,983^**^ | ,986^**^ | ,983^**^ | ,983^**^ | ,974^**^ | ,964^**^ | ,986^**^ | ,971^**^ | ,982^**^ | ,983^**^ | ,982^**^ |
| Day 7 (AM) | ,985^**^ | ,982^**^ | ,986^**^ | ,981^**^ | ,989^**^ | ,983^**^ | ,983^**^ | ,969^**^ | ,963^**^ | ,982^**^ | ,978^**^ | ,983^**^ | ,988^**^ | ,986^**^ |
| Day 7 (PM) | ,981^**^ | ,985^**^ | ,982^**^ | ,984^**^ | ,986^**^ | ,979^**^ | ,989^**^ | ,981^**^ | ,968^**^ | ,985^**^ | ,974^**^ | ,983^**^ | ,987^**^ | ,988^**^ |
| Day 8 (AM) | ,976^**^ | ,976^**^ | ,976^**^ | ,966^**^ | ,982^**^ | ,963^**^ | ,972^**^ | ,963^**^ | ,957^**^ | ,969^**^ | ,971^**^ | ,973^**^ | ,979^**^ | ,981^**^ |
| Day 8 (PM) | ,954^**^ | ,966^**^ | ,954^**^ | ,963^**^ | ,959^**^ | ,960^**^ | ,961^**^ | ,954^**^ | ,942^**^ | ,961^**^ | ,944^**^ | ,957^**^ | ,957^**^ | ,960^**^ |
| Day 9 (AM) | ,960^**^ | ,959^**^ | ,955^**^ | ,947^**^ | ,964^**^ | ,953^**^ | ,951^**^ | ,943^**^ | ,933^**^ | ,954^**^ | ,951^**^ | ,954^**^ | ,956^**^ | ,959^**^ |
| Day 9 (PM) | ,976^**^ | ,983^**^ | ,979^**^ | ,990^**^ | ,985^**^ | ,990^**^ | ,983^**^ | ,976^**^ | ,964^**^ | ,987^**^ | ,967^**^ | ,982^**^ | ,979^**^ | ,979^**^ |
| Day 10 (AM) | ,988^**^ | ,985^**^ | ,988^**^ | ,983^**^ | ,991^**^ | ,984^**^ | ,982^**^ | ,972^**^ | ,969^**^ | ,984^**^ | ,979^**^ | ,984^**^ | ,987^**^ | ,986^**^ |
| Day 10 (PM) | ,970^**^ | ,977^**^ | ,973^**^ | ,975^**^ | ,980^**^ | ,982^**^ | ,973^**^ | ,970^**^ | ,954^**^ | ,981^**^ | ,962^**^ | ,976^**^ | ,973^**^ | ,974^**^ |
| Day 11 (AM) | ,983^**^ | ,983^**^ | ,983^**^ | ,974^**^ | ,986^**^ | ,976^**^ | ,980^**^ | ,969^**^ | ,965^**^ | ,975^**^ | ,980^**^ | ,984^**^ | ,984^**^ | ,985^**^ |
| Day 11 (PM) | ,972^**^ | ,985^**^ | ,969^**^ | ,970^**^ | ,976^**^ | ,967^**^ | ,976^**^ | ,978^**^ | ,968^**^ | ,973^**^ | ,972^**^ | ,977^**^ | ,975^**^ | ,980^**^ |
| Day 12 (AM) | ,988^**^ | ,982^**^ | ,980^**^ | ,975^**^ | ,985^**^ | ,968^**^ | ,980^**^ | ,976^**^ | ,977^**^ | ,974^**^ | ,985^**^ | ,980^**^ | ,986^**^ | ,987^**^ |
| Day 12 (PM) | ,982^**^ | ,989^**^ | ,976^**^ | ,977^**^ | ,985^**^ | ,972^**^ | ,979^**^ | ,986^**^ | ,978^**^ | ,981^**^ | ,981^**^ | ,982^**^ | ,980^**^ | ,985^**^ |
| Day 13 (AM) | ,988^**^ | ,986^**^ | ,980^**^ | ,983^**^ | ,987^**^ | ,977^**^ | ,983^**^ | ,982^**^ | ,986^**^ | ,980^**^ | ,984^**^ | ,980^**^ | ,985^**^ | ,985^**^ |
| Day 13 (PM) | ,983^**^ | ,984^**^ | ,983^**^ | ,991^**^ | ,986^**^ | ,989^**^ | ,987^**^ | ,977^**^ | ,971^**^ | ,986^**^ | ,975^**^ | ,985^**^ | ,984^**^ | ,982^**^ |
| Day 14 (AM) | ,989^**^ | ,982^**^ | ,977^**^ | ,973^**^ | ,984^**^ | ,972^**^ | ,974^**^ | ,976^**^ | ,980^**^ | ,977^**^ | ,987^**^ | ,976^**^ | ,981^**^ | ,978^**^ |
| Day 14 (PM) | ,991^**^ | ,985^**^ | ,989^**^ | ,989^**^ | ,990^**^ | ,988^**^ | ,985^**^ | ,975^**^ | ,972^**^ | ,991^**^ | ,980^**^ | ,984^**^ | ,986^**^ | ,986^**^ |
| Day 15 (AM) | 1 | ,986^**^ | ,990^**^ | ,983^**^ | ,992^**^ | ,981^**^ | ,984^**^ | ,977^**^ | ,980^**^ | ,984^**^ | ,989^**^ | ,983^**^ | ,991^**^ | ,989^**^ |
| Day 15 (PM) |  | 1 | ,983^**^ | ,987^**^ | ,988^**^ | ,981^**^ | ,986^**^ | ,987^**^ | ,984^**^ | ,986^**^ | ,982^**^ | ,989^**^ | ,984^**^ | ,988^**^ |
| Day 16 (AM) |  |  | 1 | ,984^**^ | ,992^**^ | ,986^**^ | ,984^**^ | ,970^**^ | ,970^**^ | ,985^**^ | ,985^**^ | ,986^**^ | ,990^**^ | ,991^**^ |
| Day 16 (PM) |  |  |  | 1 | ,988^**^ | ,991^**^ | ,989^**^ | ,982^**^ | ,972^**^ | ,990^**^ | ,973^**^ | ,984^**^ | ,984^**^ | ,985^**^ |
| Day 17 (AM) |  |  |  |  | 1 | ,985^**^ | ,988^**^ | ,980^**^ | ,977^**^ | ,987^**^ | ,986^**^ | ,988^**^ | ,993^**^ | ,993^**^ |
| Day 17 (PM) |  |  |  |  |  | 1 | ,987^**^ | ,974^**^ | ,964^**^ | ,992^**^ | ,972^**^ | ,980^**^ | ,982^**^ | ,980^**^ |
| Day 18 (AM) |  |  |  |  |  |  | 1 | ,986^**^ | ,975^**^ | ,989^**^ | ,980^**^ | ,985^**^ | ,989^**^ | ,988^**^ |
| Day 18 (PM) |  |  |  |  |  |  |  | 1 | ,984^**^ | ,985^**^ | ,975^**^ | ,979^**^ | ,980^**^ | ,981^**^ |
| Day 19 (AM) |  |  |  |  |  |  |  |  | 1 | ,972^**^ | ,980^**^ | ,973^**^ | ,976^**^ | ,978^**^ |
| Day 19 (PM) |  |  |  |  |  |  |  |  |  | 1 | ,976^**^ | ,984^**^ | ,984^**^ | ,984^**^ |
| Day 20 (AM) |  |  |  |  |  |  |  |  |  |  | 1 | ,985^**^ | ,987^**^ | ,986^**^ |
| Day 20 (PM) |  |  |  |  |  |  |  |  |  |  |  | 1 | ,987^**^ | ,988^**^ |
| Day 21 (AM) |  |  |  |  |  |  |  |  |  |  |  |  | 1 | ,994^**^ |
| Day 21 (PM) |  |  |  |  |  |  |  |  |  |  |  |  |  | 1 |

**p<0.05, **p<0.001*

**S5 Table A.** Correlations for Online News from Day 1 (AM) to Day 31 (PM) (First-Level Agenda) – **Continued**

| **Days** | **Day 22** | | **Day 23** | | **Day 24** | | **Day 25** | | **Day 26** | | **Day 27** | | **Day 28** | |
| --- | --- | --- | --- | --- | --- | --- | --- | --- | --- | --- | --- | --- | --- | --- |
|  | **AM** | **PM** | **AM** | **PM** | **AM** | **PM** | **AM** | **PM** | **AM** | **PM** | **AM** | **PM** | **AM** | **PM** |
| Day 1 (AM) | ,986^**^ | ,982^**^ | ,983^**^ | ,986^**^ | ,980^**^ | ,973^**^ | ,984^**^ | ,982^**^ | ,988^**^ | ,986^**^ | ,978^**^ | ,990^**^ | ,984^**^ | ,989^**^ |
| Day 1 (PM) | ,986^**^ | ,981^**^ | ,982^**^ | ,989^**^ | ,981^**^ | ,977^**^ | ,982^**^ | ,981^**^ | ,983^**^ | ,985^**^ | ,980^**^ | ,988^**^ | ,980^**^ | ,986^**^ |
| Day 2 (AM) | ,984^**^ | ,980^**^ | ,980^**^ | ,982^**^ | ,981^**^ | ,977^**^ | ,984^**^ | ,985^**^ | ,987^**^ | ,987^**^ | ,988^**^ | ,986^**^ | ,985^**^ | ,986^**^ |
| Day 2 (PM) | ,983^**^ | ,981^**^ | ,981^**^ | ,989^**^ | ,981^**^ | ,983^**^ | ,983^**^ | ,984^**^ | ,984^**^ | ,988^**^ | ,981^**^ | ,989^**^ | ,982^**^ | ,985^**^ |
| Day 3 (AM) | ,983^**^ | ,981^**^ | ,981^**^ | ,987^**^ | ,981^**^ | ,981^**^ | ,986^**^ | ,983^**^ | ,983^**^ | ,990^**^ | ,979^**^ | ,989^**^ | ,985^**^ | ,987^**^ |
| Day 3 (PM) | ,983^**^ | ,979^**^ | ,981^**^ | ,989^**^ | ,982^**^ | ,984^**^ | ,983^**^ | ,985^**^ | ,983^**^ | ,988^**^ | ,984^**^ | ,988^**^ | ,984^**^ | ,984^**^ |
| Day 4 (AM) | ,986^**^ | ,983^**^ | ,985^**^ | ,987^**^ | ,983^**^ | ,978^**^ | ,989^**^ | ,982^**^ | ,989^**^ | ,988^**^ | ,981^**^ | ,990^**^ | ,987^**^ | ,989^**^ |
| Day 4 (PM) | ,984^**^ | ,979^**^ | ,980^**^ | ,987^**^ | ,981^**^ | ,980^**^ | ,983^**^ | ,983^**^ | ,986^**^ | ,985^**^ | ,982^**^ | ,988^**^ | ,984^**^ | ,985^**^ |
| Day 5 (AM) | ,983^**^ | ,972^**^ | ,975^**^ | ,981^**^ | ,977^**^ | ,968^**^ | ,975^**^ | ,974^**^ | ,982^**^ | ,979^**^ | ,974^**^ | ,981^**^ | ,979^**^ | ,981^**^ |
| Day 5 (PM) | ,980^**^ | ,977^**^ | ,978^**^ | ,985^**^ | ,973^**^ | ,971^**^ | ,979^**^ | ,976^**^ | ,977^**^ | ,983^**^ | ,967^**^ | ,984^**^ | ,974^**^ | ,984^**^ |
| Day 6 (AM) | ,962^**^ | ,958^**^ | ,958^**^ | ,956^**^ | ,960^**^ | ,954^**^ | ,969^**^ | ,964^**^ | ,967^**^ | ,968^**^ | ,974^**^ | ,967^**^ | ,974^**^ | ,968^**^ |
| Day 6 (PM) | ,980^**^ | ,979^**^ | ,977^**^ | ,984^**^ | ,976^**^ | ,975^**^ | ,982^**^ | ,982^**^ | ,982^**^ | ,986^**^ | ,980^**^ | ,985^**^ | ,980^**^ | ,986^**^ |
| Day 7 (AM) | ,984^**^ | ,978^**^ | ,980^**^ | ,982^**^ | ,981^**^ | ,974^**^ | ,987^**^ | ,981^**^ | ,986^**^ | ,987^**^ | ,982^**^ | ,987^**^ | ,987^**^ | ,987^**^ |
| Day 7 (PM) | ,980^**^ | ,983^**^ | ,982^**^ | ,982^**^ | ,979^**^ | ,977^**^ | ,990^**^ | ,986^**^ | ,986^**^ | ,989^**^ | ,980^**^ | ,992^**^ | ,986^**^ | ,986^**^ |
| Day 8 (AM) | ,973^**^ | ,969^**^ | ,971^**^ | ,966^**^ | ,971^**^ | ,962^**^ | ,980^**^ | ,976^**^ | ,980^**^ | ,978^**^ | ,980^**^ | ,980^**^ | ,982^**^ | ,976^**^ |
| Day 8 (PM) | ,957^**^ | ,956^**^ | ,955^**^ | ,964^**^ | ,953^**^ | ,958^**^ | ,959^**^ | ,961^**^ | ,960^**^ | ,961^**^ | ,955^**^ | ,963^**^ | ,955^**^ | ,960^**^ |
| Day 9 (AM) | ,953^**^ | ,950^**^ | ,946^**^ | ,948^**^ | ,951^**^ | ,947^**^ | ,962^**^ | ,961^**^ | ,964^**^ | ,958^**^ | ,966^**^ | ,958^**^ | ,962^**^ | ,958^**^ |
| Day 9 (PM) | ,983^**^ | ,980^**^ | ,977^**^ | ,990^**^ | ,977^**^ | ,979^**^ | ,976^**^ | ,978^**^ | ,980^**^ | ,983^**^ | ,975^**^ | ,982^**^ | ,976^**^ | ,987^**^ |
| Day 10 (AM) | ,987^**^ | ,980^**^ | ,983^**^ | ,986^**^ | ,984^**^ | ,974^**^ | ,985^**^ | ,981^**^ | ,988^**^ | ,985^**^ | ,983^**^ | ,989^**^ | ,986^**^ | ,987^**^ |
| Day 10 (PM) | ,977^**^ | ,972^**^ | ,967^**^ | ,980^**^ | ,972^**^ | ,978^**^ | ,971^**^ | ,977^**^ | ,978^**^ | ,976^**^ | ,977^**^ | ,974^**^ | ,973^**^ | ,980^**^ |
| Day 11 (AM) | ,984^**^ | ,977^**^ | ,977^**^ | ,978^**^ | ,982^**^ | ,975^**^ | ,987^**^ | ,982^**^ | ,988^**^ | ,985^**^ | ,986^**^ | ,984^**^ | ,987^**^ | ,981^**^ |
| Day 11 (PM) | ,977^**^ | ,978^**^ | ,971^**^ | ,968^**^ | ,976^**^ | ,971^**^ | ,976^**^ | ,987^**^ | ,984^**^ | ,978^**^ | ,984^**^ | ,977^**^ | ,974^**^ | ,975^**^ |
| Day 12 (AM) | ,984^**^ | ,982^**^ | ,980^**^ | ,971^**^ | ,980^**^ | ,965^**^ | ,981^**^ | ,981^**^ | ,988^**^ | ,979^**^ | ,981^**^ | ,982^**^ | ,982^**^ | ,982^**^ |
| Day 12 (PM) | ,985^**^ | ,989^**^ | ,980^**^ | ,976^**^ | ,983^**^ | ,973^**^ | ,979^**^ | ,991^**^ | ,990^**^ | ,982^**^ | ,989^**^ | ,983^**^ | ,979^**^ | ,981^**^ |
| Day 13 (AM) | ,988^**^ | ,988^**^ | ,985^**^ | ,979^**^ | ,983^**^ | ,968^**^ | ,979^**^ | ,983^**^ | ,989^**^ | ,981^**^ | ,977^**^ | ,983^**^ | ,975^**^ | ,985^**^ |
| Day 13 (PM) | ,987^**^ | ,983^**^ | ,982^**^ | ,988^**^ | ,979^**^ | ,974^**^ | ,980^**^ | ,979^**^ | ,983^**^ | ,985^**^ | ,972^**^ | ,985^**^ | ,976^**^ | ,988^**^ |
| Day 14 (AM) | ,985^**^ | ,982^**^ | ,977^**^ | ,969^**^ | ,979^**^ | ,961^**^ | ,974^**^ | ,983^**^ | ,988^**^ | ,972^**^ | ,977^**^ | ,975^**^ | ,972^**^ | ,980^**^ |
| Day 14 (PM) | ,989^**^ | ,986^**^ | ,988^**^ | ,990^**^ | ,987^**^ | ,975^**^ | ,985^**^ | ,979^**^ | ,987^**^ | ,985^**^ | ,978^**^ | ,990^**^ | ,983^**^ | ,991^**^ |
| Day 15 (AM) | ,990^**^ | ,987^**^ | ,986^**^ | ,981^**^ | ,986^**^ | ,970^**^ | ,988^**^ | ,984^**^ | ,992^**^ | ,984^**^ | ,983^**^ | ,987^**^ | ,985^**^ | ,990^**^ |
| Day 15 (PM) | ,991^**^ | ,989^**^ | ,984^**^ | ,986^**^ | ,985^**^ | ,980^**^ | ,983^**^ | ,992^**^ | ,993^**^ | ,987^**^ | ,986^**^ | ,988^**^ | ,980^**^ | ,986^**^ |
| Day 16 (AM) | ,989^**^ | ,982^**^ | ,989^**^ | ,988^**^ | ,991^**^ | ,976^**^ | ,989^**^ | ,979^**^ | ,988^**^ | ,989^**^ | ,984^**^ | ,992^**^ | ,993^**^ | ,991^**^ |
| Day 16 (PM) | ,987^**^ | ,986^**^ | ,985^**^ | ,991^**^ | ,983^**^ | ,979^**^ | ,983^**^ | ,982^**^ | ,984^**^ | ,988^**^ | ,974^**^ | ,989^**^ | ,979^**^ | ,990^**^ |
| Day 17 (AM) | ,992^**^ | ,988^**^ | ,990^**^ | ,987^**^ | ,989^**^ | ,977^**^ | ,989^**^ | ,986^**^ | ,992^**^ | ,991^**^ | ,988^**^ | ,992^**^ | ,991^**^ | ,993^**^ |
| Day 17 (PM) | ,984^**^ | ,979^**^ | ,980^**^ | ,990^**^ | ,979^**^ | ,979^**^ | ,981^**^ | ,980^**^ | ,982^**^ | ,982^**^ | ,971^**^ | ,985^**^ | ,977^**^ | ,989^**^ |
| Day 18 (AM) | ,987^**^ | ,988^**^ | ,989^**^ | ,988^**^ | ,984^**^ | ,982^**^ | ,990^**^ | ,986^**^ | ,987^**^ | ,991^**^ | ,979^**^ | ,991^**^ | ,984^**^ | ,989^**^ |
| Day 18 (PM) | ,981^**^ | ,991^**^ | ,980^**^ | ,978^**^ | ,976^**^ | ,974^**^ | ,979^**^ | ,991^**^ | ,985^**^ | ,983^**^ | ,976^**^ | ,983^**^ | ,972^**^ | ,981^**^ |
| Day 19 (AM) | ,981^**^ | ,986^**^ | ,979^**^ | ,970^**^ | ,974^**^ | ,959^**^ | ,970^**^ | ,982^**^ | ,984^**^ | ,972^**^ | ,972^**^ | ,975^**^ | ,965^**^ | ,976^**^ |
| Day 19 (PM) | ,985^**^ | ,988^**^ | ,985^**^ | ,991^**^ | ,986^**^ | ,984^**^ | ,986^**^ | ,987^**^ | ,986^**^ | ,986^**^ | ,979^**^ | ,990^**^ | ,981^**^ | ,990^**^ |
| Day 20 (AM) | ,988^**^ | ,983^**^ | ,985^**^ | ,973^**^ | ,985^**^ | ,966^**^ | ,983^**^ | ,981^**^ | ,988^**^ | ,981^**^ | ,981^**^ | ,984^**^ | ,984^**^ | ,983^**^ |
| Day 20 (PM) | ,990^**^ | ,984^**^ | ,985^**^ | ,987^**^ | ,988^**^ | ,981^**^ | ,986^**^ | ,985^**^ | ,987^**^ | ,989^**^ | ,986^**^ | ,989^**^ | ,988^**^ | ,987^**^ |
| Day 21 (AM) | ,990^**^ | ,987^**^ | ,990^**^ | ,985^**^ | ,989^**^ | ,978^**^ | ,993^**^ | ,984^**^ | ,991^**^ | ,992^**^ | ,986^**^ | ,991^**^ | ,992^**^ | ,992^**^ |
| Day 21 (PM) | ,990^**^ | ,990^**^ | ,992^**^ | ,986^**^ | ,991^**^ | ,979^**^ | ,993^**^ | ,987^**^ | ,992^**^ | ,994^**^ | ,991^**^ | ,994^**^ | ,994^**^ | ,993^**^ |
| Day 22 (AM) | 1 | ,989^**^ | ,990^**^ | ,988^**^ | ,990^**^ | ,979^**^ | ,985^**^ | ,987^**^ | ,993^**^ | ,989^**^ | ,986^**^ | ,989^**^ | ,986^**^ | ,991^**^ |
| Day 22 (PM) |  | 1 | ,990^**^ | ,985^**^ | ,987^**^ | ,978^**^ | ,986^**^ | ,991^**^ | ,992^**^ | ,988^**^ | ,985^**^ | ,991^**^ | ,983^**^ | ,989^**^ |
| Day 23 (AM) |  |  | 1 | ,987^**^ | ,992^**^ | ,975^**^ | ,989^**^ | ,982^**^ | ,987^**^ | ,989^**^ | ,984^**^ | ,991^**^ | ,987^**^ | ,989^**^ |
| Day 23 (PM) |  |  |  | 1 | ,989^**^ | ,991^**^ | ,987^**^ | ,983^**^ | ,985^**^ | ,990^**^ | ,980^**^ | ,992^**^ | ,984^**^ | ,989^**^ |
| Day 24 (AM) |  |  |  |  | 1 | ,983^**^ | ,990^**^ | ,983^**^ | ,989^**^ | ,989^**^ | ,989^**^ | ,991^**^ | ,990^**^ | ,987^**^ |
| Day 24 (PM) |  |  |  |  |  | 1 | ,985^**^ | ,981^**^ | ,982^**^ | ,984^**^ | ,977^**^ | ,984^**^ | ,979^**^ | ,978^**^ |
| Day 25 (AM) |  |  |  |  |  |  | 1 | ,986^**^ | ,989^**^ | ,993^**^ | ,986^**^ | ,994^**^ | ,993^**^ | ,989^**^ |
| Day 25 (PM) |  |  |  |  |  |  |  | 1 | ,993^**^ | ,987^**^ | ,986^**^ | ,989^**^ | ,982^**^ | ,985^**^ |
| Day 26 (AM) |  |  |  |  |  |  |  |  | 1 | ,989^**^ | ,989^**^ | ,991^**^ | ,988^**^ | ,989^**^ |
| Day 26 (PM) |  |  |  |  |  |  |  |  |  | 1 | ,988^**^ | ,995^**^ | ,992^**^ | ,991^**^ |
| Day 27 (AM) |  |  |  |  |  |  |  |  |  |  | 1 | ,987^**^ | ,989^**^ | ,984^**^ |
| Day 27 (PM) |  |  |  |  |  |  |  |  |  |  |  | 1 | ,994^**^ | ,992^**^ |
| Day 28 (AM) |  |  |  |  |  |  |  |  |  |  |  |  | 1 | ,989^**^ |
| Day 28 (PM) |  |  |  |  |  |  |  |  |  |  |  |  |  | 1 |

**p<0.05, **p<0.001*

**S5 Table A.** Correlations for Online News from Day 1 (AM) to Day 31 (PM) (First-Level Agenda) – **Continued**

| **Days** | **Day 29** | | **Day 30** | | **Day 31** | |
| --- | --- | --- | --- | --- | --- | --- |
|  | **AM** | **PM** | **AM** | **PM** | **AM** | **PM** |
| Day 1 (AM) | ,982^**^ | ,983^**^ | ,979^**^ | ,951^**^ | ,896^**^ | ,844^**^ |
| Day 1 (PM) | ,977^**^ | ,982^**^ | ,973^**^ | ,951^**^ | ,890^**^ | ,832^**^ |
| Day 2 (AM) | ,980^**^ | ,982^**^ | ,978^**^ | ,960^**^ | ,905^**^ | ,859^**^ |
| Day 2 (PM) | ,978^**^ | ,984^**^ | ,974^**^ | ,957^**^ | ,894^**^ | ,838^**^ |
| Day 3 (AM) | ,980^**^ | ,985^**^ | ,979^**^ | ,960^**^ | ,901^**^ | ,847^**^ |
| Day 3 (PM) | ,978^**^ | ,985^**^ | ,975^**^ | ,964^**^ | ,900^**^ | ,848^**^ |
| Day 4 (AM) | ,986^**^ | ,988^**^ | ,981^**^ | ,962^**^ | ,906^**^ | ,858^**^ |
| Day 4 (PM) | ,980^**^ | ,986^**^ | ,975^**^ | ,960^**^ | ,899^**^ | ,850^**^ |
| Day 5 (AM) | ,976^**^ | ,978^**^ | ,965^**^ | ,946^**^ | ,892^**^ | ,840^**^ |
| Day 5 (PM) | ,973^**^ | ,978^**^ | ,972^**^ | ,945^**^ | ,888^**^ | ,824^**^ |
| Day 6 (AM) | ,965^**^ | ,965^**^ | ,962^**^ | ,956^**^ | ,913^**^ | ,883^**^ |
| Day 6 (PM) | ,975^**^ | ,980^**^ | ,973^**^ | ,953^**^ | ,900^**^ | ,846^**^ |
| Day 7 (AM) | ,984^**^ | ,985^**^ | ,981^**^ | ,963^**^ | ,915^**^ | ,869^**^ |
| Day 7 (PM) | ,982^**^ | ,985^**^ | ,984^**^ | ,964^**^ | ,906^**^ | ,859^**^ |
| Day 8 (AM) | ,975^**^ | ,976^**^ | ,975^**^ | ,965^**^ | ,911^**^ | ,880^**^ |
| Day 8 (PM) | ,951^**^ | ,959^**^ | ,950^**^ | ,935^**^ | ,874^**^ | ,820^**^ |
| Day 9 (AM) | ,953^**^ | ,956^**^ | ,952^**^ | ,948^**^ | ,899^**^ | ,874^**^ |
| Day 9 (PM) | ,973^**^ | ,980^**^ | ,966^**^ | ,948^**^ | ,887^**^ | ,828^**^ |
| Day 10 (AM) | ,983^**^ | ,985^**^ | ,977^**^ | ,959^**^ | ,904^**^ | ,854^**^ |
| Day 10 (PM) | ,967^**^ | ,976^**^ | ,963^**^ | ,954^**^ | ,894^**^ | ,847^**^ |
| Day 11 (AM) | ,986^**^ | ,986^**^ | ,980^**^ | ,970^**^ | ,918^**^ | ,877^**^ |
| Day 11 (PM) | ,971^**^ | ,975^**^ | ,973^**^ | ,956^**^ | ,899^**^ | ,858^**^ |
| Day 12 (AM) | ,982^**^ | ,980^**^ | ,977^**^ | ,955^**^ | ,902^**^ | ,864^**^ |
| Day 12 (PM) | ,973^**^ | ,980^**^ | ,975^**^ | ,956^**^ | ,897^**^ | ,856^**^ |
| Day 13 (AM) | ,975^**^ | ,978^**^ | ,974^**^ | ,942^**^ | ,889^**^ | ,833^**^ |
| Day 13 (PM) | ,976^**^ | ,980^**^ | ,972^**^ | ,943^**^ | ,888^**^ | ,827^**^ |
| Day 14 (AM) | ,971^**^ | ,973^**^ | ,970^**^ | ,941^**^ | ,888^**^ | ,846^**^ |
| Day 14 (PM) | ,983^**^ | ,986^**^ | ,979^**^ | ,952^**^ | ,900^**^ | ,842^**^ |
| Day 15 (AM) | ,986^**^ | ,985^**^ | ,982^**^ | ,955^**^ | ,910^**^ | ,863^**^ |
| Day 15 (PM) | ,978^**^ | ,983^**^ | ,976^**^ | ,953^**^ | ,890^**^ | ,832^**^ |
| Day 16 (AM) | ,992^**^ | ,992^**^ | ,986^**^ | ,971^**^ | ,922^**^ | ,877^**^ |
| Day 16 (PM) | ,978^**^ | ,983^**^ | ,977^**^ | ,949^**^ | ,890^**^ | ,827^**^ |
| Day 17 (AM) | ,987^**^ | ,989^**^ | ,983^**^ | ,961^**^ | ,910^**^ | ,865^**^ |
| Day 17 (PM) | ,977^**^ | ,984^**^ | ,974^**^ | ,952^**^ | ,894^**^ | ,834^**^ |
| Day 18 (AM) | ,984^**^ | ,988^**^ | ,985^**^ | ,960^**^ | ,902^**^ | ,848^**^ |
| Day 18 (PM) | ,970^**^ | ,977^**^ | ,975^**^ | ,948^**^ | ,882^**^ | ,828^**^ |
| Day 19 (AM) | ,965^**^ | ,968^**^ | ,964^**^ | ,932^**^ | ,872^**^ | ,815^**^ |
| Day 19 (PM) | ,980^**^ | ,987^**^ | ,980^**^ | ,960^**^ | ,897^**^ | ,841^**^ |
| Day 20 (AM) | ,985^**^ | ,982^**^ | ,980^**^ | ,957^**^ | ,909^**^ | ,869^**^ |
| Day 20 (PM) | ,985^**^ | ,989^**^ | ,981^**^ | ,964^**^ | ,909^**^ | ,864^**^ |
| Day 21 (AM) | ,992^**^ | ,989^**^ | ,990^**^ | ,965^**^ | ,929^**^ | ,874^**^ |
| Day 21 (PM) | ,993^**^ | ,993^**^ | ,991^**^ | ,974^**^ | ,933^**^ | ,886^**^ |
| Day 22 (AM) | ,986^**^ | ,987^**^ | ,979^**^ | ,956^**^ | ,902^**^ | ,848^**^ |
| Day 22 (PM) | ,980^**^ | ,985^**^ | ,980^**^ | ,954^**^ | ,898^**^ | ,845^**^ |
| Day 23 (AM) | ,987^**^ | ,989^**^ | ,985^**^ | ,964^**^ | ,914^**^ | ,864^**^ |
| Day 23 (PM) | ,982^**^ | ,990^**^ | ,977^**^ | ,961^**^ | ,900^**^ | ,839^**^ |
| Day 24 (AM) | ,989^**^ | ,991^**^ | ,985^**^ | ,969^**^ | ,922^**^ | ,872^**^ |
| Day 24 (PM) | ,976^**^ | ,986^**^ | ,974^**^ | ,966^**^ | ,902^**^ | ,845^**^ |
| Day 25 (AM) | ,992^**^ | ,993^**^ | ,993^**^ | ,977^**^ | ,929^**^ | ,884^**^ |
| Day 25 (PM) | ,978^**^ | ,985^**^ | ,981^**^ | ,960^**^ | ,894^**^ | ,850^**^ |
| Day 26 (AM) | ,987^**^ | ,989^**^ | ,984^**^ | ,961^**^ | ,907^**^ | ,857^**^ |
| Day 26 (PM) | ,990^**^ | ,992^**^ | ,989^**^ | ,971^**^ | ,926^**^ | ,871^**^ |
| Day 27 (AM) | ,985^**^ | ,987^**^ | ,980^**^ | ,969^**^ | ,927^**^ | ,885^**^ |
| Day 27 (PM) | ,991^**^ | ,995^**^ | ,990^**^ | ,973^**^ | ,920^**^ | ,869^**^ |
| Day 28 (AM) | ,996^**^ | ,995^**^ | ,991^**^ | ,982^**^ | ,937^**^ | ,903^**^ |
| Day 28 (PM) | ,989^**^ | ,989^**^ | ,984^**^ | ,962^**^ | ,912^**^ | ,864^**^ |
| Day 29 (AM) | 1 | ,995^**^ | ,992^**^ | ,979^**^ | ,944^**^ | ,902^**^ |
| Day 29 (PM) |  | 1 | ,992^**^ | ,982^**^ | ,932^**^ | ,891^**^ |
| Day 30 (AM) |  |  | 1 | ,981^**^ | ,945^**^ | ,901^**^ |
| Day 30 (PM) |  |  |  | 1 | ,953^**^ | ,934^**^ |
| Day 31 (AM) |  |  |  |  | 1 | ,948^**^ |
| Day 31 (PM) |  |  |  |  |  | 1 |

**p<0.05, **p<0.001*

**S5 Table B.** Correlations for X from Day 1 (AM) to Day 31 (PM) (First-Level Agenda)

| **Days** | **Day 1** | | **Day 2** | | **Day 3** | | **Day 4** | | **Day 5** | | **Day 6** | | **Day 7** | |
| --- | --- | --- | --- | --- | --- | --- | --- | --- | --- | --- | --- | --- | --- | --- |
|  | **AM** | **PM** | **AM** | **PM** | **AM** | **PM** | **AM** | **PM** | **AM** | **PM** | **AM** | **PM** | **AM** | **PM** |
| Day 1 (AM) | 1 | ,990^**^ | ,988^**^ | ,986^**^ | ,990^**^ | ,981^**^ | ,994^**^ | ,991^**^ | ,987^**^ | ,993^**^ | ,990^**^ | ,988^**^ | ,994^**^ | ,975^**^ |
| Day 1 (PM) |  | 1 | ,989^**^ | ,993^**^ | ,992^**^ | ,991^**^ | ,993^**^ | ,990^**^ | ,979^**^ | ,996^**^ | ,993^**^ | ,993^**^ | ,989^**^ | ,984^**^ |
| Day 2 (AM) |  |  | 1 | ,990^**^ | ,991^**^ | ,980^**^ | ,988^**^ | ,985^**^ | ,981^**^ | ,990^**^ | ,986^**^ | ,982^**^ | ,985^**^ | ,970^**^ |
| Day 2 (PM) |  |  |  | 1 | ,996^**^ | ,994^**^ | ,990^**^ | ,987^**^ | ,983^**^ | ,993^**^ | ,991^**^ | ,990^**^ | ,985^**^ | ,985^**^ |
| Day 3 (AM) |  |  |  |  | 1 | ,991^**^ | ,989^**^ | ,985^**^ | ,989^**^ | ,992^**^ | ,994^**^ | ,986^**^ | ,987^**^ | ,979^**^ |
| Day 3 (PM) |  |  |  |  |  | 1 | ,987^**^ | ,982^**^ | ,973^**^ | ,991^**^ | ,991^**^ | ,991^**^ | ,981^**^ | ,991^**^ |
| Day 4 (AM) |  |  |  |  |  |  | 1 | ,994^**^ | ,982^**^ | ,995^**^ | ,990^**^ | ,993^**^ | ,994^**^ | ,982^**^ |
| Day 4 (PM) |  |  |  |  |  |  |  | 1 | ,982^**^ | ,991^**^ | ,984^**^ | ,990^**^ | ,988^**^ | ,977^**^ |
| Day 5 (AM) |  |  |  |  |  |  |  |  | 1 | ,982^**^ | ,984^**^ | ,973^**^ | ,983^**^ | ,963^**^ |
| Day 5 (PM) |  |  |  |  |  |  |  |  |  | 1 | ,994^**^ | ,995^**^ | ,992^**^ | ,985^**^ |
| Day 6 (AM) |  |  |  |  |  |  |  |  |  |  | 1 | ,991^**^ | ,990^**^ | ,983^**^ |
| Day 6 (PM) |  |  |  |  |  |  |  |  |  |  |  | 1 | ,990^**^ | ,990^**^ |
| Day 7 (AM) |  |  |  |  |  |  |  |  |  |  |  |  | 1 | ,978^**^ |
| Day 7 (PM) |  |  |  |  |  |  |  |  |  |  |  |  |  | 1 |

**p<0.05, **p<0.001*

**S5 Table B.** Correlations for X from Day 1 (AM) to Day 31 (PM) (First-Level Agenda) – **Continued**

| **Days** | **Day 8** | | **Day 9** | | **Day 10** | | **Day 11** | | **Day 12** | | **Day 13** | | **Day 14** | |
| --- | --- | --- | --- | --- | --- | --- | --- | --- | --- | --- | --- | --- | --- | --- |
|  | **AM** | **PM** | **AM** | **PM** | **AM** | **PM** | **AM** | **PM** | **AM** | **PM** | **AM** | **PM** | **AM** | **PM** |
| Day 1 (AM) | ,953^**^ | ,953^**^ | ,956^**^ | ,941^**^ | ,962^**^ | ,931^**^ | ,916^**^ | ,934^**^ | ,960^**^ | ,944^**^ | ,939^**^ | ,951^**^ | ,948^**^ | ,942^**^ |
| Day 1 (PM) | ,954^**^ | ,962^**^ | ,964^**^ | ,954^**^ | ,973^**^ | ,946^**^ | ,928^**^ | ,940^**^ | ,966^**^ | ,955^**^ | ,945^**^ | ,957^**^ | ,951^**^ | ,951^**^ |
| Day 2 (AM) | ,947^**^ | ,951^**^ | ,955^**^ | ,942^**^ | ,961^**^ | ,932^**^ | ,926^**^ | ,931^**^ | ,958^**^ | ,944^**^ | ,940^**^ | ,946^**^ | ,943^**^ | ,940^**^ |
| Day 2 (PM) | ,967^**^ | ,972^**^ | ,971^**^ | ,964^**^ | ,978^**^ | ,958^**^ | ,944^**^ | ,952^**^ | ,974^**^ | ,966^**^ | ,958^**^ | ,966^**^ | ,961^**^ | ,961^**^ |
| Day 3 (AM) | ,967^**^ | ,970^**^ | ,971^**^ | ,963^**^ | ,978^**^ | ,958^**^ | ,949^**^ | ,950^**^ | ,976^**^ | ,967^**^ | ,962^**^ | ,965^**^ | ,965^**^ | ,962^**^ |
| Day 3 (PM) | ,967^**^ | ,975^**^ | ,971^**^ | ,967^**^ | ,982^**^ | ,963^**^ | ,943^**^ | ,955^**^ | ,975^**^ | ,971^**^ | ,957^**^ | ,970^**^ | ,961^**^ | ,964^**^ |
| Day 4 (AM) | ,953^**^ | ,958^**^ | ,960^**^ | ,947^**^ | ,966^**^ | ,935^**^ | ,918^**^ | ,934^**^ | ,960^**^ | ,947^**^ | ,938^**^ | ,952^**^ | ,945^**^ | ,943^**^ |
| Day 4 (PM) | ,941^**^ | ,947^**^ | ,952^**^ | ,940^**^ | ,957^**^ | ,930^**^ | ,910^**^ | ,923^**^ | ,949^**^ | ,941^**^ | ,933^**^ | ,943^**^ | ,937^**^ | ,939^**^ |
| Day 5 (AM) | ,963^**^ | ,958^**^ | ,958^**^ | ,943^**^ | ,960^**^ | ,936^**^ | ,933^**^ | ,932^**^ | ,962^**^ | ,950^**^ | ,950^**^ | ,948^**^ | ,956^**^ | ,946^**^ |
| Day 5 (PM) | ,959^**^ | ,963^**^ | ,962^**^ | ,950^**^ | ,971^**^ | ,942^**^ | ,923^**^ | ,944^**^ | ,966^**^ | ,956^**^ | ,944^**^ | ,959^**^ | ,951^**^ | ,949^**^ |
| Day 6 (AM) | ,969^**^ | ,971^**^ | ,970^**^ | ,961^**^ | ,979^**^ | ,955^**^ | ,940^**^ | ,952^**^ | ,977^**^ | ,967^**^ | ,959^**^ | ,968^**^ | ,965^**^ | ,962^**^ |
| Day 6 (PM) | ,955^**^ | ,963^**^ | ,964^**^ | ,956^**^ | ,973^**^ | ,947^**^ | ,922^**^ | ,943^**^ | ,964^**^ | ,958^**^ | ,945^**^ | ,960^**^ | ,949^**^ | ,952^**^ |
| Day 7 (AM) | ,955^**^ | ,955^**^ | ,958^**^ | ,942^**^ | ,964^**^ | ,930^**^ | ,917^**^ | ,932^**^ | ,960^**^ | ,944^**^ | ,938^**^ | ,949^**^ | ,948^**^ | ,942^**^ |
| Day 7 (PM) | ,970^**^ | ,978^**^ | ,974^**^ | ,971^**^ | ,983^**^ | ,964^**^ | ,938^**^ | ,956^**^ | ,973^**^ | ,974^**^ | ,958^**^ | ,976^**^ | ,963^**^ | ,970^**^ |
| Day 8 (AM) | 1 | ,994^**^ | ,983^**^ | ,978^**^ | ,985^**^ | ,971^**^ | ,968^**^ | ,969^**^ | ,985^**^ | ,981^**^ | ,975^**^ | ,980^**^ | ,984^**^ | ,980^**^ |
| Day 8 (PM) |  | 1 | ,993^**^ | ,990^**^ | ,993^**^ | ,984^**^ | ,975^**^ | ,969^**^ | ,987^**^ | ,987^**^ | ,981^**^ | ,981^**^ | ,983^**^ | ,985^**^ |
| Day 9 (AM) |  |  | 1 | ,994^**^ | ,994^**^ | ,987^**^ | ,978^**^ | ,964^**^ | ,986^**^ | ,983^**^ | ,983^**^ | ,977^**^ | ,983^**^ | ,985^**^ |
| Day 9 (PM) |  |  |  | 1 | ,992^**^ | ,995^**^ | ,981^**^ | ,963^**^ | ,984^**^ | ,988^**^ | ,986^**^ | ,978^**^ | ,981^**^ | ,989^**^ |
| Day 10 (AM) |  |  |  |  | 1 | ,987^**^ | ,972^**^ | ,970^**^ | ,991^**^ | ,987^**^ | ,980^**^ | ,983^**^ | ,984^**^ | ,986^**^ |
| Day 10 (PM) |  |  |  |  |  | 1 | ,982^**^ | ,967^**^ | ,981^**^ | ,991^**^ | ,988^**^ | ,980^**^ | ,980^**^ | ,989^**^ |
| Day 11 (AM) |  |  |  |  |  |  | 1 | ,957^**^ | ,976^**^ | ,976^**^ | ,984^**^ | ,966^**^ | ,981^**^ | ,980^**^ |
| Day 11 (PM) |  |  |  |  |  |  |  | 1 | ,982^**^ | ,978^**^ | ,965^**^ | ,991^**^ | ,980^**^ | ,975^**^ |
| Day 12 (AM) |  |  |  |  |  |  |  |  | 1 | ,989^**^ | ,984^**^ | ,990^**^ | ,992^**^ | ,988^**^ |
| Day 12 (PM) |  |  |  |  |  |  |  |  |  | 1 | ,993^**^ | ,991^**^ | ,987^**^ | ,991^**^ |
| Day 13 (AM) |  |  |  |  |  |  |  |  |  |  | 1 | ,981^**^ | ,984^**^ | ,986^**^ |
| Day 13 (PM) |  |  |  |  |  |  |  |  |  |  |  | 1 | ,988^**^ | ,989^**^ |
| Day 14 (AM) |  |  |  |  |  |  |  |  |  |  |  |  | 1 | ,993^**^ |
| Day 14 (PM) |  |  |  |  |  |  |  |  |  |  |  |  |  | 1 |

**p<0.05, **p<0.001*

**S5 Table B.** Correlations for X from Day 1 (AM) to Day 31 (PM) (First-Level Agenda) – **Continued**

| **Days** | **Day 15** | | **Day 16** | | **Day 17** | | **Day 18** | | **Day 19** | | **Day 20** | | **Day 21** | |
| --- | --- | --- | --- | --- | --- | --- | --- | --- | --- | --- | --- | --- | --- | --- |
|  | **AM** | **PM** | **AM** | **PM** | **AM** | **PM** | **AM** | **PM** | **AM** | **PM** | **AM** | **PM** | **AM** | **PM** |
| Day 1 (AM) | ,937^**^ | ,953^**^ | ,958^**^ | ,964^**^ | ,960^**^ | ,942^**^ | ,966^**^ | ,949^**^ | ,958^**^ | ,952^**^ | ,972^**^ | ,962^**^ | ,962^**^ | ,952^**^ |
| Day 1 (PM) | ,942^**^ | ,961^**^ | ,962^**^ | ,971^**^ | ,963^**^ | ,943^**^ | ,969^**^ | ,958^**^ | ,963^**^ | ,960^**^ | ,976^**^ | ,969^**^ | ,968^**^ | ,964^**^ |
| Day 2 (AM) | ,935^**^ | ,952^**^ | ,956^**^ | ,961^**^ | ,956^**^ | ,933^**^ | ,965^**^ | ,948^**^ | ,955^**^ | ,949^**^ | ,969^**^ | ,961^**^ | ,960^**^ | ,951^**^ |
| Day 2 (PM) | ,954^**^ | ,973^**^ | ,974^**^ | ,981^**^ | ,974^**^ | ,950^**^ | ,978^**^ | ,970^**^ | ,973^**^ | ,969^**^ | ,982^**^ | ,977^**^ | ,976^**^ | ,972^**^ |
| Day 3 (AM) | ,957^**^ | ,973^**^ | ,975^**^ | ,978^**^ | ,975^**^ | ,948^**^ | ,979^**^ | ,970^**^ | ,974^**^ | ,969^**^ | ,984^**^ | ,977^**^ | ,978^**^ | ,971^**^ |
| Day 3 (PM) | ,956^**^ | ,975^**^ | ,974^**^ | ,983^**^ | ,974^**^ | ,954^**^ | ,977^**^ | ,972^**^ | ,972^**^ | ,973^**^ | ,982^**^ | ,978^**^ | ,976^**^ | ,977^**^ |
| Day 4 (AM) | ,938^**^ | ,956^**^ | ,960^**^ | ,966^**^ | ,960^**^ | ,939^**^ | ,967^**^ | ,952^**^ | ,960^**^ | ,954^**^ | ,973^**^ | ,966^**^ | ,963^**^ | ,958^**^ |
| Day 4 (PM) | ,927^**^ | ,949^**^ | ,952^**^ | ,960^**^ | ,951^**^ | ,930^**^ | ,955^**^ | ,947^**^ | ,952^**^ | ,945^**^ | ,968^**^ | ,959^**^ | ,955^**^ | ,950^**^ |
| Day 5 (AM) | ,948^**^ | ,960^**^ | ,969^**^ | ,965^**^ | ,964^**^ | ,936^**^ | ,970^**^ | ,954^**^ | ,962^**^ | ,952^**^ | ,974^**^ | ,962^**^ | ,968^**^ | ,953^**^ |
| Day 5 (PM) | ,944^**^ | ,961^**^ | ,963^**^ | ,973^**^ | ,964^**^ | ,946^**^ | ,972^**^ | ,956^**^ | ,963^**^ | ,959^**^ | ,976^**^ | ,968^**^ | ,967^**^ | ,963^**^ |
| Day 6 (AM) | ,958^**^ | ,972^**^ | ,974^**^ | ,979^**^ | ,976^**^ | ,954^**^ | ,980^**^ | ,968^**^ | ,974^**^ | ,971^**^ | ,984^**^ | ,976^**^ | ,978^**^ | ,972^**^ |
| Day 6 (PM) | ,940^**^ | ,961^**^ | ,962^**^ | ,974^**^ | ,965^**^ | ,949^**^ | ,967^**^ | ,960^**^ | ,964^**^ | ,962^**^ | ,976^**^ | ,971^**^ | ,967^**^ | ,966^**^ |
| Day 7 (AM) | ,938^**^ | ,953^**^ | ,958^**^ | ,963^**^ | ,961^**^ | ,946^**^ | ,968^**^ | ,948^**^ | ,959^**^ | ,951^**^ | ,971^**^ | ,963^**^ | ,963^**^ | ,955^**^ |
| Day 7 (PM) | ,961^**^ | ,977^**^ | ,975^**^ | ,985^**^ | ,975^**^ | ,964^**^ | ,972^**^ | ,976^**^ | ,976^**^ | ,976^**^ | ,982^**^ | ,980^**^ | ,975^**^ | ,979^**^ |
| Day 8 (AM) | ,985^**^ | ,988^**^ | ,991^**^ | ,986^**^ | ,990^**^ | ,969^**^ | ,987^**^ | ,981^**^ | ,986^**^ | ,982^**^ | ,983^**^ | ,982^**^ | ,987^**^ | ,981^**^ |
| Day 8 (PM) | ,982^**^ | ,993^**^ | ,991^**^ | ,990^**^ | ,990^**^ | ,965^**^ | ,985^**^ | ,989^**^ | ,990^**^ | ,988^**^ | ,988^**^ | ,989^**^ | ,990^**^ | ,990^**^ |
| Day 9 (AM) | ,977^**^ | ,990^**^ | ,988^**^ | ,986^**^ | ,989^**^ | ,960^**^ | ,981^**^ | ,990^**^ | ,991^**^ | ,987^**^ | ,990^**^ | ,992^**^ | ,991^**^ | ,990^**^ |
| Day 9 (PM) | ,973^**^ | ,990^**^ | ,984^**^ | ,985^**^ | ,986^**^ | ,955^**^ | ,971^**^ | ,993^**^ | ,989^**^ | ,988^**^ | ,986^**^ | ,991^**^ | ,988^**^ | ,991^**^ |
| Day 10 (AM) | ,979^**^ | ,990^**^ | ,988^**^ | ,989^**^ | ,990^**^ | ,967^**^ | ,985^**^ | ,989^**^ | ,991^**^ | ,990^**^ | ,992^**^ | ,992^**^ | ,992^**^ | ,993^**^ |
| Day 10 (PM) | ,971^**^ | ,989^**^ | ,980^**^ | ,982^**^ | ,981^**^ | ,949^**^ | ,967^**^ | ,994^**^ | ,986^**^ | ,988^**^ | ,983^**^ | ,987^**^ | ,984^**^ | ,988^**^ |
| Day 11 (AM) | ,970^**^ | ,981^**^ | ,976^**^ | ,966^**^ | ,975^**^ | ,937^**^ | ,963^**^ | ,980^**^ | ,978^**^ | ,975^**^ | ,974^**^ | ,976^**^ | ,979^**^ | ,975^**^ |
| Day 11 (PM) | ,967^**^ | ,975^**^ | ,971^**^ | ,975^**^ | ,977^**^ | ,961^**^ | ,975^**^ | ,974^**^ | ,978^**^ | ,985^**^ | ,974^**^ | ,973^**^ | ,975^**^ | ,972^**^ |
| Day 12 (AM) | ,983^**^ | ,990^**^ | ,988^**^ | ,985^**^ | ,992^**^ | ,969^**^ | ,988^**^ | ,986^**^ | ,992^**^ | ,992^**^ | ,991^**^ | ,990^**^ | ,993^**^ | ,987^**^ |
| Day 12 (PM) | ,981^**^ | ,993^**^ | ,988^**^ | ,989^**^ | ,987^**^ | ,961^**^ | ,977^**^ | ,993^**^ | ,989^**^ | ,991^**^ | ,988^**^ | ,988^**^ | ,989^**^ | ,989^**^ |
| Day 13 (AM) | ,976^**^ | ,989^**^ | ,984^**^ | ,979^**^ | ,983^**^ | ,948^**^ | ,970^**^ | ,988^**^ | ,986^**^ | ,983^**^ | ,984^**^ | ,985^**^ | ,986^**^ | ,982^**^ |
| Day 13 (PM) | ,981^**^ | ,989^**^ | ,984^**^ | ,986^**^ | ,987^**^ | ,971^**^ | ,980^**^ | ,987^**^ | ,990^**^ | ,992^**^ | ,986^**^ | ,985^**^ | ,985^**^ | ,983^**^ |
| Day 14 (AM) | ,989^**^ | ,991^**^ | ,989^**^ | ,982^**^ | ,991^**^ | ,971^**^ | ,983^**^ | ,987^**^ | ,993^**^ | ,990^**^ | ,989^**^ | ,987^**^ | ,992^**^ | ,983^**^ |
| Day 14 (PM) | ,989^**^ | ,995^**^ | ,987^**^ | ,985^**^ | ,989^**^ | ,971^**^ | ,976^**^ | ,994^**^ | ,994^**^ | ,991^**^ | ,989^**^ | ,988^**^ | ,989^**^ | ,988^**^ |
| Day 15 (AM) | 1 | ,991^**^ | ,989^**^ | ,980^**^ | ,984^**^ | ,967^**^ | ,981^**^ | ,983^**^ | ,988^**^ | ,982^**^ | ,981^**^ | ,976^**^ | ,983^**^ | ,977^**^ |
| Day 15 (PM) |  | 1 | ,995^**^ | ,993^**^ | ,992^**^ | ,969^**^ | ,985^**^ | ,996^**^ | ,995^**^ | ,992^**^ | ,993^**^ | ,992^**^ | ,993^**^ | ,991^**^ |
| Day 16 (AM) |  |  | 1 | ,993^**^ | ,993^**^ | ,967^**^ | ,989^**^ | ,991^**^ | ,993^**^ | ,988^**^ | ,990^**^ | ,989^**^ | ,993^**^ | ,988^**^ |
| Day 16 (PM) |  |  |  | 1 | ,991^**^ | ,968^**^ | ,987^**^ | ,992^**^ | ,989^**^ | ,989^**^ | ,990^**^ | ,990^**^ | ,989^**^ | ,989^**^ |
| Day 17 (AM) |  |  |  |  | 1 | ,976^**^ | ,989^**^ | ,990^**^ | ,994^**^ | ,992^**^ | ,992^**^ | ,991^**^ | ,995^**^ | ,990^**^ |
| Day 17 (PM) |  |  |  |  |  | 1 | ,971^**^ | ,964^**^ | ,968^**^ | ,971^**^ | ,967^**^ | ,965^**^ | ,968^**^ | ,966^**^ |
| Day 18 (AM) |  |  |  |  |  |  | 1 | ,980^**^ | ,986^**^ | ,985^**^ | ,988^**^ | ,986^**^ | ,991^**^ | ,983^**^ |
| Day 18 (PM) |  |  |  |  |  |  |  | 1 | ,995^**^ | ,994^**^ | ,991^**^ | ,992^**^ | ,991^**^ | ,991^**^ |
| Day 19 (AM) |  |  |  |  |  |  |  |  | 1 | ,994^**^ | ,994^**^ | ,993^**^ | ,994^**^ | ,990^**^ |
| Day 19 (PM) |  |  |  |  |  |  |  |  |  | 1 | ,993^**^ | ,993^**^ | ,993^**^ | ,991^**^ |
| Day 20 (AM) |  |  |  |  |  |  |  |  |  |  | 1 | ,996^**^ | ,996^**^ | ,991^**^ |
| Day 20 (PM) |  |  |  |  |  |  |  |  |  |  |  | 1 | ,997^**^ | ,993^**^ |
| Day 21 (AM) |  |  |  |  |  |  |  |  |  |  |  |  | 1 | ,994^**^ |
| Day 21 (PM) |  |  |  |  |  |  |  |  |  |  |  |  |  | 1 |

**p<0.05, **p<0.001*

**S5 Table B.** Correlations for X from Day 1 (AM) to Day 31 (PM) (First-Level Agenda) – **Continued**

| **Days** | **Day 22** | | **Day 23** | | **Day 24** | | **Day 25** | | **Day 26** | | **Day 27** | | **Day 28** | |
| --- | --- | --- | --- | --- | --- | --- | --- | --- | --- | --- | --- | --- | --- | --- |
|  | **AM** | **PM** | **AM** | **PM** | **AM** | **PM** | **AM** | **PM** | **AM** | **PM** | **AM** | **PM** | **AM** | **PM** |
| Day 1 (AM) | ,959^**^ | ,926^**^ | ,933^**^ | ,959^**^ | ,967^**^ | ,941^**^ | ,962^**^ | ,950^**^ | ,954^**^ | ,957^**^ | ,960^**^ | ,947^**^ | ,952^**^ | ,944^**^ |
| Day 1 (PM) | ,961^**^ | ,936^**^ | ,938^**^ | ,963^**^ | ,972^**^ | ,947^**^ | ,967^**^ | ,957^**^ | ,956^**^ | ,966^**^ | ,970^**^ | ,957^**^ | ,959^**^ | ,955^**^ |
| Day 2 (AM) | ,955^**^ | ,925^**^ | ,933^**^ | ,956^**^ | ,965^**^ | ,939^**^ | ,960^**^ | ,949^**^ | ,951^**^ | ,956^**^ | ,960^**^ | ,947^**^ | ,952^**^ | ,945^**^ |
| Day 2 (PM) | ,972^**^ | ,948^**^ | ,950^**^ | ,974^**^ | ,980^**^ | ,960^**^ | ,977^**^ | ,968^**^ | ,968^**^ | ,972^**^ | ,975^**^ | ,965^**^ | ,968^**^ | ,966^**^ |
| Day 3 (AM) | ,975^**^ | ,949^**^ | ,954^**^ | ,974^**^ | ,980^**^ | ,961^**^ | ,978^**^ | ,970^**^ | ,971^**^ | ,972^**^ | ,976^**^ | ,967^**^ | ,970^**^ | ,966^**^ |
| Day 3 (PM) | ,972^**^ | ,950^**^ | ,946^**^ | ,973^**^ | ,981^**^ | ,961^**^ | ,978^**^ | ,969^**^ | ,967^**^ | ,973^**^ | ,978^**^ | ,968^**^ | ,969^**^ | ,970^**^ |
| Day 4 (AM) | ,959^**^ | ,929^**^ | ,933^**^ | ,960^**^ | ,970^**^ | ,943^**^ | ,965^**^ | ,951^**^ | ,954^**^ | ,961^**^ | ,965^**^ | ,952^**^ | ,956^**^ | ,950^**^ |
| Day 4 (PM) | ,949^**^ | ,924^**^ | ,927^**^ | ,954^**^ | ,960^**^ | ,938^**^ | ,957^**^ | ,944^**^ | ,943^**^ | ,952^**^ | ,955^**^ | ,941^**^ | ,944^**^ | ,941^**^ |
| Day 5 (AM) | ,965^**^ | ,935^**^ | ,945^**^ | ,964^**^ | ,969^**^ | ,945^**^ | ,965^**^ | ,957^**^ | ,961^**^ | ,956^**^ | ,961^**^ | ,951^**^ | ,958^**^ | ,950^**^ |
| Day 5 (PM) | ,963^**^ | ,935^**^ | ,936^**^ | ,965^**^ | ,974^**^ | ,947^**^ | ,968^**^ | ,957^**^ | ,959^**^ | ,966^**^ | ,970^**^ | ,956^**^ | ,959^**^ | ,955^**^ |
| Day 6 (AM) | ,974^**^ | ,946^**^ | ,949^**^ | ,973^**^ | ,981^**^ | ,956^**^ | ,977^**^ | ,968^**^ | ,971^**^ | ,973^**^ | ,978^**^ | ,967^**^ | ,970^**^ | ,965^**^ |
| Day 6 (PM) | ,961^**^ | ,939^**^ | ,935^**^ | ,965^**^ | ,973^**^ | ,950^**^ | ,970^**^ | ,957^**^ | ,957^**^ | ,967^**^ | ,969^**^ | ,957^**^ | ,958^**^ | ,957^**^ |
| Day 7 (AM) | ,961^**^ | ,929^**^ | ,935^**^ | ,961^**^ | ,970^**^ | ,941^**^ | ,964^**^ | ,951^**^ | ,956^**^ | ,960^**^ | ,963^**^ | ,951^**^ | ,957^**^ | ,948^**^ |
| Day 7 (PM) | ,972^**^ | ,955^**^ | ,948^**^ | ,973^**^ | ,979^**^ | ,959^**^ | ,977^**^ | ,969^**^ | ,968^**^ | ,976^**^ | ,980^**^ | ,970^**^ | ,968^**^ | ,973^**^ |
| Day 8 (AM) | ,988^**^ | ,967^**^ | ,970^**^ | ,980^**^ | ,983^**^ | ,959^**^ | ,981^**^ | ,982^**^ | ,989^**^ | ,979^**^ | ,984^**^ | ,982^**^ | ,985^**^ | ,982^**^ |
| Day 8 (PM) | ,989^**^ | ,976^**^ | ,975^**^ | ,984^**^ | ,985^**^ | ,968^**^ | ,986^**^ | ,989^**^ | ,989^**^ | ,986^**^ | ,989^**^ | ,990^**^ | ,989^**^ | ,991^**^ |
| Day 9 (AM) | ,989^**^ | ,980^**^ | ,984^**^ | ,987^**^ | ,986^**^ | ,974^**^ | ,988^**^ | ,992^**^ | ,989^**^ | ,990^**^ | ,990^**^ | ,993^**^ | ,991^**^ | ,992^**^ |
| Day 9 (PM) | ,986^**^ | ,987^**^ | ,986^**^ | ,985^**^ | ,980^**^ | ,974^**^ | ,986^**^ | ,992^**^ | ,984^**^ | ,987^**^ | ,986^**^ | ,992^**^ | ,987^**^ | ,994^**^ |
| Day 10 (AM) | ,990^**^ | ,975^**^ | ,976^**^ | ,987^**^ | ,991^**^ | ,974^**^ | ,990^**^ | ,990^**^ | ,988^**^ | ,992^**^ | ,994^**^ | ,992^**^ | ,990^**^ | ,991^**^ |
| Day 10 (PM) | ,982^**^ | ,987^**^ | ,981^**^ | ,981^**^ | ,975^**^ | ,974^**^ | ,982^**^ | ,990^**^ | ,979^**^ | ,981^**^ | ,981^**^ | ,987^**^ | ,981^**^ | ,991^**^ |
| Day 11 (AM) | ,978^**^ | ,978^**^ | ,983^**^ | ,972^**^ | ,967^**^ | ,957^**^ | ,971^**^ | ,981^**^ | ,979^**^ | ,972^**^ | ,972^**^ | ,981^**^ | ,979^**^ | ,980^**^ |
| Day 11 (PM) | ,974^**^ | ,954^**^ | ,951^**^ | ,964^**^ | ,970^**^ | ,944^**^ | ,965^**^ | ,966^**^ | ,973^**^ | ,971^**^ | ,972^**^ | ,969^**^ | ,970^**^ | ,969^**^ |
| Day 12 (AM) | ,991^**^ | ,972^**^ | ,976^**^ | ,983^**^ | ,988^**^ | ,964^**^ | ,985^**^ | ,987^**^ | ,991^**^ | ,991^**^ | ,992^**^ | ,990^**^ | ,990^**^ | ,986^**^ |
| Day 12 (PM) | ,986^**^ | ,985^**^ | ,978^**^ | ,982^**^ | ,981^**^ | ,968^**^ | ,985^**^ | ,990^**^ | ,986^**^ | ,986^**^ | ,986^**^ | ,986^**^ | ,983^**^ | ,991^**^ |
| Day 13 (AM) | ,984^**^ | ,989^**^ | ,987^**^ | ,980^**^ | ,974^**^ | ,964^**^ | ,980^**^ | ,990^**^ | ,985^**^ | ,981^**^ | ,980^**^ | ,985^**^ | ,982^**^ | ,987^**^ |
| Day 13 (PM) | ,984^**^ | ,970^**^ | ,966^**^ | ,976^**^ | ,980^**^ | ,958^**^ | ,979^**^ | ,980^**^ | ,983^**^ | ,983^**^ | ,984^**^ | ,980^**^ | ,978^**^ | ,981^**^ |
| Day 14 (AM) | ,988^**^ | ,973^**^ | ,979^**^ | ,981^**^ | ,982^**^ | ,961^**^ | ,982^**^ | ,985^**^ | ,989^**^ | ,985^**^ | ,986^**^ | ,987^**^ | ,987^**^ | ,985^**^ |
| Day 14 (PM) | ,985^**^ | ,979^**^ | ,978^**^ | ,980^**^ | ,978^**^ | ,963^**^ | ,982^**^ | ,987^**^ | ,985^**^ | ,985^**^ | ,986^**^ | ,987^**^ | ,983^**^ | ,988^**^ |
| Day 15 (AM) | ,981^**^ | ,962^**^ | ,966^**^ | ,972^**^ | ,975^**^ | ,953^**^ | ,975^**^ | ,979^**^ | ,985^**^ | ,977^**^ | ,981^**^ | ,981^**^ | ,980^**^ | ,980^**^ |
| Day 15 (PM) | ,991^**^ | ,981^**^ | ,981^**^ | ,987^**^ | ,986^**^ | ,972^**^ | ,989^**^ | ,992^**^ | ,991^**^ | ,987^**^ | ,990^**^ | ,991^**^ | ,988^**^ | ,993^**^ |
| Day 16 (AM) | ,991^**^ | ,976^**^ | ,979^**^ | ,988^**^ | ,988^**^ | ,972^**^ | ,989^**^ | ,989^**^ | ,992^**^ | ,985^**^ | ,989^**^ | ,988^**^ | ,989^**^ | ,989^**^ |
| Day 16 (PM) | ,988^**^ | ,975^**^ | ,971^**^ | ,989^**^ | ,988^**^ | ,976^**^ | ,990^**^ | ,988^**^ | ,986^**^ | ,984^**^ | ,988^**^ | ,985^**^ | ,983^**^ | ,988^**^ |
| Day 17 (AM) | ,993^**^ | ,977^**^ | ,980^**^ | ,989^**^ | ,990^**^ | ,970^**^ | ,989^**^ | ,990^**^ | ,993^**^ | ,991^**^ | ,991^**^ | ,991^**^ | ,991^**^ | ,990^**^ |
| Day 17 (PM) | ,965^**^ | ,942^**^ | ,941^**^ | ,960^**^ | ,965^**^ | ,939^**^ | ,963^**^ | ,959^**^ | ,966^**^ | ,965^**^ | ,968^**^ | ,961^**^ | ,961^**^ | ,959^**^ |
| Day 18 (AM) | ,988^**^ | ,961^**^ | ,965^**^ | ,981^**^ | ,989^**^ | ,962^**^ | ,984^**^ | ,981^**^ | ,989^**^ | ,984^**^ | ,988^**^ | ,983^**^ | ,987^**^ | ,981^**^ |
| Day 18 (PM) | ,989^**^ | ,986^**^ | ,982^**^ | ,987^**^ | ,984^**^ | ,976^**^ | ,989^**^ | ,993^**^ | ,987^**^ | ,986^**^ | ,987^**^ | ,989^**^ | ,985^**^ | ,993^**^ |
| Day 19 (AM) | ,993^**^ | ,979^**^ | ,982^**^ | ,987^**^ | ,988^**^ | ,971^**^ | ,989^**^ | ,991^**^ | ,992^**^ | ,990^**^ | ,991^**^ | ,991^**^ | ,990^**^ | ,991^**^ |
| Day 19 (PM) | ,990^**^ | ,978^**^ | ,976^**^ | ,984^**^ | ,986^**^ | ,968^**^ | ,986^**^ | ,988^**^ | ,988^**^ | ,988^**^ | ,989^**^ | ,989^**^ | ,987^**^ | ,989^**^ |
| Day 20 (AM) | ,992^**^ | ,976^**^ | ,979^**^ | ,988^**^ | ,990^**^ | ,970^**^ | ,990^**^ | ,990^**^ | ,990^**^ | ,991^**^ | ,992^**^ | ,990^**^ | ,990^**^ | ,989^**^ |
| Day 20 (PM) | ,991^**^ | ,981^**^ | ,982^**^ | ,988^**^ | ,988^**^ | ,971^**^ | ,989^**^ | ,991^**^ | ,990^**^ | ,992^**^ | ,991^**^ | ,992^**^ | ,991^**^ | ,992^**^ |
| Day 21 (AM) | ,994^**^ | ,980^**^ | ,983^**^ | ,989^**^ | ,990^**^ | ,971^**^ | ,991^**^ | ,992^**^ | ,994^**^ | ,992^**^ | ,993^**^ | ,993^**^ | ,994^**^ | ,992^**^ |
| Day 21 (PM) | ,992^**^ | ,982^**^ | ,979^**^ | ,987^**^ | ,988^**^ | ,972^**^ | ,989^**^ | ,991^**^ | ,989^**^ | ,991^**^ | ,992^**^ | ,993^**^ | ,991^**^ | ,995^**^ |
| Day 22 (AM) | 1 | ,982^**^ | ,986^**^ | ,993^**^ | ,993^**^ | ,975^**^ | ,992^**^ | ,993^**^ | ,996^**^ | ,991^**^ | ,993^**^ | ,994^**^ | ,994^**^ | ,992^**^ |
| Day 22 (PM) |  | 1 | ,990^**^ | ,983^**^ | ,973^**^ | ,971^**^ | ,981^**^ | ,989^**^ | ,981^**^ | ,978^**^ | ,977^**^ | ,984^**^ | ,979^**^ | ,989^**^ |
| Day 23 (AM) |  |  | 1 | ,987^**^ | ,977^**^ | ,970^**^ | ,981^**^ | ,989^**^ | ,985^**^ | ,980^**^ | ,980^**^ | ,987^**^ | ,985^**^ | ,987^**^ |
| Day 23 (PM) |  |  |  | 1 | ,995^**^ | ,989^**^ | ,996^**^ | ,993^**^ | ,990^**^ | ,988^**^ | ,991^**^ | ,990^**^ | ,990^**^ | ,990^**^ |
| Day 24 (AM) |  |  |  |  | 1 | ,983^**^ | ,996^**^ | ,989^**^ | ,991^**^ | ,991^**^ | ,995^**^ | ,991^**^ | ,992^**^ | ,988^**^ |
| Day 24 (PM) |  |  |  |  |  | 1 | ,991^**^ | ,983^**^ | ,972^**^ | ,969^**^ | ,974^**^ | ,975^**^ | ,970^**^ | ,976^**^ |
| Day 25 (AM) |  |  |  |  |  |  | 1 | ,995^**^ | ,991^**^ | ,990^**^ | ,992^**^ | ,992^**^ | ,990^**^ | ,991^**^ |
| Day 25 (PM) |  |  |  |  |  |  |  | 1 | ,994^**^ | ,992^**^ | ,992^**^ | ,996^**^ | ,993^**^ | ,996^**^ |
| Day 26 (AM) |  |  |  |  |  |  |  |  | 1 | ,993^**^ | ,994^**^ | ,995^**^ | ,996^**^ | ,992^**^ |
| Day 26 (PM) |  |  |  |  |  |  |  |  |  | 1 | ,997^**^ | ,996^**^ | ,995^**^ | ,992^**^ |
| Day 27 (AM) |  |  |  |  |  |  |  |  |  |  | 1 | ,996^**^ | ,995^**^ | ,993^**^ |
| Day 27 (PM) |  |  |  |  |  |  |  |  |  |  |  | 1 | ,998^**^ | ,997^**^ |
| Day 28 (AM) |  |  |  |  |  |  |  |  |  |  |  |  | 1 | ,994^**^ |
| Day 28 (PM) |  |  |  |  |  |  |  |  |  |  |  |  |  | 1 |

**p<0.05, **p<0.001*

**S5 Table B.** Correlations for X from Day 1 (AM) to Day 31 (PM) (First-Level Agenda) – **Continued**

| **Days** | **Day 29** | | **Day 30** | | **Day 31** | |
| --- | --- | --- | --- | --- | --- | --- |
|  | **AM** | **PM** | **AM** | **PM** | **AM** | **PM** |
| Day 1 (AM) | ,955^**^ | ,940^**^ | ,940^**^ | ,935^**^ | ,922^**^ | ,897^**^ |
| Day 1 (PM) | ,963^**^ | ,952^**^ | ,949^**^ | ,947^**^ | ,932^**^ | ,904^**^ |
| Day 2 (AM) | ,954^**^ | ,941^**^ | ,942^**^ | ,938^**^ | ,925^**^ | ,897^**^ |
| Day 2 (PM) | ,970^**^ | ,960^**^ | ,958^**^ | ,957^**^ | ,935^**^ | ,903^**^ |
| Day 3 (AM) | ,971^**^ | ,961^**^ | ,960^**^ | ,957^**^ | ,930^**^ | ,892^**^ |
| Day 3 (PM) | ,972^**^ | ,963^**^ | ,958^**^ | ,957^**^ | ,934^**^ | ,900^**^ |
| Day 4 (AM) | ,961^**^ | ,946^**^ | ,945^**^ | ,941^**^ | ,929^**^ | ,902^**^ |
| Day 4 (PM) | ,948^**^ | ,935^**^ | ,932^**^ | ,929^**^ | ,914^**^ | ,890^**^ |
| Day 5 (AM) | ,958^**^ | ,944^**^ | ,949^**^ | ,944^**^ | ,916^**^ | ,877^**^ |
| Day 5 (PM) | ,963^**^ | ,949^**^ | ,948^**^ | ,945^**^ | ,931^**^ | ,905^**^ |
| Day 6 (AM) | ,973^**^ | ,961^**^ | ,961^**^ | ,958^**^ | ,936^**^ | ,901^**^ |
| Day 6 (PM) | ,964^**^ | ,953^**^ | ,947^**^ | ,946^**^ | ,931^**^ | ,910^**^ |
| Day 7 (AM) | ,961^**^ | ,945^**^ | ,946^**^ | ,943^**^ | ,931^**^ | ,904^**^ |
| Day 7 (PM) | ,974^**^ | ,965^**^ | ,957^**^ | ,957^**^ | ,934^**^ | ,906^**^ |
| Day 8 (AM) | ,984^**^ | ,978^**^ | ,980^**^ | ,976^**^ | ,938^**^ | ,890^**^ |
| Day 8 (PM) | ,989^**^ | ,987^**^ | ,984^**^ | ,984^**^ | ,945^**^ | ,898^**^ |
| Day 9 (AM) | ,990^**^ | ,992^**^ | ,988^**^ | ,989^**^ | ,950^**^ | ,907^**^ |
| Day 9 (PM) | ,986^**^ | ,992^**^ | ,984^**^ | ,985^**^ | ,936^**^ | ,892^**^ |
| Day 10 (AM) | ,992^**^ | ,990^**^ | ,986^**^ | ,987^**^ | ,953^**^ | ,912^**^ |
| Day 10 (PM) | ,978^**^ | ,987^**^ | ,977^**^ | ,980^**^ | ,924^**^ | ,875^**^ |
| Day 11 (AM) | ,973^**^ | ,982^**^ | ,978^**^ | ,977^**^ | ,918^**^ | ,856^**^ |
| Day 11 (PM) | ,970^**^ | ,967^**^ | ,966^**^ | ,964^**^ | ,936^**^ | ,900^**^ |
| Day 12 (AM) | ,990^**^ | ,987^**^ | ,987^**^ | ,984^**^ | ,952^**^ | ,909^**^ |
| Day 12 (PM) | ,982^**^ | ,985^**^ | ,979^**^ | ,978^**^ | ,930^**^ | ,883^**^ |
| Day 13 (AM) | ,978^**^ | ,984^**^ | ,980^**^ | ,977^**^ | ,922^**^ | ,869^**^ |
| Day 13 (PM) | ,980^**^ | ,976^**^ | ,973^**^ | ,971^**^ | ,936^**^ | ,897^**^ |
| Day 14 (AM) | ,985^**^ | ,984^**^ | ,986^**^ | ,983^**^ | ,943^**^ | ,897^**^ |
| Day 14 (PM) | ,982^**^ | ,985^**^ | ,981^**^ | ,979^**^ | ,933^**^ | ,887^**^ |
| Day 15 (AM) | ,979^**^ | ,975^**^ | ,978^**^ | ,975^**^ | ,935^**^ | ,881^**^ |
| Day 15 (PM) | ,987^**^ | ,987^**^ | ,984^**^ | ,982^**^ | ,937^**^ | ,887^**^ |
| Day 16 (AM) | ,988^**^ | ,985^**^ | ,985^**^ | ,982^**^ | ,941^**^ | ,891^**^ |
| Day 16 (PM) | ,985^**^ | ,982^**^ | ,977^**^ | ,976^**^ | ,940^**^ | ,900^**^ |
| Day 17 (AM) | ,991^**^ | ,988^**^ | ,988^**^ | ,985^**^ | ,952^**^ | ,911^**^ |
| Day 17 (PM) | ,965^**^ | ,956^**^ | ,956^**^ | ,953^**^ | ,933^**^ | ,903^**^ |
| Day 18 (AM) | ,987^**^ | ,979^**^ | ,983^**^ | ,981^**^ | ,957^**^ | ,913^**^ |
| Day 18 (PM) | ,985^**^ | ,987^**^ | ,981^**^ | ,981^**^ | ,934^**^ | ,887^**^ |
| Day 19 (AM) | ,990^**^ | ,988^**^ | ,987^**^ | ,985^**^ | ,946^**^ | ,902^**^ |
| Day 19 (PM) | ,987^**^ | ,986^**^ | ,983^**^ | ,982^**^ | ,944^**^ | ,904^**^ |
| Day 20 (AM) | ,990^**^ | ,987^**^ | ,985^**^ | ,982^**^ | ,947^**^ | ,906^**^ |
| Day 20 (PM) | ,991^**^ | ,991^**^ | ,987^**^ | ,986^**^ | ,952^**^ | ,912^**^ |
| Day 21 (AM) | ,993^**^ | ,992^**^ | ,991^**^ | ,989^**^ | ,954^**^ | ,909^**^ |
| Day 21 (PM) | ,991^**^ | ,992^**^ | ,987^**^ | ,986^**^ | ,948^**^ | ,903^**^ |
| Day 22 (AM) | ,994^**^ | ,990^**^ | ,990^**^ | ,987^**^ | ,947^**^ | ,898^**^ |
| Day 22 (PM) | ,976^**^ | ,985^**^ | ,977^**^ | ,976^**^ | ,916^**^ | ,864^**^ |
| Day 23 (AM) | ,981^**^ | ,987^**^ | ,984^**^ | ,980^**^ | ,921^**^ | ,868^**^ |
| Day 23 (PM) | ,990^**^ | ,987^**^ | ,985^**^ | ,983^**^ | ,940^**^ | ,895^**^ |
| Day 24 (AM) | ,994^**^ | ,986^**^ | ,987^**^ | ,985^**^ | ,953^**^ | ,911^**^ |
| Day 24 (PM) | ,970^**^ | ,971^**^ | ,965^**^ | ,968^**^ | ,916^**^ | ,871^**^ |
| Day 25 (AM) | ,990^**^ | ,988^**^ | ,986^**^ | ,985^**^ | ,946^**^ | ,902^**^ |
| Day 25 (PM) | ,991^**^ | ,993^**^ | ,990^**^ | ,989^**^ | ,946^**^ | ,899^**^ |
| Day 26 (AM) | ,995^**^ | ,991^**^ | ,993^**^ | ,991^**^ | ,954^**^ | ,907^**^ |
| Day 26 (PM) | ,996^**^ | ,993^**^ | ,993^**^ | ,991^**^ | ,968^**^ | ,931^**^ |
| Day 27 (AM) | ,996^**^ | ,991^**^ | ,991^**^ | ,989^**^ | ,960^**^ | ,917^**^ |
| Day 27 (PM) | ,996^**^ | ,997^**^ | ,996^**^ | ,994^**^ | ,957^**^ | ,910^**^ |
| Day 28 (AM) | ,998^**^ | ,996^**^ | ,997^**^ | ,994^**^ | ,962^**^ | ,915^**^ |
| Day 28 (PM) | ,994^**^ | ,996^**^ | ,991^**^ | ,991^**^ | ,949^**^ | ,901^**^ |
| Day 29 (AM) | 1 | ,995^**^ | ,995^**^ | ,993^**^ | ,967^**^ | ,924^**^ |
| Day 29 (PM) |  | 1 | ,996^**^ | ,995^**^ | ,957^**^ | ,912^**^ |
| Day 30 (AM) |  |  | 1 | ,997^**^ | ,965^**^ | ,917^**^ |
| Day 30 (PM) |  |  |  | 1 | ,969^**^ | ,922^**^ |
| Day 31 (AM) |  |  |  |  | 1 | ,979^**^ |
| Day 31 (PM) |  |  |  |  |  | 1 |

**p<0.05, **p<0.001*

**S5 Table C.** Relationships between X (AM) and Online News (PM), and between Online News (AM) and X (PM) (Intraday Relationships) - (First-Level Agenda)

| **Days** | **X (AM) – Online News (PM)** | | **Online News (AM) – X (PM)** | |
| --- | --- | --- | --- | --- |
|  | **r** | **p** | **r** | **p** |
| Day 1 | 0.883 | 0.000* | 0.898 | 0.000* |
| Day 2 | 0.886 | 0.000* | 0.927 | 0.000* |
| Day 3 | 0.916 | 0.000* | 0.932 | 0.000* |
| Day 4 | 0.902 | 0.000* | 0.894 | 0.000* |
| Day 5 | 0.902 | 0.000* | 0.898 | 0.000* |
| Day 6 | 0.910 | 0.000* | 0.913 | 0.000* |
| Day 7 | 0.901 | 0.000* | 0.940 | 0.000* |
| Day 8 | 0.936 | 0.000* | 0.954 | 0.000* |
| Day 9 | 0.922 | 0.000* | 0.910 | 0.000* |
| Day 10 | 0.931 | 0.000* | 0.933 | 0.000* |
| Day 11 | 0.899 | 0.000* | 0.950 | 0.000* |
| Day 12 | 0.933 | 0.000* | 0.944 | 0.000* |
| Day 13 | 0.917 | 0.000* | 0.947 | 0.000* |
| Day 14 | 0.943 | 0.000* | 0.942 | 0.000* |
| Day 15 | 0.944 | 0.000* | 0.955 | 0.000* |
| Day 16 | 0.948 | 0.000* | 0.965 | 0.000* |
| Day 17 | 0.937 | 0.000* | 0.950 | 0.000* |
| Day 18 | 0.938 | 0.000* | 0.934 | 0.000* |
| Day 19 | 0.930 | 0.000* | 0.936 | 0.000* |
| Day 20 | 0.948 | 0.000* | 0.947 | 0.000* |
| Day 21 | 0.955 | 0.000* | 0.959 | 0.000* |
| Day 22 | 0.943 | 0.000* | 0.943 | 0.000* |
| Day 23 | 0.919 | 0.000* | 0.945 | 0.000* |
| Day 24 | 0.944 | 0.000* | 0.942 | 0.000* |
| Day 25 | 0.931 | 0.000* | 0.945 | 0.000* |
| Day 26 | **0.957** | 0.000* | 0.938 | 0.000* |
| Day 27 | 0.951 | 0.000* | 0.950 | 0.000* |
| Day 28 | 0.952 | 0.000* | **0.968** | 0.000* |
| Day 29 | 0.963 | 0.000* | 0.966 | 0.000* |
| Day 30 | 0.938 | 0.000* | 0.947 | 0.000* |
| Day 31 | 0.926 | 0.000* | 0.933 | 0.000* |

**p<0.05*

**S5 Table D.** Relationships between X (PM) and Online News (AM), and between Online News (PM) and X (AM) (Day-to-Day Relationships) - (First-Level Agenda)

| **Days** | **X (PM) – Online News (AM)** | | **Online News (PM) – X (AM)** | |
| --- | --- | --- | --- | --- |
|  | **r** | **p** | **r** | **p** |
| Days 1 and 2 | 0.909 | 0.000* | 0.880 | 0.000* |
| Days 2 and 3 | 0.921 | 0.000* | 0.911 | 0.000* |
| Days 3 and 4 | 0.940 | 0.000* | 0.899 | 0.000* |
| Days 4 and 5 | 0.874 | 0.000* | 0.905 | 0.000* |
| Days 5 and 6 | 0.910 | 0.000* | 0.908 | 0.000* |
| Days 6 and 7 | 0.920 | 0.000* | 0.886 | 0.000* |
| Days 7 and 8 | 0.943 | 0.000* | 0.954 | 0.000* |
| Days 8 and 9 | 0.937 | 0.000* | 0.898 | 0.000* |
| Days 9 and 10 | 0.935 | 0.000* | 0.929 | 0.000* |
| Days 10 and 11 | 0.947 | 0.000* | 0.906 | 0.000* |
| Days 11 and 12 | 0.957 | 0.000* | 0.926 | 0.000* |
| Days 12 and 13 | 0.940 | 0.000* | 0.917 | 0.000* |
| Days 13 and 14 | 0.955 | 0.000* | 0.929 | 0.000* |
| Days 14 and 15 | 0.951 | 0.000* | 0.953 | 0.000* |
| Days 15 and 16 | 0.965 | 0.000* | 0.950 | 0.000* |
| Days 16 and 17 | 0.964 | 0.000* | 0.941 | 0.000* |
| Days 17 and 18 | 0.932 | 0.000* | 0.946 | 0.000* |
| Days 18 and 19 | 0.925 | 0.000* | 0.917 | 0.000* |
| Days 19 and 20 | 0.961 | 0.000* | 0.927 | 0.000* |
| Days 20 and 21 | 0.947 | 0.000* | 0.952 | 0.000* |
| Days 21 and 22 | 0.958 | 0.000* | **0.961** | 0.000* |
| Days 22 and 23 | 0.933 | 0.000* | 0.902 | 0.000* |
| Days 23 and 24 | 0.954 | 0.000* | 0.946 | 0.000* |
| Days 24 and 25 | 0.935 | 0.000* | 0.944 | 0.000* |
| Days 25 and 26 | 0.941 | 0.000* | 0.938 | 0.000* |
| Days 26 and 27 | 0.947 | 0.000* | 0.953 | 0.000* |
| Days 27 and 28 | 0.965 | 0.000* | 0.951 | 0.000* |
| Days 28 and 29 | **0.971** | 0.000* | 0.952 | 0.000* |
| Days 29 and 30 | 0.947 | 0.000* | 0.955 | 0.000* |
| Days 30 and 31 | 0.927 | 0.000* | 0.928 | 0.000* |

**p<0.05*

**Appendix S6. Correlation Scores (Second-Level Agenda)**

**S6 Table A.** Correlations for Online News from Day 1 (AM) to Day 31 (PM) (Second-Level Agenda)

| **Days** | **Day 1** | | **Day 2** | | **Day 3** | | **Day 4** | | **Day 5** | | **Day 6** | | **Day 7** | |
| --- | --- | --- | --- | --- | --- | --- | --- | --- | --- | --- | --- | --- | --- | --- |
|  | **AM** | **PM** | **AM** | **PM** | **AM** | **PM** | **AM** | **PM** | **AM** | **PM** | **AM** | **PM** | **AM** | **PM** |
| Day 1 (AM) | 1 | ,965^**^ | ,943^**^ | ,904^**^ | ,855^**^ | ,883^**^ | ,895^**^ | ,913^**^ | ,874^**^ | ,878^**^ | ,869^**^ | ,941^**^ | ,892^**^ | ,897^**^ |
| Day 1 (PM) |  | 1 | ,925^**^ | ,928^**^ | ,869^**^ | ,903^**^ | ,883^**^ | ,933^**^ | ,884^**^ | ,874^**^ | ,854^**^ | ,939^**^ | ,881^**^ | ,892^**^ |
| Day 2 (AM) |  |  | 1 | ,914^**^ | ,855^**^ | ,889^**^ | ,869^**^ | ,910^**^ | ,847^**^ | ,860^**^ | ,873^**^ | ,922^**^ | ,861^**^ | ,902^**^ |
| Day 2 (PM) |  |  |  | 1 | ,919^**^ | ,935^**^ | ,830^**^ | ,932^**^ | ,853^**^ | ,862^**^ | ,859^**^ | ,949^**^ | ,856^**^ | ,918^**^ |
| Day 3 (AM) |  |  |  |  | 1 | ,891^**^ | ,780^**^ | ,870^**^ | ,798^**^ | ,841^**^ | ,835^**^ | ,901^**^ | ,809^**^ | ,890^**^ |
| Day 3 (PM) |  |  |  |  |  | 1 | ,889^**^ | ,934^**^ | ,879^**^ | ,881^**^ | ,889^**^ | ,924^**^ | ,867^**^ | ,933^**^ |
| Day 4 (AM) |  |  |  |  |  |  | 1 | ,852^**^ | ,936^**^ | ,913^**^ | ,912^**^ | ,896^**^ | ,921^**^ | ,931^**^ |
| Day 4 (PM) |  |  |  |  |  |  |  | 1 | ,869^**^ | ,833^**^ | ,863^**^ | ,929^**^ | ,864^**^ | ,893^**^ |
| Day 5 (AM) |  |  |  |  |  |  |  |  | 1 | ,927^**^ | ,885^**^ | ,900^**^ | ,937^**^ | ,917^**^ |
| Day 5 (PM) |  |  |  |  |  |  |  |  |  | 1 | ,914^**^ | ,927^**^ | ,941^**^ | ,920^**^ |
| Day 6 (AM) |  |  |  |  |  |  |  |  |  |  | 1 | ,901^**^ | ,891^**^ | ,940^**^ |
| Day 6 (PM) |  |  |  |  |  |  |  |  |  |  |  | 1 | ,932^**^ | ,933^**^ |
| Day 7 (AM) |  |  |  |  |  |  |  |  |  |  |  |  | 1 | ,908^**^ |
| Day 7 (PM) |  |  |  |  |  |  |  |  |  |  |  |  |  | 1 |

**p<0.05, **p<0.001*

**S6 Table A.** Correlations for Online News from Day 1 (AM) to Day 31 (PM) (Second-Level Agenda) - **Continued**

| **Days** | **Day 8** | | **Day 9** | | **Day 10** | | **Day 11** | | **Day 12** | | **Day 13** | | **Day 14** | |
| --- | --- | --- | --- | --- | --- | --- | --- | --- | --- | --- | --- | --- | --- | --- |
|  | **AM** | **PM** | **AM** | **PM** | **AM** | **PM** | **AM** | **PM** | **AM** | **PM** | **AM** | **PM** | **AM** | **PM** |
| Day 1 (AM) | ,881^**^ | ,555^**^ | ,753^**^ | ,873^**^ | ,852^**^ | ,894^**^ | ,897^**^ | ,904^**^ | ,901^**^ | ,932^**^ | ,853^**^ | ,917^**^ | ,891^**^ | ,907^**^ |
| Day 1 (PM) | ,868^**^ | ,584^**^ | ,741^**^ | ,890^**^ | ,824^**^ | ,889^**^ | ,876^**^ | ,897^**^ | ,875^**^ | ,908^**^ | ,859^**^ | ,886^**^ | ,871^**^ | ,900^**^ |
| Day 2 (AM) | ,890^**^ | ,558^**^ | ,743^**^ | ,871^**^ | ,826^**^ | ,866^**^ | ,870^**^ | ,918^**^ | ,886^**^ | ,890^**^ | ,822^**^ | ,894^**^ | ,845^**^ | ,860^**^ |
| Day 2 (PM) | ,867^**^ | ,610^**^ | ,746^**^ | ,925^**^ | ,781^**^ | ,926^**^ | ,845^**^ | ,930^**^ | ,862^**^ | ,885^**^ | ,815^**^ | ,879^**^ | ,837^**^ | ,843^**^ |
| Day 3 (AM) | ,802^**^ | ,583^**^ | ,718^**^ | ,892^**^ | ,742^**^ | ,899^**^ | ,812^**^ | ,907^**^ | ,823^**^ | ,790^**^ | ,752^**^ | ,871^**^ | ,733^**^ | ,751^**^ |
| Day 3 (PM) | ,899^**^ | ,691^**^ | ,819^**^ | ,921^**^ | ,788^**^ | ,904^**^ | ,904^**^ | ,914^**^ | ,854^**^ | ,905^**^ | ,854^**^ | ,885^**^ | ,836^**^ | ,822^**^ |
| Day 4 (AM) | ,919^**^ | ,788^**^ | ,911^**^ | ,845^**^ | ,858^**^ | ,834^**^ | ,923^**^ | ,827^**^ | ,856^**^ | ,899^**^ | ,940^**^ | ,881^**^ | ,866^**^ | ,855^**^ |
| Day 4 (PM) | ,833^**^ | ,569^**^ | ,725^**^ | ,907^**^ | ,802^**^ | ,867^**^ | ,864^**^ | ,883^**^ | ,830^**^ | ,895^**^ | ,811^**^ | ,880^**^ | ,836^**^ | ,874^**^ |
| Day 5 (AM) | ,891^**^ | ,806^**^ | ,901^**^ | ,875^**^ | ,855^**^ | ,828^**^ | ,877^**^ | ,837^**^ | ,826^**^ | ,869^**^ | ,925^**^ | ,862^**^ | ,844^**^ | ,872^**^ |
| Day 5 (PM) | ,902^**^ | ,776^**^ | ,886^**^ | ,869^**^ | ,901^**^ | ,849^**^ | ,890^**^ | ,884^**^ | ,893^**^ | ,871^**^ | ,911^**^ | ,918^**^ | ,808^**^ | ,829^**^ |
| Day 6 (AM) | ,910^**^ | ,697^**^ | ,839^**^ | ,871^**^ | ,867^**^ | ,855^**^ | ,928^**^ | ,871^**^ | ,879^**^ | ,889^**^ | ,864^**^ | ,918^**^ | ,794^**^ | ,837^**^ |
| Day 6 (PM) | ,882^**^ | ,674^**^ | ,818^**^ | ,925^**^ | ,867^**^ | ,916^**^ | ,888^**^ | ,919^**^ | ,901^**^ | ,905^**^ | ,882^**^ | ,934^**^ | ,853^**^ | ,871^**^ |
| Day 7 (AM) | ,900^**^ | ,770^**^ | ,868^**^ | ,856^**^ | ,906^**^ | ,820^**^ | ,874^**^ | ,849^**^ | ,868^**^ | ,872^**^ | ,917^**^ | ,894^**^ | ,852^**^ | ,871^**^ |
| Day 7 (PM) | ,940^**^ | ,761^**^ | ,879^**^ | ,921^**^ | ,833^**^ | ,896^**^ | ,914^**^ | ,907^**^ | ,893^**^ | ,904^**^ | ,889^**^ | ,912^**^ | ,843^**^ | ,842^**^ |
| Day 8 (AM) | 1 | ,735^**^ | ,860^**^ | ,862^**^ | ,877^**^ | ,844^**^ | ,914^**^ | ,895^**^ | ,917^**^ | ,912^**^ | ,886^**^ | ,886^**^ | ,860^**^ | ,845^**^ |
| Day 8 (PM) |  | 1 | ,923^**^ | ,692^**^ | ,612^**^ | ,624^**^ | ,640^**^ | ,589^**^ | ,578^**^ | ,602^**^ | ,803^**^ | ,629^**^ | ,591^**^ | ,538^**^ |
| Day 9 (AM) |  |  | 1 | ,817^**^ | ,763^**^ | ,781^**^ | ,828^**^ | ,764^**^ | ,760^**^ | ,781^**^ | ,881^**^ | ,807^**^ | ,756^**^ | ,698^**^ |
| Day 9 (PM) |  |  |  | 1 | ,803^**^ | ,946^**^ | ,857^**^ | ,904^**^ | ,859^**^ | ,883^**^ | ,842^**^ | ,911^**^ | ,834^**^ | ,844^**^ |
| Day 10 (AM) |  |  |  |  | 1 | ,763^**^ | ,861^**^ | ,808^**^ | ,908^**^ | ,857^**^ | ,844^**^ | ,905^**^ | ,801^**^ | ,885^**^ |
| Day 10 (PM) |  |  |  |  |  | 1 | ,856^**^ | ,894^**^ | ,842^**^ | ,872^**^ | ,811^**^ | ,898^**^ | ,845^**^ | ,840^**^ |
| Day 11 (AM) |  |  |  |  |  |  | 1 | ,881^**^ | ,901^**^ | ,934^**^ | ,867^**^ | ,902^**^ | ,856^**^ | ,826^**^ |
| Day 11 (PM) |  |  |  |  |  |  |  | 1 | ,923^**^ | ,898^**^ | ,825^**^ | ,910^**^ | ,853^**^ | ,801^**^ |
| Day 12 (AM) |  |  |  |  |  |  |  |  | 1 | ,918^**^ | ,852^**^ | ,915^**^ | ,840^**^ | ,837^**^ |
| Day 12 (PM) |  |  |  |  |  |  |  |  |  | 1 | ,878^**^ | ,898^**^ | ,918^**^ | ,889^**^ |
| Day 13 (AM) |  |  |  |  |  |  |  |  |  |  | 1 | ,850^**^ | ,858^**^ | ,830^**^ |
| Day 13 (PM) |  |  |  |  |  |  |  |  |  |  |  | 1 | ,842^**^ | ,877^**^ |
| Day 14 (AM) |  |  |  |  |  |  |  |  |  |  |  |  | 1 | ,895^**^ |
| Day 14 (PM) |  |  |  |  |  |  |  |  |  |  |  |  |  | 1 |

**p<0.05, **p<0.001*

**S6 Table A.** Correlations for Online News from Day 1 (AM) to Day 31 (PM) (Second-Level Agenda) - **Continued**

| **Days** | **Day 15** | | **Day 16** | | **Day 17** | | **Day 18** | | **Day 19** | | **Day 20** | | **Day 21** | |
| --- | --- | --- | --- | --- | --- | --- | --- | --- | --- | --- | --- | --- | --- | --- |
|  | **AM** | **PM** | **AM** | **PM** | **AM** | **PM** | **AM** | **PM** | **AM** | **PM** | **AM** | **PM** | **AM** | **PM** |
| Day 1 (AM) | ,895^**^ | ,931^**^ | ,920^**^ | ,936^**^ | ,932^**^ | ,931^**^ | ,946^**^ | ,871^**^ | ,796^**^ | ,889^**^ | ,854^**^ | ,923^**^ | ,932^**^ | ,869^**^ |
| Day 1 (PM) | ,887^**^ | ,916^**^ | ,906^**^ | ,932^**^ | ,896^**^ | ,926^**^ | ,928^**^ | ,846^**^ | ,804^**^ | ,897^**^ | ,832^**^ | ,902^**^ | ,917^**^ | ,873^**^ |
| Day 2 (AM) | ,832^**^ | ,915^**^ | ,869^**^ | ,916^**^ | ,890^**^ | ,912^**^ | ,936^**^ | ,869^**^ | ,742^**^ | ,872^**^ | ,812^**^ | ,896^**^ | ,923^**^ | ,878^**^ |
| Day 2 (PM) | ,834^**^ | ,904^**^ | ,861^**^ | ,929^**^ | ,858^**^ | ,928^**^ | ,898^**^ | ,888^**^ | ,791^**^ | ,912^**^ | ,785^**^ | ,892^**^ | ,929^**^ | ,906^**^ |
| Day 3 (AM) | ,778^**^ | ,852^**^ | ,804^**^ | ,868^**^ | ,788^**^ | ,869^**^ | ,867^**^ | ,830^**^ | ,735^**^ | ,850^**^ | ,736^**^ | ,859^**^ | ,890^**^ | ,853^**^ |
| Day 3 (PM) | ,851^**^ | ,914^**^ | ,878^**^ | ,926^**^ | ,882^**^ | ,911^**^ | ,878^**^ | ,906^**^ | ,825^**^ | ,883^**^ | ,789^**^ | ,884^**^ | ,920^**^ | ,897^**^ |
| Day 4 (AM) | ,869^**^ | ,902^**^ | ,869^**^ | ,876^**^ | ,899^**^ | ,871^**^ | ,879^**^ | ,836^**^ | ,764^**^ | ,819^**^ | ,813^**^ | ,871^**^ | ,882^**^ | ,829^**^ |
| Day 4 (PM) | ,872^**^ | ,890^**^ | ,878^**^ | ,904^**^ | ,876^**^ | ,922^**^ | ,893^**^ | ,884^**^ | ,786^**^ | ,924^**^ | ,807^**^ | ,902^**^ | ,913^**^ | ,878^**^ |
| Day 5 (AM) | ,858^**^ | ,877^**^ | ,859^**^ | ,860^**^ | ,846^**^ | ,874^**^ | ,851^**^ | ,798^**^ | ,748^**^ | ,878^**^ | ,806^**^ | ,855^**^ | ,869^**^ | ,854^**^ |
| Day 5 (PM) | ,848^**^ | ,891^**^ | ,853^**^ | ,873^**^ | ,830^**^ | ,870^**^ | ,879^**^ | ,835^**^ | ,778^**^ | ,843^**^ | ,829^**^ | ,899^**^ | ,887^**^ | ,885^**^ |
| Day 6 (AM) | ,851^**^ | ,900^**^ | ,870^**^ | ,865^**^ | ,852^**^ | ,867^**^ | ,848^**^ | ,879^**^ | ,783^**^ | ,868^**^ | ,812^**^ | ,913^**^ | ,926^**^ | ,914^**^ |
| Day 6 (PM) | ,873^**^ | ,917^**^ | ,888^**^ | ,926^**^ | ,883^**^ | ,942^**^ | ,943^**^ | ,897^**^ | ,784^**^ | ,913^**^ | ,835^**^ | ,931^**^ | ,939^**^ | ,906^**^ |
| Day 7 (AM) | ,859^**^ | ,886^**^ | ,860^**^ | ,865^**^ | ,849^**^ | ,861^**^ | ,874^**^ | ,826^**^ | ,736^**^ | ,870^**^ | ,831^**^ | ,896^**^ | ,880^**^ | ,844^**^ |
| Day 7 (PM) | ,848^**^ | ,943^**^ | ,895^**^ | ,911^**^ | ,883^**^ | ,907^**^ | ,886^**^ | ,911^**^ | ,800^**^ | ,888^**^ | ,788^**^ | ,909^**^ | ,928^**^ | ,919^**^ |
| Day 8 (AM) | ,838^**^ | ,934^**^ | ,899^**^ | ,894^**^ | ,887^**^ | ,856^**^ | ,853^**^ | ,875^**^ | ,789^**^ | ,855^**^ | ,804^**^ | ,890^**^ | ,896^**^ | ,901^**^ |
| Day 8 (PM) | ,534^**^ | ,651^**^ | ,556^**^ | ,625^**^ | ,558^**^ | ,597^**^ | ,572^**^ | ,579^**^ | ,516^**^ | ,571^**^ | ,489^**^ | ,562^**^ | ,585^**^ | ,607^**^ |
| Day 9 (AM) | ,729^**^ | ,807^**^ | ,750^**^ | ,781^**^ | ,777^**^ | ,777^**^ | ,772^**^ | ,742^**^ | ,656^**^ | ,726^**^ | ,691^**^ | ,739^**^ | ,757^**^ | ,759^**^ |
| Day 9 (PM) | ,836^**^ | ,915^**^ | ,870^**^ | ,923^**^ | ,859^**^ | ,940^**^ | ,891^**^ | ,902^**^ | ,785^**^ | ,934^**^ | ,798^**^ | ,878^**^ | ,903^**^ | ,906^**^ |
| Day 10 (AM) | ,860^**^ | ,858^**^ | ,874^**^ | ,796^**^ | ,823^**^ | ,828^**^ | ,849^**^ | ,806^**^ | ,741^**^ | ,858^**^ | ,875^**^ | ,915^**^ | ,849^**^ | ,852^**^ |
| Day 10 (PM) | ,829^**^ | ,897^**^ | ,848^**^ | ,936^**^ | ,871^**^ | ,941^**^ | ,891^**^ | ,872^**^ | ,760^**^ | ,887^**^ | ,785^**^ | ,868^**^ | ,914^**^ | ,872^**^ |
| Day 11 (AM) | ,898^**^ | ,907^**^ | ,902^**^ | ,879^**^ | ,928^**^ | ,866^**^ | ,876^**^ | ,872^**^ | ,815^**^ | ,845^**^ | ,813^**^ | ,899^**^ | ,915^**^ | ,885^**^ |
| Day 11 (PM) | ,866^**^ | ,933^**^ | ,888^**^ | ,920^**^ | ,881^**^ | ,904^**^ | ,920^**^ | ,908^**^ | ,832^**^ | ,875^**^ | ,840^**^ | ,897^**^ | ,918^**^ | ,911^**^ |
| Day 12 (AM) | ,883^**^ | ,928^**^ | ,907^**^ | ,883^**^ | ,882^**^ | ,869^**^ | ,912^**^ | ,900^**^ | ,838^**^ | ,851^**^ | ,878^**^ | ,927^**^ | ,902^**^ | ,916^**^ |
| Day 12 (PM) | ,921^**^ | ,937^**^ | ,927^**^ | ,932^**^ | ,945^**^ | ,914^**^ | ,890^**^ | ,929^**^ | ,856^**^ | ,882^**^ | ,859^**^ | ,929^**^ | ,925^**^ | ,910^**^ |
| Day 13 (AM) | ,852^**^ | ,883^**^ | ,844^**^ | ,853^**^ | ,841^**^ | ,845^**^ | ,855^**^ | ,830^**^ | ,812^**^ | ,805^**^ | ,809^**^ | ,842^**^ | ,847^**^ | ,825^**^ |
| Day 13 (PM) | ,889^**^ | ,911^**^ | ,915^**^ | ,914^**^ | ,898^**^ | ,931^**^ | ,924^**^ | ,892^**^ | ,769^**^ | ,910^**^ | ,878^**^ | ,936^**^ | ,923^**^ | ,892^**^ |
| Day 14 (AM) | ,918^**^ | ,908^**^ | ,891^**^ | ,902^**^ | ,928^**^ | ,868^**^ | ,858^**^ | ,869^**^ | ,799^**^ | ,859^**^ | ,851^**^ | ,871^**^ | ,866^**^ | ,804^**^ |
| Day 14 (PM) | ,900^**^ | ,885^**^ | ,910^**^ | ,878^**^ | ,872^**^ | ,905^**^ | ,846^**^ | ,820^**^ | ,758^**^ | ,932^**^ | ,894^**^ | ,914^**^ | ,892^**^ | ,847^**^ |
| Day 15 (AM) | 1 | ,905^**^ | ,927^**^ | ,874^**^ | ,929^**^ | ,882^**^ | ,877^**^ | ,879^**^ | ,855^**^ | ,881^**^ | ,928^**^ | ,915^**^ | ,890^**^ | ,849^**^ |
| Day 15 (PM) |  | 1 | ,931^**^ | ,921^**^ | ,917^**^ | ,902^**^ | ,907^**^ | ,943^**^ | ,891^**^ | ,896^**^ | ,869^**^ | ,934^**^ | ,939^**^ | ,923^**^ |
| Day 16 (AM) |  |  | 1 | ,888^**^ | ,931^**^ | ,915^**^ | ,888^**^ | ,890^**^ | ,842^**^ | ,922^**^ | ,891^**^ | ,926^**^ | ,904^**^ | ,903^**^ |
| Day 16 (PM) |  |  |  | 1 | ,925^**^ | ,943^**^ | ,910^**^ | ,899^**^ | ,783^**^ | ,895^**^ | ,828^**^ | ,896^**^ | ,925^**^ | ,877^**^ |
| Day 17 (AM) |  |  |  |  | 1 | ,914^**^ | ,914^**^ | ,890^**^ | ,793^**^ | ,866^**^ | ,860^**^ | ,899^**^ | ,905^**^ | ,846^**^ |
| Day 17 (PM) |  |  |  |  |  | 1 | ,943^**^ | ,889^**^ | ,767^**^ | ,937^**^ | ,856^**^ | ,910^**^ | ,922^**^ | ,888^**^ |
| Day 18 (AM) |  |  |  |  |  |  | 1 | ,872^**^ | ,771^**^ | ,869^**^ | ,858^**^ | ,904^**^ | ,905^**^ | ,851^**^ |
| Day 18 (PM) |  |  |  |  |  |  |  | 1 | ,879^**^ | ,874^**^ | ,834^**^ | ,925^**^ | ,923^**^ | ,913^**^ |
| Day 19 (AM) |  |  |  |  |  |  |  |  | 1 | ,778^**^ | ,819^**^ | ,836^**^ | ,824^**^ | ,854^**^ |
| Day 19 (PM) |  |  |  |  |  |  |  |  |  | 1 | ,856^**^ | ,920^**^ | ,911^**^ | ,908^**^ |
| Day 20 (AM) |  |  |  |  |  |  |  |  |  |  | 1 | ,894^**^ | ,856^**^ | ,841^**^ |
| Day 20 (PM) |  |  |  |  |  |  |  |  |  |  |  | 1 | ,963^**^ | ,927^**^ |
| Day 21 (AM) |  |  |  |  |  |  |  |  |  |  |  |  | 1 | ,941^**^ |
| Day 21 (PM) |  |  |  |  |  |  |  |  |  |  |  |  |  | 1 |

**p<0.05, **p<0.001*

**S6 Table A.** Correlations for Online News from Day 1 (AM) to Day 31 (PM) (Second-Level Agenda) - **Continued**

| **Days** | **Day 22** | | **Day 23** | | **Day 24** | | **Day 25** | | **Day 26** | | **Day 27** | | **Day 28** | |
| --- | --- | --- | --- | --- | --- | --- | --- | --- | --- | --- | --- | --- | --- | --- |
|  | **AM** | **PM** | **AM** | **PM** | **AM** | **PM** | **AM** | **PM** | **AM** | **PM** | **AM** | **PM** | **AM** | **PM** |
| Day 1 (AM) | ,921^**^ | ,946^**^ | ,921^**^ | ,906^**^ | ,858^**^ | ,910^**^ | ,910^**^ | ,914^**^ | ,907^**^ | ,902^**^ | ,908^**^ | ,938^**^ | ,936^**^ | ,912^**^ |
| Day 1 (PM) | ,899^**^ | ,929^**^ | ,921^**^ | ,918^**^ | ,861^**^ | ,901^**^ | ,890^**^ | ,896^**^ | ,858^**^ | ,852^**^ | ,879^**^ | ,923^**^ | ,908^**^ | ,915^**^ |
| Day 2 (AM) | ,862^**^ | ,878^**^ | ,851^**^ | ,860^**^ | ,782^**^ | ,874^**^ | ,867^**^ | ,915^**^ | ,890^**^ | ,885^**^ | ,848^**^ | ,891^**^ | ,875^**^ | ,911^**^ |
| Day 2 (PM) | ,879^**^ | ,853^**^ | ,865^**^ | ,912^**^ | ,792^**^ | ,908^**^ | ,859^**^ | ,915^**^ | ,848^**^ | ,833^**^ | ,854^**^ | ,876^**^ | ,880^**^ | ,940^**^ |
| Day 3 (AM) | ,824^**^ | ,774^**^ | ,813^**^ | ,837^**^ | ,706^**^ | ,883^**^ | ,819^**^ | ,842^**^ | ,803^**^ | ,791^**^ | ,788^**^ | ,813^**^ | ,816^**^ | ,888^**^ |
| Day 3 (PM) | ,850^**^ | ,831^**^ | ,849^**^ | ,912^**^ | ,779^**^ | ,911^**^ | ,891^**^ | ,912^**^ | ,848^**^ | ,836^**^ | ,857^**^ | ,872^**^ | ,884^**^ | ,913^**^ |
| Day 4 (AM) | ,846^**^ | ,889^**^ | ,857^**^ | ,871^**^ | ,813^**^ | ,876^**^ | ,887^**^ | ,884^**^ | ,875^**^ | ,872^**^ | ,878^**^ | ,882^**^ | ,889^**^ | ,842^**^ |
| Day 4 (PM) | ,872^**^ | ,845^**^ | ,856^**^ | ,913^**^ | ,861^**^ | ,904^**^ | ,877^**^ | ,892^**^ | ,835^**^ | ,830^**^ | ,860^**^ | ,889^**^ | ,878^**^ | ,926^**^ |
| Day 5 (AM) | ,848^**^ | ,866^**^ | ,846^**^ | ,893^**^ | ,849^**^ | ,871^**^ | ,864^**^ | ,855^**^ | ,857^**^ | ,832^**^ | ,857^**^ | ,886^**^ | ,871^**^ | ,869^**^ |
| Day 5 (PM) | ,868^**^ | ,875^**^ | ,867^**^ | ,868^**^ | ,798^**^ | ,874^**^ | ,860^**^ | ,888^**^ | ,893^**^ | ,879^**^ | ,864^**^ | ,888^**^ | ,875^**^ | ,876^**^ |
| Day 6 (AM) | ,863^**^ | ,860^**^ | ,839^**^ | ,856^**^ | ,782^**^ | ,875^**^ | ,900^**^ | ,885^**^ | ,913^**^ | ,899^**^ | ,911^**^ | ,880^**^ | ,872^**^ | ,883^**^ |
| Day 6 (PM) | ,912^**^ | ,903^**^ | ,892^**^ | ,908^**^ | ,843^**^ | ,930^**^ | ,894^**^ | ,927^**^ | ,892^**^ | ,895^**^ | ,897^**^ | ,913^**^ | ,912^**^ | ,950^**^ |
| Day 7 (AM) | ,893^**^ | ,883^**^ | ,862^**^ | ,871^**^ | ,857^**^ | ,869^**^ | ,854^**^ | ,890^**^ | ,890^**^ | ,871^**^ | ,870^**^ | ,903^**^ | ,884^**^ | ,879^**^ |
| Day 7 (PM) | ,869^**^ | ,862^**^ | ,856^**^ | ,905^**^ | ,800^**^ | ,919^**^ | ,881^**^ | ,904^**^ | ,883^**^ | ,885^**^ | ,895^**^ | ,891^**^ | ,891^**^ | ,917^**^ |
| Day 8 (AM) | ,857^**^ | ,876^**^ | ,868^**^ | ,872^**^ | ,788^**^ | ,871^**^ | ,884^**^ | ,905^**^ | ,906^**^ | ,877^**^ | ,880^**^ | ,908^**^ | ,883^**^ | ,873^**^ |
| Day 8 (PM) | ,556^**^ | ,551^**^ | ,557^**^ | ,615^**^ | ,507^**^ | ,605^**^ | ,559^**^ | ,597^**^ | ,567^**^ | ,554^**^ | ,561^**^ | ,577^**^ | ,566^**^ | ,585^**^ |
| Day 9 (AM) | ,712^**^ | ,732^**^ | ,713^**^ | ,766^**^ | ,665^**^ | ,781^**^ | ,765^**^ | ,777^**^ | ,768^**^ | ,754^**^ | ,755^**^ | ,756^**^ | ,760^**^ | ,754^**^ |
| Day 9 (PM) | ,858^**^ | ,831^**^ | ,848^**^ | ,901^**^ | ,800^**^ | ,897^**^ | ,850^**^ | ,867^**^ | ,843^**^ | ,830^**^ | ,847^**^ | ,868^**^ | ,859^**^ | ,935^**^ |
| Day 10 (AM) | ,868^**^ | ,891^**^ | ,877^**^ | ,823^**^ | ,857^**^ | ,839^**^ | ,870^**^ | ,868^**^ | ,916^**^ | ,900^**^ | ,874^**^ | ,915^**^ | ,872^**^ | ,841^**^ |
| Day 10 (PM) | ,879^**^ | ,847^**^ | ,871^**^ | ,894^**^ | ,758^**^ | ,899^**^ | ,862^**^ | ,872^**^ | ,857^**^ | ,828^**^ | ,844^**^ | ,856^**^ | ,875^**^ | ,910^**^ |
| Day 11 (AM) | ,857^**^ | ,896^**^ | ,859^**^ | ,877^**^ | ,815^**^ | ,900^**^ | ,935^**^ | ,897^**^ | ,914^**^ | ,896^**^ | ,931^**^ | ,894^**^ | ,917^**^ | ,865^**^ |
| Day 11 (PM) | ,881^**^ | ,864^**^ | ,857^**^ | ,876^**^ | ,762^**^ | ,884^**^ | ,878^**^ | ,932^**^ | ,893^**^ | ,869^**^ | ,864^**^ | ,883^**^ | ,877^**^ | ,931^**^ |
| Day 12 (AM) | ,903^**^ | ,915^**^ | ,896^**^ | ,854^**^ | ,819^**^ | ,883^**^ | ,900^**^ | ,923^**^ | ,924^**^ | ,916^**^ | ,899^**^ | ,920^**^ | ,902^**^ | ,897^**^ |
| Day 12 (PM) | ,917^**^ | ,934^**^ | ,898^**^ | ,930^**^ | ,878^**^ | ,926^**^ | ,928^**^ | ,935^**^ | ,921^**^ | ,904^**^ | ,931^**^ | ,931^**^ | ,951^**^ | ,902^**^ |
| Day 13 (AM) | ,860^**^ | ,887^**^ | ,851^**^ | ,857^**^ | ,805^**^ | ,837^**^ | ,851^**^ | ,862^**^ | ,848^**^ | ,830^**^ | ,832^**^ | ,859^**^ | ,855^**^ | ,818^**^ |
| Day 13 (PM) | ,878^**^ | ,891^**^ | ,895^**^ | ,876^**^ | ,819^**^ | ,892^**^ | ,897^**^ | ,908^**^ | ,918^**^ | ,923^**^ | ,915^**^ | ,917^**^ | ,891^**^ | ,915^**^ |
| Day 14 (AM) | ,888^**^ | ,902^**^ | ,878^**^ | ,886^**^ | ,843^**^ | ,855^**^ | ,866^**^ | ,913^**^ | ,882^**^ | ,828^**^ | ,872^**^ | ,874^**^ | ,883^**^ | ,838^**^ |
| Day 14 (PM) | ,907^**^ | ,927^**^ | ,938^**^ | ,916^**^ | ,921^**^ | ,871^**^ | ,886^**^ | ,867^**^ | ,899^**^ | ,868^**^ | ,899^**^ | ,942^**^ | ,902^**^ | ,880^**^ |
| Day 15 (AM) | ,904^**^ | ,928^**^ | ,917^**^ | ,906^**^ | ,893^**^ | ,884^**^ | ,925^**^ | ,910^**^ | ,898^**^ | ,876^**^ | ,917^**^ | ,917^**^ | ,916^**^ | ,870^**^ |
| Day 15 (PM) | ,928^**^ | ,921^**^ | ,912^**^ | ,911^**^ | ,838^**^ | ,904^**^ | ,914^**^ | ,936^**^ | ,922^**^ | ,913^**^ | ,914^**^ | ,933^**^ | ,915^**^ | ,925^**^ |
| Day 16 (AM) | ,885^**^ | ,930^**^ | ,928^**^ | ,934^**^ | ,897^**^ | ,910^**^ | ,926^**^ | ,906^**^ | ,914^**^ | ,916^**^ | ,943^**^ | ,957^**^ | ,934^**^ | ,912^**^ |
| Day 16 (PM) | ,886^**^ | ,881^**^ | ,887^**^ | ,910^**^ | ,794^**^ | ,899^**^ | ,878^**^ | ,917^**^ | ,870^**^ | ,842^**^ | ,867^**^ | ,889^**^ | ,899^**^ | ,911^**^ |
| Day 17 (AM) | ,869^**^ | ,910^**^ | ,879^**^ | ,910^**^ | ,846^**^ | ,904^**^ | ,920^**^ | ,921^**^ | ,897^**^ | ,886^**^ | ,913^**^ | ,910^**^ | ,923^**^ | ,870^**^ |
| Day 17 (PM) | ,883^**^ | ,894^**^ | ,895^**^ | ,940^**^ | ,849^**^ | ,924^**^ | ,886^**^ | ,905^**^ | ,878^**^ | ,873^**^ | ,885^**^ | ,913^**^ | ,913^**^ | ,947^**^ |
| Day 18 (AM) | ,884^**^ | ,903^**^ | ,872^**^ | ,869^**^ | ,805^**^ | ,891^**^ | ,876^**^ | ,923^**^ | ,886^**^ | ,888^**^ | ,860^**^ | ,891^**^ | ,886^**^ | ,918^**^ |
| Day 18 (PM) | ,899^**^ | ,856^**^ | ,844^**^ | ,881^**^ | ,805^**^ | ,893^**^ | ,883^**^ | ,925^**^ | ,884^**^ | ,889^**^ | ,885^**^ | ,876^**^ | ,890^**^ | ,909^**^ |
| Day 19 (AM) | ,843^**^ | ,851^**^ | ,845^**^ | ,823^**^ | ,761^**^ | ,801^**^ | ,858^**^ | ,838^**^ | ,805^**^ | ,795^**^ | ,837^**^ | ,824^**^ | ,824^**^ | ,800^**^ |
| Day 19 (PM) | ,882^**^ | ,875^**^ | ,894^**^ | ,927^**^ | ,889^**^ | ,900^**^ | ,885^**^ | ,882^**^ | ,886^**^ | ,857^**^ | ,898^**^ | ,921^**^ | ,886^**^ | ,940^**^ |
| Day 20 (AM) | ,883^**^ | ,900^**^ | ,915^**^ | ,850^**^ | ,857^**^ | ,832^**^ | ,914^**^ | ,882^**^ | ,898^**^ | ,879^**^ | ,874^**^ | ,910^**^ | ,874^**^ | ,851^**^ |
| Day 20 (PM) | ,935^**^ | ,927^**^ | ,921^**^ | ,917^**^ | ,892^**^ | ,933^**^ | ,928^**^ | ,937^**^ | ,955^**^ | ,944^**^ | ,945^**^ | ,946^**^ | ,930^**^ | ,929^**^ |
| Day 21 (AM) | ,933^**^ | ,914^**^ | ,902^**^ | ,921^**^ | ,855^**^ | ,931^**^ | ,940^**^ | ,928^**^ | ,940^**^ | ,927^**^ | ,934^**^ | ,929^**^ | ,937^**^ | ,944^**^ |
| Day 21 (PM) | ,895^**^ | ,880^**^ | ,875^**^ | ,907^**^ | ,842^**^ | ,917^**^ | ,916^**^ | ,898^**^ | ,908^**^ | ,903^**^ | ,910^**^ | ,919^**^ | ,907^**^ | ,940^**^ |
| Day 22 (AM) | 1 | ,940^**^ | ,926^**^ | ,908^**^ | ,896^**^ | ,893^**^ | ,903^**^ | ,919^**^ | ,926^**^ | ,901^**^ | ,896^**^ | ,935^**^ | ,936^**^ | ,910^**^ |
| Day 22 (PM) |  | 1 | ,953^**^ | ,912^**^ | ,911^**^ | ,892^**^ | ,927^**^ | ,912^**^ | ,930^**^ | ,927^**^ | ,940^**^ | ,958^**^ | ,948^**^ | ,884^**^ |
| Day 23 (AM) |  |  | 1 | ,921^**^ | ,892^**^ | ,885^**^ | ,922^**^ | ,885^**^ | ,900^**^ | ,881^**^ | ,910^**^ | ,954^**^ | ,916^**^ | ,874^**^ |
| Day 23 (PM) |  |  |  | 1 | ,924^**^ | ,949^**^ | ,908^**^ | ,900^**^ | ,888^**^ | ,879^**^ | ,911^**^ | ,947^**^ | ,947^**^ | ,927^**^ |
| Day 24 (AM) |  |  |  |  | 1 | ,880^**^ | ,872^**^ | ,828^**^ | ,863^**^ | ,864^**^ | ,893^**^ | ,937^**^ | ,917^**^ | ,864^**^ |
| Day 24 (PM) |  |  |  |  |  | 1 | ,922^**^ | ,912^**^ | ,908^**^ | ,905^**^ | ,917^**^ | ,924^**^ | ,948^**^ | ,931^**^ |
| Day 25 (AM) |  |  |  |  |  |  | 1 | ,909^**^ | ,933^**^ | ,918^**^ | ,941^**^ | ,936^**^ | ,938^**^ | ,888^**^ |
| Day 25 (PM) |  |  |  |  |  |  |  | 1 | ,933^**^ | ,914^**^ | ,900^**^ | ,914^**^ | ,919^**^ | ,915^**^ |
| Day 26 (AM) |  |  |  |  |  |  |  |  | 1 | ,962^**^ | ,944^**^ | ,941^**^ | ,935^**^ | ,901^**^ |
| Day 26 (PM) |  |  |  |  |  |  |  |  |  | 1 | ,946^**^ | ,941^**^ | ,931^**^ | ,902^**^ |
| Day 27 (AM) |  |  |  |  |  |  |  |  |  |  | 1 | ,944^**^ | ,939^**^ | ,903^**^ |
| Day 27 (PM) |  |  |  |  |  |  |  |  |  |  |  | 1 | ,964^**^ | ,927^**^ |
| Day 28 (AM) |  |  |  |  |  |  |  |  |  |  |  |  | 1 | ,920^**^ |
| Day 28 (PM) |  |  |  |  |  |  |  |  |  |  |  |  |  | 1 |

**p<0.05, **p<0.001*

**S6 Table A.** Correlations for Online News from Day 1 (AM) to Day 31 (PM) (Second-Level Agenda) - **Continued**

| **Days** | **Day 29** | | **Day 30** | | **Day 31** | |
| --- | --- | --- | --- | --- | --- | --- |
|  | **AM** | **PM** | **AM** | **PM** | **AM** | **PM** |
| Day 1 (AM) | ,937^**^ | ,915^**^ | ,920^**^ | ,752^**^ | ,698^**^ | ,705^**^ |
| Day 1 (PM) | ,919^**^ | ,919^**^ | ,890^**^ | ,773^**^ | ,660^**^ | ,668^**^ |
| Day 2 (AM) | ,911^**^ | ,897^**^ | ,930^**^ | ,773^**^ | ,673^**^ | ,682^**^ |
| Day 2 (PM) | ,904^**^ | ,910^**^ | ,875^**^ | ,777^**^ | ,684^**^ | ,699^**^ |
| Day 3 (AM) | ,857^**^ | ,828^**^ | ,797^**^ | ,716^**^ | ,650^**^ | ,666^**^ |
| Day 3 (PM) | ,902^**^ | ,912^**^ | ,880^**^ | ,828^**^ | ,745^**^ | ,772^**^ |
| Day 4 (AM) | ,886^**^ | ,892^**^ | ,870^**^ | ,741^**^ | ,678^**^ | ,699^**^ |
| Day 4 (PM) | ,903^**^ | ,914^**^ | ,882^**^ | ,750^**^ | ,696^**^ | ,716^**^ |
| Day 5 (AM) | ,862^**^ | ,895^**^ | ,832^**^ | ,713^**^ | ,699^**^ | ,692^**^ |
| Day 5 (PM) | ,879^**^ | ,864^**^ | ,862^**^ | ,735^**^ | ,726^**^ | ,716^**^ |
| Day 6 (AM) | ,898^**^ | ,870^**^ | ,906^**^ | ,779^**^ | ,825^**^ | ,830^**^ |
| Day 6 (PM) | ,941^**^ | ,920^**^ | ,897^**^ | ,739^**^ | ,712^**^ | ,721^**^ |
| Day 7 (AM) | ,896^**^ | ,881^**^ | ,876^**^ | ,704^**^ | ,692^**^ | ,678^**^ |
| Day 7 (PM) | ,908^**^ | ,911^**^ | ,892^**^ | ,816^**^ | ,732^**^ | ,760^**^ |
| Day 8 (AM) | ,899^**^ | ,897^**^ | ,915^**^ | ,878^**^ | ,745^**^ | ,747^**^ |
| Day 8 (PM) | ,566^**^ | ,601^**^ | ,536^**^ | ,529^**^ | ,493^**^ | ,503^**^ |
| Day 9 (AM) | ,769^**^ | ,782^**^ | ,722^**^ | ,642^**^ | ,637^**^ | ,656^**^ |
| Day 9 (PM) | ,888^**^ | ,893^**^ | ,841^**^ | ,779^**^ | ,730^**^ | ,747^**^ |
| Day 10 (AM) | ,877^**^ | ,854^**^ | ,883^**^ | ,743^**^ | ,711^**^ | ,675^**^ |
| Day 10 (PM) | ,887^**^ | ,879^**^ | ,851^**^ | ,755^**^ | ,711^**^ | ,740^**^ |
| Day 11 (AM) | ,909^**^ | ,913^**^ | ,893^**^ | ,791^**^ | ,785^**^ | ,804^**^ |
| Day 11 (PM) | ,915^**^ | ,893^**^ | ,885^**^ | ,789^**^ | ,711^**^ | ,711^**^ |
| Day 12 (AM) | ,921^**^ | ,891^**^ | ,905^**^ | ,837^**^ | ,724^**^ | ,709^**^ |
| Day 12 (PM) | ,924^**^ | ,938^**^ | ,924^**^ | ,817^**^ | ,785^**^ | ,791^**^ |
| Day 13 (AM) | ,841^**^ | ,859^**^ | ,819^**^ | ,744^**^ | ,653^**^ | ,649^**^ |
| Day 13 (PM) | ,940^**^ | ,896^**^ | ,908^**^ | ,758^**^ | ,769^**^ | ,780^**^ |
| Day 14 (AM) | ,875^**^ | ,904^**^ | ,881^**^ | ,708^**^ | ,616^**^ | ,632^**^ |
| Day 14 (PM) | ,894^**^ | ,908^**^ | ,912^**^ | ,761^**^ | ,703^**^ | ,695^**^ |
| Day 15 (AM) | ,918^**^ | ,928^**^ | ,883^**^ | ,731^**^ | ,680^**^ | ,688^**^ |
| Day 15 (PM) | ,917^**^ | ,921^**^ | ,932^**^ | ,841^**^ | ,713^**^ | ,718^**^ |
| Day 16 (AM) | ,941^**^ | ,944^**^ | ,905^**^ | ,839^**^ | ,748^**^ | ,753^**^ |
| Day 16 (PM) | ,922^**^ | ,907^**^ | ,905^**^ | ,789^**^ | ,727^**^ | ,753^**^ |
| Day 17 (AM) | ,939^**^ | ,946^**^ | ,904^**^ | ,761^**^ | ,717^**^ | ,748^**^ |
| Day 17 (PM) | ,937^**^ | ,932^**^ | ,879^**^ | ,769^**^ | ,723^**^ | ,749^**^ |
| Day 18 (AM) | ,937^**^ | ,899^**^ | ,878^**^ | ,717^**^ | ,624^**^ | ,640^**^ |
| Day 18 (PM) | ,904^**^ | ,897^**^ | ,905^**^ | ,811^**^ | ,741^**^ | ,761^**^ |
| Day 19 (AM) | ,780^**^ | ,805^**^ | ,788^**^ | ,796^**^ | ,667^**^ | ,663^**^ |
| Day 19 (PM) | ,915^**^ | ,919^**^ | ,883^**^ | ,774^**^ | ,754^**^ | ,756^**^ |
| Day 20 (AM) | ,894^**^ | ,869^**^ | ,872^**^ | ,721^**^ | ,691^**^ | ,673^**^ |
| Day 20 (PM) | ,949^**^ | ,927^**^ | ,951^**^ | ,786^**^ | ,774^**^ | ,770^**^ |
| Day 21 (AM) | ,945^**^ | ,938^**^ | ,952^**^ | ,795^**^ | ,802^**^ | ,805^**^ |
| Day 21 (PM) | ,904^**^ | ,917^**^ | ,908^**^ | ,865^**^ | ,838^**^ | ,827^**^ |
| Day 22 (AM) | ,906^**^ | ,901^**^ | ,918^**^ | ,759^**^ | ,708^**^ | ,689^**^ |
| Day 22 (PM) | ,916^**^ | ,923^**^ | ,909^**^ | ,775^**^ | ,713^**^ | ,699^**^ |
| Day 23 (AM) | ,901^**^ | ,904^**^ | ,891^**^ | ,817^**^ | ,703^**^ | ,696^**^ |
| Day 23 (PM) | ,916^**^ | ,962^**^ | ,881^**^ | ,810^**^ | ,738^**^ | ,753^**^ |
| Day 24 (AM) | ,867^**^ | ,917^**^ | ,847^**^ | ,711^**^ | ,697^**^ | ,676^**^ |
| Day 24 (PM) | ,928^**^ | ,949^**^ | ,884^**^ | ,780^**^ | ,756^**^ | ,765^**^ |
| Day 25 (AM) | ,933^**^ | ,935^**^ | ,915^**^ | ,801^**^ | ,806^**^ | ,804^**^ |
| Day 25 (PM) | ,937^**^ | ,927^**^ | ,936^**^ | ,780^**^ | ,700^**^ | ,703^**^ |
| Day 26 (AM) | ,930^**^ | ,911^**^ | ,950^**^ | ,773^**^ | ,789^**^ | ,769^**^ |
| Day 26 (PM) | ,929^**^ | ,911^**^ | ,934^**^ | ,764^**^ | ,774^**^ | ,765^**^ |
| Day 27 (AM) | ,932^**^ | ,933^**^ | ,917^**^ | ,783^**^ | ,817^**^ | ,816^**^ |
| Day 27 (PM) | ,946^**^ | ,952^**^ | ,934^**^ | ,839^**^ | ,759^**^ | ,745^**^ |
| Day 28 (AM) | ,936^**^ | ,953^**^ | ,915^**^ | ,792^**^ | ,766^**^ | ,766^**^ |
| Day 28 (PM) | ,948^**^ | ,934^**^ | ,905^**^ | ,791^**^ | ,735^**^ | ,734^**^ |
| Day 29 (AM) | 1 | ,954^**^ | ,935^**^ | ,785^**^ | ,745^**^ | ,752^**^ |
| Day 29 (PM) |  | 1 | ,920^**^ | ,809^**^ | ,742^**^ | ,750^**^ |
| Day 30 (AM) |  |  | 1 | ,814^**^ | ,766^**^ | ,765^**^ |
| Day 30 (PM) |  |  |  | 1 | ,718^**^ | ,722^**^ |
| Day 31 (AM) |  |  |  |  | 1 | ,971^**^ |
| Day 31 (PM) |  |  |  |  |  | 1 |

**p<0.05, **p<0.001*

**S6 Table B.** Correlations for X from Day 1 (AM) to Day 31 (PM) (Second-Level Agenda)

| **Days** | **Day 1** | | **Day 2** | | **Day 3** | | **Day 4** | | **Day 5** | | **Day 6** | | **Day 7** | |
| --- | --- | --- | --- | --- | --- | --- | --- | --- | --- | --- | --- | --- | --- | --- |
|  | **AM** | **PM** | **AM** | **PM** | **AM** | **PM** | **AM** | **PM** | **AM** | **PM** | **AM** | **PM** | **AM** | **PM** |
| Day 1 (AM) | 1 | ,974^**^ | ,979^**^ | ,917^**^ | ,964^**^ | ,948^**^ | ,986^**^ | ,975^**^ | ,975^**^ | ,987^**^ | ,975^**^ | ,985^**^ | ,984^**^ | ,969^**^ |
| Day 1 (PM) |  | 1 | ,967^**^ | ,973^**^ | ,988^**^ | ,980^**^ | ,983^**^ | ,950^**^ | ,972^**^ | ,980^**^ | ,968^**^ | ,962^**^ | ,984^**^ | ,961^**^ |
| Day 2 (AM) |  |  | 1 | ,915^**^ | ,963^**^ | ,935^**^ | ,975^**^ | ,956^**^ | ,975^**^ | ,977^**^ | ,975^**^ | ,956^**^ | ,978^**^ | ,937^**^ |
| Day 2 (PM) |  |  |  | 1 | ,973^**^ | ,975^**^ | ,949^**^ | ,892^**^ | ,934^**^ | ,943^**^ | ,924^**^ | ,907^**^ | ,956^**^ | ,924^**^ |
| Day 3 (AM) |  |  |  |  | 1 | ,986^**^ | ,973^**^ | ,939^**^ | ,968^**^ | ,978^**^ | ,973^**^ | ,948^**^ | ,980^**^ | ,948^**^ |
| Day 3 (PM) |  |  |  |  |  | 1 | ,959^**^ | ,913^**^ | ,945^**^ | ,969^**^ | ,964^**^ | ,948^**^ | ,963^**^ | ,957^**^ |
| Day 4 (AM) |  |  |  |  |  |  | 1 | ,973^**^ | ,980^**^ | ,979^**^ | ,962^**^ | ,968^**^ | ,991^**^ | ,963^**^ |
| Day 4 (PM) |  |  |  |  |  |  |  | 1 | ,983^**^ | ,962^**^ | ,938^**^ | ,957^**^ | ,966^**^ | ,942^**^ |
| Day 5 (AM) |  |  |  |  |  |  |  |  | 1 | ,976^**^ | ,965^**^ | ,958^**^ | ,981^**^ | ,947^**^ |
| Day 5 (PM) |  |  |  |  |  |  |  |  |  | 1 | ,986^**^ | ,982^**^ | ,985^**^ | ,978^**^ |
| Day 6 (AM) |  |  |  |  |  |  |  |  |  |  | 1 | ,968^**^ | ,968^**^ | ,958^**^ |
| Day 6 (PM) |  |  |  |  |  |  |  |  |  |  |  | 1 | ,968^**^ | ,981^**^ |
| Day 7 (AM) |  |  |  |  |  |  |  |  |  |  |  |  | 1 | ,966^**^ |
| Day 7 (PM) |  |  |  |  |  |  |  |  |  |  |  |  |  | 1 |

**p<0.05, **p<0.001*

**S6 Table B.** Correlations for X from Day 1 (AM) to Day 31 (PM) (Second-Level Agenda) - **Continued**

| **Days** | **Day 8** | | **Day 9** | | **Day 10** | | **Day 11** | | **Day 12** | | **Day 13** | | **Day 14** | |
| --- | --- | --- | --- | --- | --- | --- | --- | --- | --- | --- | --- | --- | --- | --- |
|  | **AM** | **PM** | **AM** | **PM** | **AM** | **PM** | **AM** | **PM** | **AM** | **PM** | **AM** | **PM** | **AM** | **PM** |
| Day 1 (AM) | ,857^**^ | ,790^**^ | ,772^**^ | ,767^**^ | ,873^**^ | ,836^**^ | ,794^**^ | ,685^**^ | ,885^**^ | ,872^**^ | ,935^**^ | ,880^**^ | ,861^**^ | ,852^**^ |
| Day 1 (PM) | ,872^**^ | ,829^**^ | ,849^**^ | ,856^**^ | ,914^**^ | ,856^**^ | ,811^**^ | ,693^**^ | ,896^**^ | ,873^**^ | ,928^**^ | ,878^**^ | ,862^**^ | ,869^**^ |
| Day 2 (AM) | ,807^**^ | ,746^**^ | ,743^**^ | ,742^**^ | ,840^**^ | ,801^**^ | ,755^**^ | ,654^**^ | ,845^**^ | ,832^**^ | ,904^**^ | ,837^**^ | ,824^**^ | ,830^**^ |
| Day 2 (PM) | ,872^**^ | ,858^**^ | ,913^**^ | ,930^**^ | ,921^**^ | ,856^**^ | ,785^**^ | ,680^**^ | ,872^**^ | ,836^**^ | ,894^**^ | ,846^**^ | ,835^**^ | ,854^**^ |
| Day 3 (AM) | ,862^**^ | ,822^**^ | ,840^**^ | ,849^**^ | ,922^**^ | ,868^**^ | ,825^**^ | ,699^**^ | ,901^**^ | ,874^**^ | ,931^**^ | ,880^**^ | ,879^**^ | ,885^**^ |
| Day 3 (PM) | ,888^**^ | ,851^**^ | ,857^**^ | ,878^**^ | ,938^**^ | ,897^**^ | ,852^**^ | ,723^**^ | ,926^**^ | ,905^**^ | ,945^**^ | ,901^**^ | ,885^**^ | ,899^**^ |
| Day 4 (AM) | ,846^**^ | ,797^**^ | ,814^**^ | ,814^**^ | ,875^**^ | ,822^**^ | ,771^**^ | ,664^**^ | ,865^**^ | ,848^**^ | ,915^**^ | ,853^**^ | ,834^**^ | ,834^**^ |
| Day 4 (PM) | ,817^**^ | ,755^**^ | ,754^**^ | ,746^**^ | ,827^**^ | ,791^**^ | ,754^**^ | ,633^**^ | ,827^**^ | ,841^**^ | ,898^**^ | ,835^**^ | ,842^**^ | ,835^**^ |
| Day 5 (AM) | ,839^**^ | ,790^**^ | ,801^**^ | ,797^**^ | ,862^**^ | ,817^**^ | ,773^**^ | ,663^**^ | ,850^**^ | ,862^**^ | ,916^**^ | ,847^**^ | ,861^**^ | ,866^**^ |
| Day 5 (PM) | ,895^**^ | ,839^**^ | ,804^**^ | ,812^**^ | ,901^**^ | ,870^**^ | ,824^**^ | ,709^**^ | ,905^**^ | ,891^**^ | ,953^**^ | ,900^**^ | ,884^**^ | ,888^**^ |
| Day 6 (AM) | ,867^**^ | ,806^**^ | ,774^**^ | ,781^**^ | ,900^**^ | ,873^**^ | ,836^**^ | ,717^**^ | ,917^**^ | ,909^**^ | ,958^**^ | ,901^**^ | ,893^**^ | ,903^**^ |
| Day 6 (PM) | ,889^**^ | ,817^**^ | ,781^**^ | ,783^**^ | ,887^**^ | ,860^**^ | ,830^**^ | ,716^**^ | ,908^**^ | ,902^**^ | ,952^**^ | ,913^**^ | ,885^**^ | ,876^**^ |
| Day 7 (AM) | ,870^**^ | ,823^**^ | ,835^**^ | ,831^**^ | ,892^**^ | ,837^**^ | ,777^**^ | ,669^**^ | ,873^**^ | ,849^**^ | ,924^**^ | ,863^**^ | ,856^**^ | ,852^**^ |
| Day 7 (PM) | ,922^**^ | ,866^**^ | ,822^**^ | ,832^**^ | ,906^**^ | ,888^**^ | ,850^**^ | ,718^**^ | ,919^**^ | ,910^**^ | ,963^**^ | ,919^**^ | ,887^**^ | ,886^**^ |
| Day 8 (AM) | 1 | ,977^**^ | ,872^**^ | ,879^**^ | ,926^**^ | ,884^**^ | ,844^**^ | ,722^**^ | ,907^**^ | ,872^**^ | ,923^**^ | ,903^**^ | ,881^**^ | ,881^**^ |
| Day 8 (PM) |  | 1 | ,901^**^ | ,906^**^ | ,906^**^ | ,847^**^ | ,802^**^ | ,676^**^ | ,858^**^ | ,816^**^ | ,866^**^ | ,845^**^ | ,828^**^ | ,837^**^ |
| Day 9 (AM) |  |  | 1 | ,977^**^ | ,911^**^ | ,813^**^ | ,729^**^ | ,616^**^ | ,805^**^ | ,753^**^ | ,808^**^ | ,787^**^ | ,794^**^ | ,800^**^ |
| Day 9 (PM) |  |  |  | 1 | ,912^**^ | ,843^**^ | ,756^**^ | ,649^**^ | ,820^**^ | ,783^**^ | ,822^**^ | ,796^**^ | ,788^**^ | ,813^**^ |
| Day 10 (AM) |  |  |  |  | 1 | ,930^**^ | ,897^**^ | ,742^**^ | ,954^**^ | ,899^**^ | ,933^**^ | ,928^**^ | ,927^**^ | ,927^**^ |
| Day 10 (PM) |  |  |  |  |  | 1 | ,936^**^ | ,816^**^ | ,949^**^ | ,931^**^ | ,947^**^ | ,946^**^ | ,924^**^ | ,938^**^ |
| Day 11 (AM) |  |  |  |  |  |  | 1 | ,807^**^ | ,943^**^ | ,920^**^ | ,905^**^ | ,943^**^ | ,907^**^ | ,919^**^ |
| Day 11 (PM) |  |  |  |  |  |  |  | 1 | ,856^**^ | ,804^**^ | ,791^**^ | ,864^**^ | ,800^**^ | ,795^**^ |
| Day 12 (AM) |  |  |  |  |  |  |  |  | 1 | ,952^**^ | ,960^**^ | ,977^**^ | ,945^**^ | ,946^**^ |
| Day 12 (PM) |  |  |  |  |  |  |  |  |  | 1 | ,967^**^ | ,944^**^ | ,925^**^ | ,943^**^ |
| Day 13 (AM) |  |  |  |  |  |  |  |  |  |  | 1 | ,959^**^ | ,940^**^ | ,939^**^ |
| Day 13 (PM) |  |  |  |  |  |  |  |  |  |  |  | 1 | ,957^**^ | ,949^**^ |
| Day 14 (AM) |  |  |  |  |  |  |  |  |  |  |  |  | 1 | ,972^**^ |
| Day 14 (PM) |  |  |  |  |  |  |  |  |  |  |  |  |  | 1 |

**p<0.05, **p<0.001*

**S6 Table B.** Correlations for X from Day 1 (AM) to Day 31 (PM) (Second-Level Agenda) - **Continued**

| **Days** | **Day 15** | | **Day 16** | | **Day 17** | | **Day 18** | | **Day 19** | | **Day 20** | | **Day 21** | |
| --- | --- | --- | --- | --- | --- | --- | --- | --- | --- | --- | --- | --- | --- | --- |
|  | **AM** | **PM** | **AM** | **PM** | **AM** | **PM** | **AM** | **PM** | **AM** | **PM** | **AM** | **PM** | **AM** | **PM** |
| Day 1 (AM) | ,779^**^ | ,877^**^ | ,921^**^ | ,951^**^ | ,925^**^ | ,825^**^ | ,904^**^ | ,902^**^ | ,912^**^ | ,868^**^ | ,944^**^ | ,904^**^ | ,903^**^ | ,865^**^ |
| Day 1 (PM) | ,793^**^ | ,875^**^ | ,914^**^ | ,942^**^ | ,924^**^ | ,833^**^ | ,904^**^ | ,896^**^ | ,918^**^ | ,873^**^ | ,939^**^ | ,887^**^ | ,898^**^ | ,872^**^ |
| Day 2 (AM) | ,738^**^ | ,843^**^ | ,878^**^ | ,918^**^ | ,891^**^ | ,796^**^ | ,877^**^ | ,871^**^ | ,886^**^ | ,835^**^ | ,926^**^ | ,840^**^ | ,860^**^ | ,815^**^ |
| Day 2 (PM) | ,775^**^ | ,850^**^ | ,877^**^ | ,901^**^ | ,897^**^ | ,819^**^ | ,869^**^ | ,861^**^ | ,888^**^ | ,843^**^ | ,900^**^ | ,846^**^ | ,861^**^ | ,834^**^ |
| Day 3 (AM) | ,811^**^ | ,873^**^ | ,906^**^ | ,929^**^ | ,923^**^ | ,840^**^ | ,909^**^ | ,896^**^ | ,928^**^ | ,887^**^ | ,943^**^ | ,877^**^ | ,902^**^ | ,869^**^ |
| Day 3 (PM) | ,836^**^ | ,891^**^ | ,914^**^ | ,933^**^ | ,937^**^ | ,873^**^ | ,924^**^ | ,911^**^ | ,933^**^ | ,903^**^ | ,939^**^ | ,896^**^ | ,912^**^ | ,895^**^ |
| Day 4 (AM) | ,745^**^ | ,856^**^ | ,899^**^ | ,939^**^ | ,908^**^ | ,801^**^ | ,887^**^ | ,888^**^ | ,894^**^ | ,847^**^ | ,933^**^ | ,879^**^ | ,884^**^ | ,849^**^ |
| Day 4 (PM) | ,745^**^ | ,845^**^ | ,891^**^ | ,930^**^ | ,889^**^ | ,774^**^ | ,853^**^ | ,877^**^ | ,887^**^ | ,823^**^ | ,939^**^ | ,874^**^ | ,881^**^ | ,850^**^ |
| Day 5 (AM) | ,772^**^ | ,864^**^ | ,898^**^ | ,931^**^ | ,908^**^ | ,802^**^ | ,873^**^ | ,883^**^ | ,907^**^ | ,843^**^ | ,949^**^ | ,864^**^ | ,885^**^ | ,851^**^ |
| Day 5 (PM) | ,814^**^ | ,905^**^ | ,931^**^ | ,962^**^ | ,946^**^ | ,862^**^ | ,920^**^ | ,920^**^ | ,937^**^ | ,896^**^ | ,963^**^ | ,914^**^ | ,920^**^ | ,887^**^ |
| Day 6 (AM) | ,832^**^ | ,903^**^ | ,923^**^ | ,947^**^ | ,946^**^ | ,864^**^ | ,932^**^ | ,922^**^ | ,944^**^ | ,910^**^ | ,956^**^ | ,900^**^ | ,917^**^ | ,882^**^ |
| Day 6 (PM) | ,819^**^ | ,906^**^ | ,946^**^ | ,963^**^ | ,951^**^ | ,855^**^ | ,923^**^ | ,920^**^ | ,932^**^ | ,902^**^ | ,963^**^ | ,941^**^ | ,928^**^ | ,896^**^ |
| Day 7 (AM) | ,769^**^ | ,869^**^ | ,910^**^ | ,941^**^ | ,920^**^ | ,821^**^ | ,891^**^ | ,889^**^ | ,907^**^ | ,857^**^ | ,938^**^ | ,881^**^ | ,891^**^ | ,849^**^ |
| Day 7 (PM) | ,825^**^ | ,925^**^ | ,951^**^ | ,975^**^ | ,960^**^ | ,885^**^ | ,928^**^ | ,938^**^ | ,936^**^ | ,912^**^ | ,957^**^ | ,945^**^ | ,935^**^ | ,923^**^ |
| Day 8 (AM) | ,854^**^ | ,936^**^ | ,927^**^ | ,925^**^ | ,940^**^ | ,898^**^ | ,878^**^ | ,893^**^ | ,920^**^ | ,890^**^ | ,885^**^ | ,915^**^ | ,899^**^ | ,878^**^ |
| Day 8 (PM) | ,808^**^ | ,892^**^ | ,858^**^ | ,866^**^ | ,882^**^ | ,850^**^ | ,817^**^ | ,838^**^ | ,864^**^ | ,833^**^ | ,817^**^ | ,844^**^ | ,833^**^ | ,823^**^ |
| Day 9 (AM) | ,760^**^ | ,816^**^ | ,832^**^ | ,826^**^ | ,841^**^ | ,759^**^ | ,774^**^ | ,792^**^ | ,820^**^ | ,771^**^ | ,784^**^ | ,789^**^ | ,790^**^ | ,769^**^ |
| Day 9 (PM) | ,771^**^ | ,823^**^ | ,821^**^ | ,825^**^ | ,843^**^ | ,790^**^ | ,786^**^ | ,799^**^ | ,822^**^ | ,789^**^ | ,799^**^ | ,794^**^ | ,797^**^ | ,787^**^ |
| Day 10 (AM) | ,914^**^ | ,922^**^ | ,935^**^ | ,914^**^ | ,949^**^ | ,895^**^ | ,928^**^ | ,919^**^ | ,951^**^ | ,933^**^ | ,906^**^ | ,909^**^ | ,931^**^ | ,901^**^ |
| Day 10 (PM) | ,923^**^ | ,920^**^ | ,905^**^ | ,897^**^ | ,928^**^ | ,891^**^ | ,906^**^ | ,922^**^ | ,937^**^ | ,933^**^ | ,904^**^ | ,902^**^ | ,919^**^ | ,917^**^ |
| Day 11 (AM) | ,907^**^ | ,892^**^ | ,877^**^ | ,862^**^ | ,902^**^ | ,860^**^ | ,891^**^ | ,896^**^ | ,926^**^ | ,932^**^ | ,887^**^ | ,896^**^ | ,920^**^ | ,927^**^ |
| Day 11 (PM) | ,756^**^ | ,776^**^ | ,742^**^ | ,737^**^ | ,764^**^ | ,727^**^ | ,746^**^ | ,753^**^ | ,781^**^ | ,789^**^ | ,743^**^ | ,752^**^ | ,751^**^ | ,742^**^ |
| Day 12 (AM) | ,923^**^ | ,937^**^ | ,938^**^ | ,928^**^ | ,957^**^ | ,915^**^ | ,948^**^ | ,941^**^ | ,960^**^ | ,967^**^ | ,922^**^ | ,940^**^ | ,952^**^ | ,931^**^ |
| Day 12 (PM) | ,910^**^ | ,934^**^ | ,921^**^ | ,911^**^ | ,940^**^ | ,907^**^ | ,915^**^ | ,929^**^ | ,939^**^ | ,939^**^ | ,926^**^ | ,916^**^ | ,922^**^ | ,924^**^ |
| Day 13 (AM) | ,896^**^ | ,958^**^ | ,960^**^ | ,963^**^ | ,974^**^ | ,908^**^ | ,940^**^ | ,955^**^ | ,970^**^ | ,953^**^ | ,958^**^ | ,944^**^ | ,944^**^ | ,933^**^ |
| Day 13 (PM) | ,921^**^ | ,951^**^ | ,951^**^ | ,934^**^ | ,961^**^ | ,910^**^ | ,938^**^ | ,946^**^ | ,961^**^ | ,960^**^ | ,935^**^ | ,949^**^ | ,950^**^ | ,927^**^ |
| Day 14 (AM) | ,952^**^ | ,937^**^ | ,940^**^ | ,907^**^ | ,949^**^ | ,883^**^ | ,911^**^ | ,926^**^ | ,967^**^ | ,939^**^ | ,933^**^ | ,919^**^ | ,948^**^ | ,913^**^ |
| Day 14 (PM) | ,966^**^ | ,951^**^ | ,932^**^ | ,908^**^ | ,953^**^ | ,927^**^ | ,929^**^ | ,945^**^ | ,969^**^ | ,944^**^ | ,944^**^ | ,908^**^ | ,944^**^ | ,919^**^ |
| Day 15 (AM) | 1 | ,923^**^ | ,906^**^ | ,848^**^ | ,916^**^ | ,914^**^ | ,903^**^ | ,909^**^ | ,934^**^ | ,933^**^ | ,890^**^ | ,882^**^ | ,912^**^ | ,893^**^ |
| Day 15 (PM) |  | 1 | ,966^**^ | ,946^**^ | ,973^**^ | ,936^**^ | ,928^**^ | ,967^**^ | ,963^**^ | ,935^**^ | ,936^**^ | ,933^**^ | ,932^**^ | ,917^**^ |
| Day 16 (AM) |  |  | 1 | ,968^**^ | ,984^**^ | ,916^**^ | ,942^**^ | ,962^**^ | ,972^**^ | ,939^**^ | ,955^**^ | ,960^**^ | ,956^**^ | ,927^**^ |
| Day 16 (PM) |  |  |  | 1 | ,976^**^ | ,894^**^ | ,930^**^ | ,956^**^ | ,956^**^ | ,923^**^ | ,959^**^ | ,963^**^ | ,949^**^ | ,932^**^ |
| Day 17 (AM) |  |  |  |  | 1 | ,930^**^ | ,960^**^ | ,971^**^ | ,982^**^ | ,950^**^ | ,963^**^ | ,963^**^ | ,964^**^ | ,936^**^ |
| Day 17 (PM) |  |  |  |  |  | 1 | ,933^**^ | ,933^**^ | ,909^**^ | ,923^**^ | ,896^**^ | ,887^**^ | ,893^**^ | ,878^**^ |
| Day 18 (AM) |  |  |  |  |  |  | 1 | ,969^**^ | ,949^**^ | ,957^**^ | ,942^**^ | ,929^**^ | ,940^**^ | ,922^**^ |
| Day 18 (PM) |  |  |  |  |  |  |  | 1 | ,964^**^ | ,952^**^ | ,957^**^ | ,948^**^ | ,954^**^ | ,939^**^ |
| Day 19 (AM) |  |  |  |  |  |  |  |  | 1 | ,958^**^ | ,969^**^ | ,946^**^ | ,970^**^ | ,944^**^ |
| Day 19 (PM) |  |  |  |  |  |  |  |  |  | 1 | ,944^**^ | ,948^**^ | ,957^**^ | ,941^**^ |
| Day 20 (AM) |  |  |  |  |  |  |  |  |  |  | 1 | ,947^**^ | ,961^**^ | ,935^**^ |
| Day 20 (PM) |  |  |  |  |  |  |  |  |  |  |  | 1 | ,974^**^ | ,952^**^ |
| Day 21 (AM) |  |  |  |  |  |  |  |  |  |  |  |  | 1 | ,966^**^ |
| Day 21 (PM) |  |  |  |  |  |  |  |  |  |  |  |  |  | 1 |

**p<0.05, **p<0.001*

**S6 Table B.** Correlations for X from Day 1 (AM) to Day 31 (PM) (Second-Level Agenda) - **Continued**

| **Days** | **Day 22** | | **Day 23** | | **Day 24** | | **Day 25** | | **Day 26** | | **Day 27** | | **Day 28** | |
| --- | --- | --- | --- | --- | --- | --- | --- | --- | --- | --- | --- | --- | --- | --- |
|  | **AM** | **PM** | **AM** | **PM** | **AM** | **PM** | **AM** | **PM** | **AM** | **PM** | **AM** | **PM** | **AM** | **PM** |
| Day 1 (AM) | ,925^**^ | ,773^**^ | ,721^**^ | ,893^**^ | ,919^**^ | ,917^**^ | ,958^**^ | ,906^**^ | ,857^**^ | ,843^**^ | ,867^**^ | ,856^**^ | ,868^**^ | ,893^**^ |
| Day 1 (PM) | ,918^**^ | ,775^**^ | ,714^**^ | ,874^**^ | ,913^**^ | ,896^**^ | ,951^**^ | ,909^**^ | ,847^**^ | ,857^**^ | ,872^**^ | ,867^**^ | ,875^**^ | ,899^**^ |
| Day 2 (AM) | ,878^**^ | ,751^**^ | ,700^**^ | ,866^**^ | ,883^**^ | ,883^**^ | ,933^**^ | ,866^**^ | ,802^**^ | ,795^**^ | ,816^**^ | ,804^**^ | ,824^**^ | ,864^**^ |
| Day 2 (PM) | ,873^**^ | ,757^**^ | ,694^**^ | ,842^**^ | ,879^**^ | ,857^**^ | ,913^**^ | ,885^**^ | ,816^**^ | ,844^**^ | ,843^**^ | ,843^**^ | ,853^**^ | ,870^**^ |
| Day 3 (AM) | ,906^**^ | ,773^**^ | ,708^**^ | ,869^**^ | ,911^**^ | ,888^**^ | ,945^**^ | ,908^**^ | ,845^**^ | ,861^**^ | ,867^**^ | ,863^**^ | ,874^**^ | ,897^**^ |
| Day 3 (PM) | ,919^**^ | ,784^**^ | ,714^**^ | ,859^**^ | ,918^**^ | ,881^**^ | ,945^**^ | ,926^**^ | ,872^**^ | ,889^**^ | ,894^**^ | ,900^**^ | ,906^**^ | ,914^**^ |
| Day 4 (AM) | ,906^**^ | ,772^**^ | ,719^**^ | ,882^**^ | ,906^**^ | ,903^**^ | ,949^**^ | ,891^**^ | ,828^**^ | ,831^**^ | ,850^**^ | ,838^**^ | ,849^**^ | ,885^**^ |
| Day 4 (PM) | ,905^**^ | ,760^**^ | ,710^**^ | ,886^**^ | ,901^**^ | ,904^**^ | ,943^**^ | ,881^**^ | ,818^**^ | ,821^**^ | ,834^**^ | ,820^**^ | ,836^**^ | ,871^**^ |
| Day 5 (AM) | ,905^**^ | ,765^**^ | ,709^**^ | ,889^**^ | ,905^**^ | ,895^**^ | ,946^**^ | ,894^**^ | ,825^**^ | ,839^**^ | ,843^**^ | ,832^**^ | ,848^**^ | ,882^**^ |
| Day 5 (PM) | ,933^**^ | ,794^**^ | ,730^**^ | ,893^**^ | ,933^**^ | ,910^**^ | ,964^**^ | ,922^**^ | ,868^**^ | ,871^**^ | ,885^**^ | ,881^**^ | ,893^**^ | ,912^**^ |
| Day 6 (AM) | ,924^**^ | ,784^**^ | ,717^**^ | ,880^**^ | ,928^**^ | ,891^**^ | ,955^**^ | ,922^**^ | ,867^**^ | ,866^**^ | ,888^**^ | ,883^**^ | ,893^**^ | ,912^**^ |
| Day 6 (PM) | ,954^**^ | ,786^**^ | ,722^**^ | ,898^**^ | ,938^**^ | ,909^**^ | ,961^**^ | ,926^**^ | ,901^**^ | ,889^**^ | ,908^**^ | ,903^**^ | ,907^**^ | ,916^**^ |
| Day 7 (AM) | ,907^**^ | ,775^**^ | ,721^**^ | ,892^**^ | ,913^**^ | ,904^**^ | ,951^**^ | ,904^**^ | ,844^**^ | ,840^**^ | ,853^**^ | ,845^**^ | ,857^**^ | ,889^**^ |
| Day 7 (PM) | ,956^**^ | ,821^**^ | ,741^**^ | ,899^**^ | ,951^**^ | ,911^**^ | ,970^**^ | ,939^**^ | ,901^**^ | ,896^**^ | ,916^**^ | ,916^**^ | ,918^**^ | ,928^**^ |
| Day 8 (AM) | ,907^**^ | ,762^**^ | ,685^**^ | ,851^**^ | ,906^**^ | ,841^**^ | ,896^**^ | ,924^**^ | ,907^**^ | ,892^**^ | ,901^**^ | ,913^**^ | ,911^**^ | ,886^**^ |
| Day 8 (PM) | ,842^**^ | ,721^**^ | ,642^**^ | ,802^**^ | ,848^**^ | ,782^**^ | ,838^**^ | ,883^**^ | ,853^**^ | ,837^**^ | ,843^**^ | ,862^**^ | ,856^**^ | ,833^**^ |
| Day 9 (AM) | ,805^**^ | ,685^**^ | ,627^**^ | ,793^**^ | ,820^**^ | ,763^**^ | ,824^**^ | ,860^**^ | ,819^**^ | ,799^**^ | ,796^**^ | ,803^**^ | ,797^**^ | ,799^**^ |
| Day 9 (PM) | ,809^**^ | ,707^**^ | ,636^**^ | ,779^**^ | ,819^**^ | ,763^**^ | ,818^**^ | ,851^**^ | ,801^**^ | ,817^**^ | ,804^**^ | ,816^**^ | ,812^**^ | ,810^**^ |
| Day 10 (AM) | ,915^**^ | ,757^**^ | ,685^**^ | ,854^**^ | ,930^**^ | ,850^**^ | ,917^**^ | ,947^**^ | ,925^**^ | ,904^**^ | ,921^**^ | ,926^**^ | ,921^**^ | ,909^**^ |
| Day 10 (PM) | ,908^**^ | ,792^**^ | ,705^**^ | ,837^**^ | ,920^**^ | ,828^**^ | ,896^**^ | ,926^**^ | ,902^**^ | ,899^**^ | ,915^**^ | ,913^**^ | ,908^**^ | ,902^**^ |
| Day 11 (AM) | ,899^**^ | ,778^**^ | ,666^**^ | ,788^**^ | ,899^**^ | ,787^**^ | ,864^**^ | ,889^**^ | ,888^**^ | ,901^**^ | ,922^**^ | ,921^**^ | ,914^**^ | ,886^**^ |
| Day 11 (PM) | ,767^**^ | ,614^**^ | ,533^**^ | ,670^**^ | ,747^**^ | ,692^**^ | ,722^**^ | ,743^**^ | ,746^**^ | ,812^**^ | ,768^**^ | ,767^**^ | ,757^**^ | ,738^**^ |
| Day 12 (AM) | ,940^**^ | ,776^**^ | ,692^**^ | ,843^**^ | ,939^**^ | ,855^**^ | ,921^**^ | ,942^**^ | ,931^**^ | ,932^**^ | ,947^**^ | ,952^**^ | ,945^**^ | ,920^**^ |
| Day 12 (PM) | ,931^**^ | ,770^**^ | ,684^**^ | ,837^**^ | ,918^**^ | ,833^**^ | ,906^**^ | ,926^**^ | ,903^**^ | ,902^**^ | ,922^**^ | ,933^**^ | ,932^**^ | ,902^**^ |
| Day 13 (AM) | ,960^**^ | ,804^**^ | ,725^**^ | ,893^**^ | ,957^**^ | ,890^**^ | ,956^**^ | ,959^**^ | ,927^**^ | ,916^**^ | ,939^**^ | ,937^**^ | ,936^**^ | ,932^**^ |
| Day 13 (PM) | ,946^**^ | ,776^**^ | ,692^**^ | ,853^**^ | ,943^**^ | ,855^**^ | ,924^**^ | ,937^**^ | ,943^**^ | ,923^**^ | ,943^**^ | ,947^**^ | ,946^**^ | ,922^**^ |
| Day 14 (AM) | ,929^**^ | ,742^**^ | ,670^**^ | ,865^**^ | ,943^**^ | ,854^**^ | ,917^**^ | ,942^**^ | ,938^**^ | ,926^**^ | ,922^**^ | ,925^**^ | ,932^**^ | ,916^**^ |
| Day 14 (PM) | ,919^**^ | ,762^**^ | ,687^**^ | ,846^**^ | ,936^**^ | ,835^**^ | ,915^**^ | ,937^**^ | ,923^**^ | ,927^**^ | ,925^**^ | ,933^**^ | ,944^**^ | ,924^**^ |
| Day 15 (AM) | ,882^**^ | ,709^**^ | ,633^**^ | ,801^**^ | ,898^**^ | ,783^**^ | ,855^**^ | ,915^**^ | ,931^**^ | ,904^**^ | ,902^**^ | ,916^**^ | ,916^**^ | ,885^**^ |
| Day 15 (PM) | ,940^**^ | ,781^**^ | ,699^**^ | ,869^**^ | ,946^**^ | ,861^**^ | ,935^**^ | ,949^**^ | ,945^**^ | ,915^**^ | ,932^**^ | ,942^**^ | ,950^**^ | ,934^**^ |
| Day 16 (AM) | ,963^**^ | ,789^**^ | ,718^**^ | ,898^**^ | ,968^**^ | ,896^**^ | ,961^**^ | ,957^**^ | ,958^**^ | ,922^**^ | ,946^**^ | ,945^**^ | ,953^**^ | ,942^**^ |
| Day 16 (PM) | ,967^**^ | ,824^**^ | ,747^**^ | ,908^**^ | ,964^**^ | ,914^**^ | ,974^**^ | ,958^**^ | ,924^**^ | ,908^**^ | ,930^**^ | ,930^**^ | ,934^**^ | ,938^**^ |
| Day 17 (AM) | ,965^**^ | ,809^**^ | ,730^**^ | ,900^**^ | ,973^**^ | ,892^**^ | ,962^**^ | ,969^**^ | ,959^**^ | ,937^**^ | ,951^**^ | ,957^**^ | ,961^**^ | ,951^**^ |
| Day 17 (PM) | ,874^**^ | ,744^**^ | ,669^**^ | ,805^**^ | ,891^**^ | ,808^**^ | ,875^**^ | ,899^**^ | ,898^**^ | ,867^**^ | ,879^**^ | ,910^**^ | ,931^**^ | ,887^**^ |
| Day 18 (AM) | ,930^**^ | ,778^**^ | ,698^**^ | ,846^**^ | ,936^**^ | ,859^**^ | ,934^**^ | ,934^**^ | ,920^**^ | ,903^**^ | ,935^**^ | ,938^**^ | ,939^**^ | ,926^**^ |
| Day 18 (PM) | ,948^**^ | ,805^**^ | ,725^**^ | ,876^**^ | ,962^**^ | ,884^**^ | ,960^**^ | ,953^**^ | ,937^**^ | ,917^**^ | ,942^**^ | ,947^**^ | ,955^**^ | ,950^**^ |
| Day 19 (AM) | ,961^**^ | ,797^**^ | ,719^**^ | ,889^**^ | ,970^**^ | ,887^**^ | ,960^**^ | ,961^**^ | ,945^**^ | ,945^**^ | ,955^**^ | ,951^**^ | ,954^**^ | ,949^**^ |
| Day 19 (PM) | ,938^**^ | ,781^**^ | ,688^**^ | ,843^**^ | ,940^**^ | ,841^**^ | ,916^**^ | ,937^**^ | ,932^**^ | ,925^**^ | ,953^**^ | ,952^**^ | ,943^**^ | ,917^**^ |
| Day 20 (AM) | ,961^**^ | ,809^**^ | ,730^**^ | ,895^**^ | ,960^**^ | ,899^**^ | ,966^**^ | ,939^**^ | ,912^**^ | ,927^**^ | ,935^**^ | ,928^**^ | ,939^**^ | ,944^**^ |
| Day 20 (PM) | ,974^**^ | ,803^**^ | ,721^**^ | ,876^**^ | ,957^**^ | ,885^**^ | ,945^**^ | ,944^**^ | ,947^**^ | ,943^**^ | ,966^**^ | ,964^**^ | ,953^**^ | ,930^**^ |
| Day 21 (AM) | ,965^**^ | ,804^**^ | ,724^**^ | ,876^**^ | ,964^**^ | ,885^**^ | ,951^**^ | ,943^**^ | ,937^**^ | ,942^**^ | ,961^**^ | ,954^**^ | ,949^**^ | ,937^**^ |
| Day 21 (PM) | ,965^**^ | ,818^**^ | ,712^**^ | ,844^**^ | ,943^**^ | ,859^**^ | ,935^**^ | ,939^**^ | ,918^**^ | ,939^**^ | ,961^**^ | ,957^**^ | ,939^**^ | ,926^**^ |
| Day 22 (AM) | 1 | ,809^**^ | ,721^**^ | ,897^**^ | ,967^**^ | ,914^**^ | ,966^**^ | ,962^**^ | ,948^**^ | ,952^**^ | ,971^**^ | ,964^**^ | ,955^**^ | ,947^**^ |
| Day 22 (PM) |  | 1 | ,965^**^ | ,907^**^ | ,875^**^ | ,849^**^ | ,829^**^ | ,804^**^ | ,764^**^ | ,777^**^ | ,794^**^ | ,789^**^ | ,785^**^ | ,801^**^ |
| Day 23 (AM) |  |  | 1 | ,887^**^ | ,812^**^ | ,812^**^ | ,763^**^ | ,728^**^ | ,693^**^ | ,690^**^ | ,711^**^ | ,705^**^ | ,703^**^ | ,717^**^ |
| Day 23 (PM) |  |  |  | 1 | ,946^**^ | ,958^**^ | ,925^**^ | ,914^**^ | ,882^**^ | ,845^**^ | ,854^**^ | ,847^**^ | ,854^**^ | ,873^**^ |
| Day 24 (AM) |  |  |  |  | 1 | ,930^**^ | ,973^**^ | ,962^**^ | ,947^**^ | ,937^**^ | ,956^**^ | ,951^**^ | ,952^**^ | ,951^**^ |
| Day 24 (PM) |  |  |  |  |  | 1 | ,943^**^ | ,913^**^ | ,869^**^ | ,855^**^ | ,862^**^ | ,857^**^ | ,869^**^ | ,889^**^ |
| Day 25 (AM) |  |  |  |  |  |  | 1 | ,962^**^ | ,924^**^ | ,914^**^ | ,938^**^ | ,932^**^ | ,939^**^ | ,948^**^ |
| Day 25 (PM) |  |  |  |  |  |  |  | 1 | ,968^**^ | ,933^**^ | ,949^**^ | ,955^**^ | ,948^**^ | ,941^**^ |
| Day 26 (AM) |  |  |  |  |  |  |  |  | 1 | ,937^**^ | ,951^**^ | ,963^**^ | ,955^**^ | ,926^**^ |
| Day 26 (PM) |  |  |  |  |  |  |  |  |  | 1 | ,967^**^ | ,965^**^ | ,951^**^ | ,924^**^ |
| Day 27 (AM) |  |  |  |  |  |  |  |  |  |  | 1 | ,986^**^ | ,967^**^ | ,935^**^ |
| Day 27 (PM) |  |  |  |  |  |  |  |  |  |  |  | 1 | ,986^**^ | ,939^**^ |
| Day 28 (AM) |  |  |  |  |  |  |  |  |  |  |  |  | 1 | ,959^**^ |
| Day 28 (PM) |  |  |  |  |  |  |  |  |  |  |  |  |  | 1 |

**p<0.05, **p<0.001*

**S6 Table B.** Correlations for X from Day 1 (AM) to Day 31 (PM) (Second-Level Agenda) - **Continued**

| **Days** | **Day 29** | | **Day 30** | | **Day 31** | |
| --- | --- | --- | --- | --- | --- | --- |
|  | **AM** | **PM** | **AM** | **PM** | **AM** | **PM** |
| Day 1 (AM) | ,888^**^ | ,840^**^ | ,764^**^ | ,620^**^ | ,685^**^ | ,885^**^ |
| Day 1 (PM) | ,906^**^ | ,877^**^ | ,824^**^ | ,732^**^ | ,751^**^ | ,894^**^ |
| Day 2 (AM) | ,855^**^ | ,794^**^ | ,738^**^ | ,589^**^ | ,639^**^ | ,867^**^ |
| Day 2 (PM) | ,885^**^ | ,883^**^ | ,843^**^ | ,818^**^ | ,785^**^ | ,867^**^ |
| Day 3 (AM) | ,905^**^ | ,875^**^ | ,833^**^ | ,741^**^ | ,770^**^ | ,904^**^ |
| Day 3 (PM) | ,930^**^ | ,913^**^ | ,863^**^ | ,790^**^ | ,823^**^ | ,927^**^ |
| Day 4 (AM) | ,879^**^ | ,841^**^ | ,777^**^ | ,671^**^ | ,693^**^ | ,865^**^ |
| Day 4 (PM) | ,859^**^ | ,810^**^ | ,764^**^ | ,614^**^ | ,649^**^ | ,823^**^ |
| Day 5 (AM) | ,876^**^ | ,833^**^ | ,805^**^ | ,671^**^ | ,692^**^ | ,863^**^ |
| Day 5 (PM) | ,914^**^ | ,875^**^ | ,811^**^ | ,683^**^ | ,736^**^ | ,910^**^ |
| Day 6 (AM) | ,917^**^ | ,870^**^ | ,823^**^ | ,675^**^ | ,756^**^ | ,929^**^ |
| Day 6 (PM) | ,912^**^ | ,878^**^ | ,805^**^ | ,660^**^ | ,730^**^ | ,906^**^ |
| Day 7 (AM) | ,885^**^ | ,850^**^ | ,791^**^ | ,686^**^ | ,702^**^ | ,876^**^ |
| Day 7 (PM) | ,932^**^ | ,910^**^ | ,825^**^ | ,716^**^ | ,767^**^ | ,906^**^ |
| Day 8 (AM) | ,909^**^ | ,917^**^ | ,845^**^ | ,788^**^ | ,818^**^ | ,857^**^ |
| Day 8 (PM) | ,862^**^ | ,881^**^ | ,821^**^ | ,816^**^ | ,810^**^ | ,796^**^ |
| Day 9 (AM) | ,828^**^ | ,862^**^ | ,830^**^ | ,880^**^ | ,789^**^ | ,749^**^ |
| Day 9 (PM) | ,839^**^ | ,889^**^ | ,856^**^ | ,919^**^ | ,825^**^ | ,775^**^ |
| Day 10 (AM) | ,943^**^ | ,944^**^ | ,900^**^ | ,849^**^ | ,889^**^ | ,891^**^ |
| Day 10 (PM) | ,927^**^ | ,927^**^ | ,877^**^ | ,806^**^ | ,866^**^ | ,889^**^ |
| Day 11 (AM) | ,922^**^ | ,908^**^ | ,873^**^ | ,778^**^ | ,884^**^ | ,875^**^ |
| Day 11 (PM) | ,761^**^ | ,765^**^ | ,723^**^ | ,641^**^ | ,726^**^ | ,764^**^ |
| Day 12 (AM) | ,956^**^ | ,942^**^ | ,881^**^ | ,780^**^ | ,887^**^ | ,926^**^ |
| Day 12 (PM) | ,929^**^ | ,921^**^ | ,878^**^ | ,750^**^ | ,859^**^ | ,911^**^ |
| Day 13 (AM) | ,947^**^ | ,927^**^ | ,867^**^ | ,741^**^ | ,827^**^ | ,931^**^ |
| Day 13 (PM) | ,949^**^ | ,932^**^ | ,868^**^ | ,752^**^ | ,853^**^ | ,902^**^ |
| Day 14 (AM) | ,940^**^ | ,914^**^ | ,900^**^ | ,767^**^ | ,864^**^ | ,886^**^ |
| Day 14 (PM) | ,955^**^ | ,933^**^ | ,926^**^ | ,801^**^ | ,889^**^ | ,896^**^ |
| Day 15 (AM) | ,916^**^ | ,908^**^ | ,891^**^ | ,772^**^ | ,882^**^ | ,851^**^ |
| Day 15 (PM) | ,954^**^ | ,936^**^ | ,876^**^ | ,762^**^ | ,844^**^ | ,888^**^ |
| Day 16 (AM) | ,955^**^ | ,934^**^ | ,865^**^ | ,741^**^ | ,816^**^ | ,888^**^ |
| Day 16 (PM) | ,951^**^ | ,924^**^ | ,845^**^ | ,727^**^ | ,782^**^ | ,903^**^ |
| Day 17 (AM) | ,973^**^ | ,951^**^ | ,890^**^ | ,774^**^ | ,849^**^ | ,927^**^ |
| Day 17 (PM) | ,916^**^ | ,909^**^ | ,827^**^ | ,727^**^ | ,820^**^ | ,860^**^ |
| Day 18 (AM) | ,947^**^ | ,919^**^ | ,857^**^ | ,731^**^ | ,832^**^ | ,908^**^ |
| Day 18 (PM) | ,968^**^ | ,941^**^ | ,878^**^ | ,753^**^ | ,840^**^ | ,898^**^ |
| Day 19 (AM) | ,972^**^ | ,945^**^ | ,914^**^ | ,786^**^ | ,870^**^ | ,929^**^ |
| Day 19 (PM) | ,942^**^ | ,931^**^ | ,886^**^ | ,761^**^ | ,864^**^ | ,901^**^ |
| Day 20 (AM) | ,946^**^ | ,918^**^ | ,886^**^ | ,739^**^ | ,799^**^ | ,905^**^ |
| Day 20 (PM) | ,950^**^ | ,940^**^ | ,868^**^ | ,742^**^ | ,830^**^ | ,900^**^ |
| Day 21 (AM) | ,966^**^ | ,942^**^ | ,897^**^ | ,766^**^ | ,857^**^ | ,913^**^ |
| Day 21 (PM) | ,954^**^ | ,943^**^ | ,898^**^ | ,784^**^ | ,867^**^ | ,903^**^ |
| Day 22 (AM) | ,960^**^ | ,945^**^ | ,892^**^ | ,763^**^ | ,840^**^ | ,921^**^ |
| Day 22 (PM) | ,812^**^ | ,819^**^ | ,778^**^ | ,663^**^ | ,684^**^ | ,770^**^ |
| Day 23 (AM) | ,729^**^ | ,731^**^ | ,703^**^ | ,570^**^ | ,593^**^ | ,688^**^ |
| Day 23 (PM) | ,870^**^ | ,855^**^ | ,813^**^ | ,670^**^ | ,702^**^ | ,823^**^ |
| Day 24 (AM) | ,968^**^ | ,945^**^ | ,899^**^ | ,764^**^ | ,837^**^ | ,902^**^ |
| Day 24 (PM) | ,887^**^ | ,864^**^ | ,804^**^ | ,670^**^ | ,720^**^ | ,853^**^ |
| Day 25 (AM) | ,959^**^ | ,924^**^ | ,869^**^ | ,740^**^ | ,800^**^ | ,908^**^ |
| Day 25 (PM) | ,958^**^ | ,944^**^ | ,895^**^ | ,790^**^ | ,847^**^ | ,905^**^ |
| Day 26 (AM) | ,947^**^ | ,935^**^ | ,875^**^ | ,759^**^ | ,850^**^ | ,878^**^ |
| Day 26 (PM) | ,950^**^ | ,956^**^ | ,935^**^ | ,832^**^ | ,897^**^ | ,914^**^ |
| Day 27 (AM) | ,964^**^ | ,956^**^ | ,921^**^ | ,803^**^ | ,892^**^ | ,909^**^ |
| Day 27 (PM) | ,973^**^ | ,970^**^ | ,925^**^ | ,817^**^ | ,913^**^ | ,914^**^ |
| Day 28 (AM) | ,978^**^ | ,963^**^ | ,913^**^ | ,803^**^ | ,897^**^ | ,905^**^ |
| Day 28 (PM) | ,968^**^ | ,937^**^ | ,891^**^ | ,781^**^ | ,845^**^ | ,902^**^ |
| Day 29 (AM) | 1 | ,976^**^ | ,931^**^ | ,829^**^ | ,910^**^ | ,934^**^ |
| Day 29 (PM) |  | 1 | ,955^**^ | ,894^**^ | ,934^**^ | ,916^**^ |
| Day 30 (AM) |  |  | 1 | ,925^**^ | ,945^**^ | ,877^**^ |
| Day 30 (PM) |  |  |  | 1 | ,924^**^ | ,756^**^ |
| Day 31 (AM) |  |  |  |  | 1 | ,853^**^ |
| Day 31 (PM) |  |  |  |  |  | 1 |

**p<0.05, **p<0.001*

**S6 Table C.** Relationships between X (AM) and Online News (PM), and between Online News (AM) and X (PM) (Intraday Relationships) - (Second-Level Agenda)

| **Days** | **X (AM) – Online News (PM)** | | **Online News (AM) – X (PM)** | |
| --- | --- | --- | --- | --- |
|  | **r** | **p** | **r** | **p** |
| Day 1 | 0.441 | 0.000* | 0.502 | 0.000* |
| Day 2 | 0.407 | 0.000* | 0.583 | 0.000* |
| Day 3 | 0.482 | 0.000* | 0.424 | 0.000* |
| Day 4 | 0.503 | 0.000* | 0.435 | 0.000* |
| Day 5 | 0.333 | 0.000* | 0.502 | 0.000* |
| Day 6 | 0.491 | 0.000* | 0.549 | 0.000* |
| Day 7 | 0.444 | 0.000* | 0.591 | 0.000* |
| Day 8 | 0.585 | 0.000* | 0.684 | 0.000* |
| Day 9 | 0.416 | 0.000* | 0.459 | 0.000* |
| Day 10 | 0.554 | 0.000* | 0.650 | 0.000* |
| Day 11 | 0.724 | 0.000* | 0.621 | 0.000* |
| Day 12 | 0.745 | 0.000* | 0.642 | 0.000* |
| Day 13 | 0.576 | 0.000* | 0.668 | 0.000* |
| Day 14 | 0.735 | 0.000* | **0.818** | 0.000* |
| Day 15 | **0.801** | 0.000* | 0.672 | 0.000* |
| Day 16 | 0.637 | 0.000* | 0.562 | 0.000* |
| Day 17 | 0.617 | 0.000* | 0.793 | 0.000* |
| Day 18 | 0.702 | 0.000* | 0.684 | 0.000* |
| Day 19 | 0.656 | 0.000* | 0.656 | 0.000* |
| Day 20 | 0.671 | 0.000* | 0.690 | 0.000* |
| Day 21 | 0.655 | 0.000* | 0.782 | 0.000* |
| Day 22 | 0.654 | 0.000* | 0.615 | 0.000* |
| Day 23 | 0.473 | 0.000* | 0.462 | 0.000* |
| Day 24 | 0.630 | 0.000* | 0.492 | 0.000* |
| Day 25 | 0.646 | 0.000* | 0.663 | 0.000* |
| Day 26 | 0.740 | 0.000* | 0.783 | 0.000* |
| Day 27 | 0.746 | 0.000* | 0.745 | 0.000* |
| Day 28 | 0.702 | 0.000* | 0.673 | 0.000* |
| Day 29 | 0.739 | 0.000* | 0.744 | 0.000* |
| Day 30 | 0.598 | 0.000* | 0.700 | 0.000* |
| Day 31 | 0.599 | 0.000* | 0.554 | 0.000* |

**p<0.05*

**S6 Table D.** Relationships between X (PM) and Online News (AM), and between Online News (PM) and X (AM) (Day-to-Day Relationships) - (Second-Level Agenda)

| **Days** | **X (PM) – Online News (AM)** | | **Online News (PM) – X (AM)** | |
| --- | --- | --- | --- | --- |
|  | **r** | **p** | **r** | **p** |
| Days 1 and 2 | 0.598 | 0.000* | 0.377 | 0.000* |
| Days 2 and 3 | 0.350 | 0.000* | 0.467 | 0.000* |
| Days 3 and 4 | 0.555 | 0.000* | 0.414 | 0.000* |
| Days 4 and 5 | 0.406 | 0.000* | 0.510 | 0.000* |
| Days 5 and 6 | 0.540 | 0.000* | 0.435 | 0.000* |
| Days 6 and 7 | 0.548 | 0.000* | 0.386 | 0.000* |
| Days 7 and 8 | 0.620 | 0.000* | 0.702 | 0.000* |
| Days 8 and 9 | 0.690 | 0.000* | 0.293 | 0.000* |
| Days 9 and 10 | 0.474 | 0.000* | 0.553 | 0.000* |
| Days 10 and 11 | 0.754 | 0.000* | 0.668 | 0.000* |
| Days 11 and 12 | 0.620 | 0.000* | 0.674 | 0.000* |
| Days 12 and 13 | 0.650 | 0.000* | 0.690 | 0.000* |
| Days 13 and 14 | 0.801 | 0.000* | 0.597 | 0.000* |
| Days 14 and 15 | 0.730 | 0.000* | **0.798** | 0.000* |
| Days 15 and 16 | 0.639 | 0.000* | 0.713 | 0.000* |
| Days 16 and 17 | 0.661 | 0.000* | 0.666 | 0.000* |
| Days 17 and 18 | 0.749 | 0.000* | 0.628 | 0.000* |
| Days 18 and 19 | 0.589 | 0.000* | 0.677 | 0.000* |
| Days 19 and 20 | 0.684 | 0.000* | 0.647 | 0.000* |
| Days 20 and 21 | 0.760 | 0.000* | 0.738 | 0.000* |
| Days 21 and 22 | 0.764 | 0.000* | 0.635 | 0.000* |
| Days 22 and 23 | 0.545 | 0.000* | 0.457 | 0.000* |
| Days 23 and 24 | 0.487 | 0.000* | 0.622 | 0.000* |
| Days 24 and 25 | 0.515 | 0.000* | 0.568 | 0.000* |
| Days 25 and 26 | 0.708 | 0.000* | 0.756 | 0.000* |
| Days 26 and 27 | 0.720 | 0.000* | 0.771 | 0.000* |
| Days 27 and 28 | 0.767 | 0.000* | 0.754 | 0.000* |
| Days 28 and 29 | 0.666 | 0.000* | 0.682 | 0.000* |
| Days 29 and 30 | **0.853** | 0.000* | 0.728 | 0.000* |
| Days 30 and 31 | 0.492 | 0.000* | 0.656 | 0.000* |

**p<0.05*

**Appendix S7. RCb Results for First-Level and Second-Level Agenda Setting**

**S7 Table A:** Intraday RCb Results for First-Level Agenda Setting

| **PX1Y1** | **PX2Y2** | **PX1X2** | **PY1Y2** | **RCb** |
| --- | --- | --- | --- | --- |
| 0,895 | 0,893 | 0,99 | 0,991 | 0,88550711 |
| 0,892 | 0,921 | 0,99 | 0,99 | 0,897435 |
| 0,914 | 0,936 | 0,991 | 0,99 | 0,91621262 |
| 0,912 | 0,888 | 0,994 | 0,989 | 0,89235284 |
| 0,899 | 0,9 | 0,982 | 0,979 | 0,88196078 |
| 0,913 | 0,901 | 0,991 | 0,974 | 0,89116085 |
| 0,906 | 0,931 | 0,978 | 0,99 | 0,9038208 |
| 0,956 | 0,934 | 0,994 | 0,963 | 0,92479851 |
| 0,905 | 0,924 | 0,994 | 0,959 | 0,89315264 |
| 0,942 | 0,93 | 0,987 | 0,978 | 0,91962965 |
| 0,935 | 0,944 | 0,957 | 0,981 | 0,91044531 |
| 0,946 | 0,943 | 0,989 | 0,984 | 0,93175224 |
| 0,923 | 0,931 | 0,981 | 0,983 | 0,91031447 |
| 0,942 | 0,943 | 0,993 | 0,979 | 0,92932842 |
| 0,958 | 0,948 | 0,991 | 0,986 | 0,94204351 |
| 0,967 | 0,948 | 0,993 | 0,984 | 0,94649856 |
| 0,959 | 0,93 | 0,976 | 0,985 | 0,926092 |
| 0,952 | 0,919 | 0,98 | 0,986 | 0,91960078 |
| 0,928 | 0,933 | 0,994 | 0,972 | 0,91473877 |
| 0,95 | 0,949 | 0,996 | 0,985 | 0,94049425 |
| 0,954 | 0,962 | 0,994 | 0,994 | 0,952252 |
| 0,961 | 0,916 | 0,982 | 0,989 | 0,92489758 |
| 0,923 | 0,943 | 0,987 | 0,987 | 0,920871 |
| 0,961 | 0,945 | 0,983 | 0,983 | 0,936799 |
| 0,951 | 0,926 | 0,995 | 0,986 | 0,92959384 |
| 0,953 | 0,944 | 0,993 | 0,989 | 0,93996541 |
| 0,953 | 0,948 | 0,996 | 0,987 | 0,94243046 |
| 0,966 | 0,954 | 0,994 | 0,989 | 0,95184303 |
| 0,973 | 0,955 | 0,995 | 0,995 | 0,95918 |
| 0,95 | 0,944 | 0,997 | 0,981 | 0,93661364 |
| 0,95 | 0,942 | 0,979 | 0,948 | 0,91158894 |

**S7 Table B:** Inter-Day RCb Results for First-Level Agenda Setting

| **PX1Y1** | **PX2Y2** | **PX1X2** | **PY1Y2** | **RCb** |
| --- | --- | --- | --- | --- |
| 0,892 | 0,921 | 0,989 | 0,99 | 0,89698186 |
| 0,914 | 0,936 | 0,996 | 0,991 | 0,91899041 |
| 0,912 | 0,888 | 0,987 | 0,986 | 0,88785011 |
| 0,899 | 0,9 | 0,982 | 0,986 | 0,88510983 |
| 0,913 | 0,901 | 0,994 | 0,957 | 0,88493759 |
| 0,906 | 0,931 | 0,99 | 0,992 | 0,91023396 |
| 0,956 | 0,934 | 0,97 | 0,987 | 0,92471739 |
| 0,905 | 0,924 | 0,993 | 0,966 | 0,89583782 |
| 0,942 | 0,93 | 0,992 | 0,984 | 0,92477558 |
| 0,935 | 0,944 | 0,982 | 0,978 | 0,92071192 |
| 0,946 | 0,943 | 0,982 | 0,982 | 0,927499 |
| 0,923 | 0,931 | 0,993 | 0,985 | 0,9168105 |
| 0,942 | 0,943 | 0,988 | 0,975 | 0,92508404 |
| 0,958 | 0,948 | 0,989 | 0,991 | 0,94347048 |
| 0,967 | 0,948 | 0,995 | 0,983 | 0,94698493 |
| 0,959 | 0,93 | 0,991 | 0,988 | 0,93458382 |
| 0,952 | 0,919 | 0,971 | 0,987 | 0,91588508 |
| 0,928 | 0,933 | 0,995 | 0,984 | 0,92074397 |
| 0,95 | 0,949 | 0,993 | 0,976 | 0,93481759 |
| 0,954 | 0,962 | 0,997 | 0,987 | 0,95034807 |
| 0,961 | 0,916 | 0,992 | 0,99 | 0,93005397 |
| 0,923 | 0,943 | 0,99 | 0,99 | 0,92367 |
| 0,961 | 0,945 | 0,995 | 0,989 | 0,94538032 |
| 0,951 | 0,926 | 0,991 | 0,985 | 0,92724227 |
| 0,953 | 0,944 | 0,994 | 0,983 | 0,93760676 |
| 0,953 | 0,948 | 0,997 | 0,988 | 0,94338095 |
| 0,966 | 0,954 | 0,998 | 0,994 | 0,95616193 |
| 0,973 | 0,955 | 0,994 | 0,989 | 0,95580904 |
| 0,95 | 0,944 | 0,996 | 0,992 | 0,94131991 |
| 0,95 | 0,942 | 0,969 | 0,953 | 0,9091375 |

**S7 Table C:** Intraday RCb Results for Second-Level Agenda Setting

| **PX1Y1** | **PX2Y2** | **PX1X2** | **PY1Y2** | **RCb** |
| --- | --- | --- | --- | --- |
| 0,504 | 0,453 | 0,974 | 0,965 | 0,46391075 |
| 0,528 | 0,458 | 0,915 | 0,914 | 0,45084857 |
| 0,353 | 0,545 | 0,986 | 0,891 | 0,42192588 |
| 0,432 | 0,515 | 0,973 | 0,852 | 0,43301737 |
| 0,416 | 0,445 | 0,976 | 0,927 | 0,40975652 |
| 0,534 | 0,498 | 0,968 | 0,901 | 0,48251174 |
| 0,424 | 0,609 | 0,966 | 0,908 | 0,48419224 |
| 0,714 | 0,627 | 0,977 | 0,735 | 0,57965375 |
| 0,416 | 0,475 | 0,977 | 0,817 | 0,40119965 |
| 0,581 | 0,698 | 0,93 | 0,763 | 0,54396402 |
| 0,772 | 0,631 | 0,807 | 0,881 | 0,59263466 |
| 0,661 | 0,767 | 0,952 | 0,918 | 0,66770034 |
| 0,593 | 0,65 | 0,959 | 0,85 | 0,56316628 |
| 0,77 | 0,785 | 0,972 | 0,895 | 0,72641326 |
| 0,766 | 0,766 | 0,923 | 0,905 | 0,70015794 |
| 0,605 | 0,633 | 0,968 | 0,888 | 0,57496537 |
| 0,714 | 0,717 | 0,93 | 0,914 | 0,65971583 |
| 0,663 | 0,727 | 0,969 | 0,872 | 0,64063489 |
| 0,57 | 0,722 | 0,958 | 0,778 | 0,56373411 |
| 0,586 | 0,771 | 0,947 | 0,894 | 0,62481801 |
| 0,743 | 0,729 | 0,966 | 0,941 | 0,7018363 |
| 0,712 | 0,542 | 0,809 | 0,94 | 0,54984736 |
| 0,45 | 0,49 | 0,887 | 0,921 | 0,42495512 |
| 0,628 | 0,508 | 0,93 | 0,88 | 0,5142361 |
| 0,591 | 0,706 | 0,962 | 0,909 | 0,60691511 |
| 0,778 | 0,754 | 0,937 | 0,962 | 0,72738002 |
| 0,723 | 0,772 | 0,986 | 0,944 | 0,72150828 |
| 0,759 | 0,666 | 0,959 | 0,92 | 0,66953792 |
| 0,709 | 0,765 | 0,976 | 0,954 | 0,7112512 |
| 0,792 | 0,614 | 0,925 | 0,814 | 0,61250244 |
| 0,598 | 0,592 | 0,853 | 0,971 | 0,54377434 |

**S7 Table D:** Inter-Day RCb Results for Second-Level Agenda Setting

| **PX1Y1** | **PX2Y2** | **PX1X2** | **PY1Y2** | **RCb** |
| --- | --- | --- | --- | --- |
| 0,528 | 0,458 | 0,967 | 0,925 | 0,4664929 |
| 0,353 | 0,545 | 0,973 | 0,919 | 0,42492697 |
| 0,432 | 0,515 | 0,959 | 0,889 | 0,43782776 |
| 0,416 | 0,445 | 0,983 | 0,869 | 0,39939752 |
| 0,534 | 0,498 | 0,986 | 0,914 | 0,49055184 |
| 0,424 | 0,609 | 0,968 | 0,932 | 0,49076307 |
| 0,714 | 0,627 | 0,922 | 0,94 | 0,62426467 |
| 0,416 | 0,475 | 0,901 | 0,923 | 0,40632555 |
| 0,581 | 0,698 | 0,912 | 0,803 | 0,5494777 |
| 0,772 | 0,631 | 0,936 | 0,856 | 0,62917003 |
| 0,661 | 0,767 | 0,856 | 0,923 | 0,63555325 |
| 0,593 | 0,65 | 0,967 | 0,878 | 0,57400042 |
| 0,77 | 0,785 | 0,957 | 0,842 | 0,7007887 |
| 0,766 | 0,766 | 0,966 | 0,9 | 0,7151249 |
| 0,605 | 0,633 | 0,966 | 0,931 | 0,58722142 |
| 0,714 | 0,717 | 0,976 | 0,925 | 0,68032745 |
| 0,663 | 0,727 | 0,933 | 0,943 | 0,65191926 |
| 0,57 | 0,722 | 0,964 | 0,879 | 0,59592178 |
| 0,586 | 0,771 | 0,944 | 0,856 | 0,61137933 |
| 0,743 | 0,729 | 0,974 | 0,963 | 0,71282749 |
| 0,712 | 0,542 | 0,965 | 0,895 | 0,5835228 |
| 0,45 | 0,49 | 0,965 | 0,953 | 0,45073882 |
| 0,628 | 0,508 | 0,946 | 0,924 | 0,53111675 |
| 0,591 | 0,706 | 0,943 | 0,922 | 0,60476459 |
| 0,778 | 0,754 | 0,968 | 0,933 | 0,72820639 |
| 0,723 | 0,772 | 0,967 | 0,946 | 0,71502683 |
| 0,759 | 0,666 | 0,986 | 0,964 | 0,69473171 |
| 0,709 | 0,765 | 0,968 | 0,948 | 0,70608446 |
| 0,792 | 0,614 | 0,955 | 0,92 | 0,65917731 |
| 0,598 | 0,592 | 0,924 | 0,718 | 0,4923243 |
